# Pharmacological inhibition of the LIF/LIFR autocrine loop reveals vulnerability of ovarian cancer cells to ferroptosis

**DOI:** 10.1038/s41698-024-00612-y

**Published:** 2024-05-24

**Authors:** Behnam Ebrahimi, Suryavathi Viswanadhapalli, Uday P. Pratap, Gopalam Rahul, Xue Yang, Prabhakar Pitta Venkata, Viktor Drel, Bindu Santhamma, Swapna Konda, Xiaonan Li, Alondra Lee Rodriguez Sanchez, Hui Yan, Gangadhara R. Sareddy, Zhenming Xu, Brij B. Singh, Philip T. Valente, Yidong Chen, Zhao Lai, Manjeet Rao, Edward R. Kost, Tyler Curiel, Rajeshwar R. Tekmal, Hareesh B. Nair, Ratna K. Vadlamudi

**Affiliations:** 1 Department of Obstetrics and Gynecology, University of Texas Health San Antonio, San Antonio, TX 78229 USA; 2 Mays Cancer Center, University of Texas Health San Antonio, San Antonio, TX 78229 USA; 3 Department of Obstetrics and Gynecology, Second Xiangya Hospital, Central South University, Changsha, Hunan 410011 PR China; 4 Greehey Children’s Cancer Research Institute, University of Texas Health San Antonio, San Antonio, TX 78229 USA; 5 Department of Periodontics, University of Texas Health San Antonio, San Antonio, TX 78229 USA; 6 Evestra Inc, San Antonio, TX USA; 7 Department of microbiology and immunology, University of Texas Health San Antonio, San Antonio, TX 78229 USA; 8 Department of Pathology, University of Texas Health San Antonio, San Antonio, TX 78229 USA; 9 Department of Population Sciences, University of Texas Health San Antonio, San Antonio, TX 78229 USA; 10 Department of Microbiology and Immunology, Geisel School of Medicine, Dartmouth, NH 03755 USA; 11 Audie L. Murphy Division, South Texas Veterans Health Care System, San Antonio, TX 78229 USA

**Keywords:** Molecular medicine, Ovarian cancer

## Abstract

Of all gynecologic cancers, epithelial-ovarian cancer (OCa) stands out with the highest mortality rates. Despite all efforts, 90% of individuals who receive standard surgical and cytotoxic therapy experience disease recurrence. The precise mechanism by which leukemia inhibitory factor (LIF) and its receptor (LIFR) contribute to the progression of OCa remains unknown. Analysis of cancer databases revealed that elevated expression of LIF or LIFR was associated with poor progression-free survival of OCa patients and a predictor of poor response to chemotherapy. Using multiple primary and established OCa cell lines or tissues that represent five subtypes of epithelial-OCa, we demonstrated that LIF/LIFR autocrine signaling is active in OCa. Moreover, treatment with LIFR inhibitor, EC359 significantly reduced OCa cell viability and cell survival with an IC_50_ ranging from 5-50 nM. Furthermore, EC359 diminished the stemness of OCa cells. Mechanistic studies using RNA-seq and rescue experiments unveiled that EC359 primarily induced ferroptosis by suppressing the glutathione antioxidant defense system. Using multiple in vitro, ex vivo and in vivo models including cell-based xenografts, patient-derived explants, organoids, and xenograft tumors, we demonstrated that EC359 dramatically reduced the growth and progression of OCa. Additionally, EC359 therapy considerably improved tumor immunogenicity by robust CD45^+^ leukocyte tumor infiltration and polarizing tumor-associated macrophages (TAMs) toward M1 phenotype while showing no impact on normal T-, B-, and other immune cells. Collectively, our findings indicate that the LIF/LIFR autocrine loop plays an essential role in OCa progression and that EC359 could be a promising therapeutic agent for OCa.

## Introduction

The most fatal gynecologic cancer in the United States is ovarian cancer (OCa)^1^. OCa is highly heterogeneous, and the majority of OCa are from epithelial origin with five histological subgroups, including high-grade serous (HGSOC), low-grade serous (LGSOC), clear cell (CCOC), endometrioid (ENOC), and mucinous (MOC)^2^. The majority of OCa patients are diagnosed with advanced-stage disease^3^ and HGSOC accounts for more than 70% of all epithelial OCa cases^4^. The current standard of care for OCa includes a combination of surgery and cytotoxic therapy. Unfortunately, around 90% of patients experience recurrence and ultimately pass away from chemotherapy-resistant disease^5^. Furthermore, the removal of OCa stem cells is essential for the creation of effective therapeutic approaches because they are linked to tumor recurrence and therapy resistance. The long-term outlook for OCa is poor since there are few effective treatments available right now and therefore, identification of new therapeutic targets is urgently needed.

Leukemia inhibitory factor (LIF) is the most pleiotropic member of the interleukin-6 family of cytokines^6^. The LIFR complex, which consists of LIFR and glycoprotein 130 (gp130), transmits LIF signals^7^ through the downstream pathways including JAK/STAT3 as the immediate effectors and concurrent MAPK, AKT, and mTOR, that are associated with the progression of OCa^7–9^. Oncostatin M (OSM), another LIFR ligand, also interacts with LIFR to activate its downstream signaling^10^. The emerging evidence has shown oncogenic functions for LIF/LIFR pathway in many solid cancers that has recently attracted considerable attention^11–14^. However, it is not clear how essential LIFR is as a therapeutic target or how blocking LIFR will alter the course of OCa.

LIF signaling has also been demonstrated to have extrinsic effects in the tumor microenvironment in addition to its tumor intrinsic effects. For instance, LIF signaling promotes activation of stromal fibroblasts and pro-invasive interactions between tumor cells and fibroblasts^15^. In OCa patients, LIF is secreted to the peritoneal cavity by OCa cells and is shown to contribute to immunological deficiencies within the tumors^16,17^. Moreover, LIF/LIFR signaling was shown to be involved in modifying the effector T cells, regulatory T cells, macrophages^17^, and myeloid cells found in the tumor microenvironment (TME), which results in immune suppression^18^. Currently, it remains unknown whether the autocrine LIF/LIFR loop, other tumor intrinsic, as well as external effects of the LIF/LIFR axis drive the progression of OCa.

In this study, we examined whether LIF/LIFR signaling is essential for the progression of OCa and whether inhibiting LIF/LIFR signaling with the recently developed LIFR inhibitor, EC359 will have a therapeutic effect on OCa cells. Our findings, derived from the analysis of public cancer databases alongside 32 established, primary OCa cell lines, and tumor tissues reveal the existence of an autocrine loop involving LIF/LIFR. LIFR inhibition enhanced cell death of OCa cells through ferroptosis. Furthermore, we presented proof that inhibiting LIFR signaling lowers stemness and increases anti-tumor immunity. Moreover, we provided evidence that LIFR inhibitor EC359 inhibited the growth of OCa cells in vitro and tumor progression in cell-derived xenografts (CDX) and patient-derived xenograft (PDX) models. These findings collectively signify EC359 potential as a valuable drug for further preclinical and clinical investigations.

## Results

### LIFR ligands are highly expressed in OCa and their levels correlate with poor survival of OCa patients

**Fig. 1:**
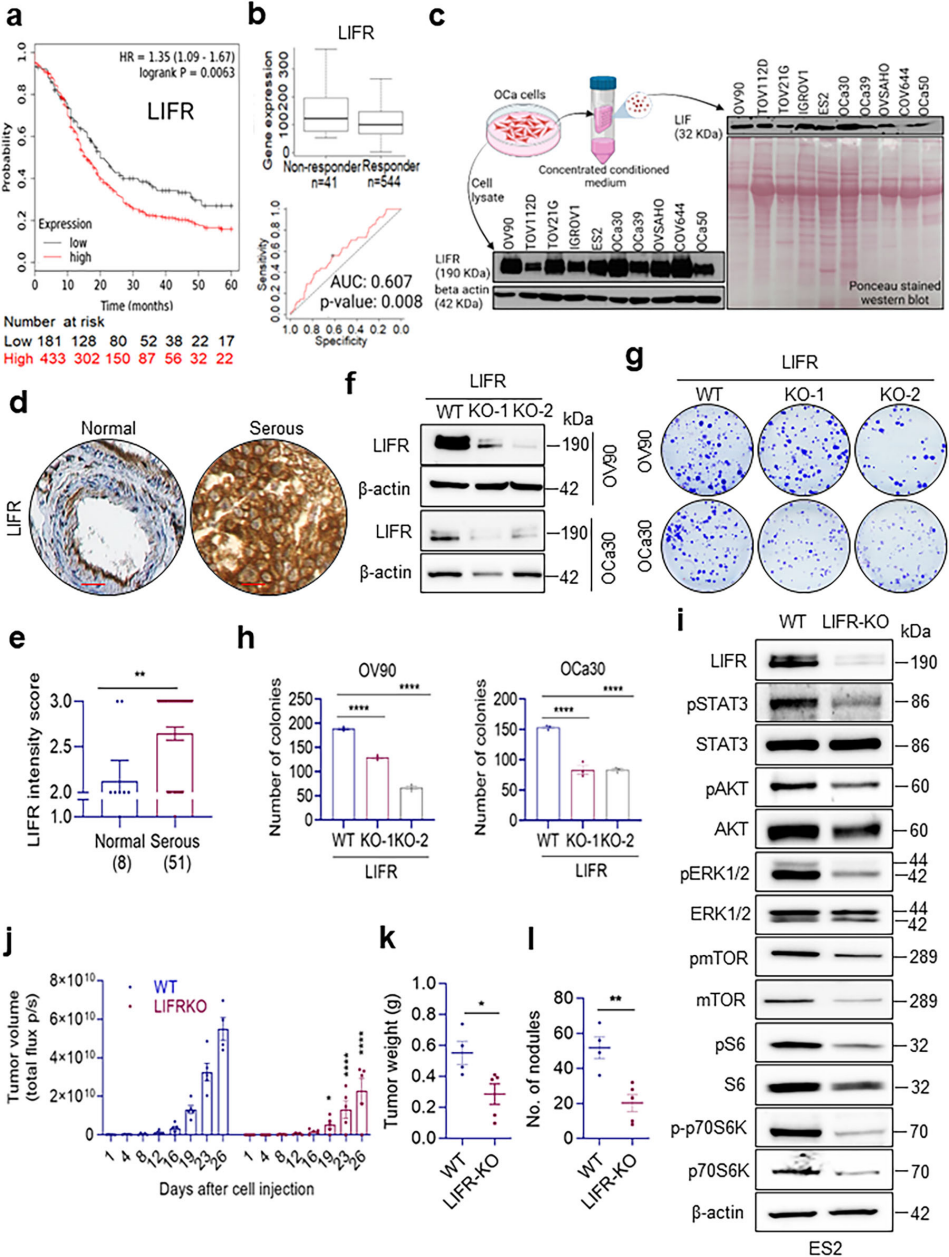
OCa upregulates an autocrine loop of LIF/LIFR. **a** Kaplan-Meier survival analysis of OCa stratified by LIFR gene expression levels. **b** Box plots and ROC curves of LIFR were generated using progression free survival (PFS) at 6-month cohort. Only samples with serous histology (grade 3) and those treated with platinum and taxane combined therapy were included in the analysis. **c** Western blot analysis of concentrated conditioned media of OCa cells cultured in serum free RPMI-1640 demonstrating the presence of LIF and total cellular lysates of OCa cells cultured in RPMI-1640 supplemented with 10% FBS showing LIFR expression. Ponceau stained nitrocellulose membrane is shown as loading control for the conditioned media. **d** Representative IHC images of LIFR expression in normal and serous OCa on OCa tissue array (OVC2281, TissueArray.Com LLC) showing higher expression of LIFR in OCa compared to normal tissues of the ovary. Scale bar represents 100 µm. **e** Quantitation of LIFR expression in normal (*n* = 8) and serous OCa (*n* = 51) from OCa tissue array. Data presented as mean ± S.E.M., significance was determined by Two-tailed Unpaired *t* test. **f** Representation of expression of LIFR in OV90, and OCa30-WT cells, stably expressing LIFR targeting sgRNA-1 and 2. **g** The effect of LIFR-KO on the long-term colony formation in OV90 and OCa30 cell lines. **h** Bar chart represents quantification of the colonies. Data presented as mean ± S.E.M., *n* = 3 biologically independent samples. Significance was determined by one-way ANOVA followed by Uncorrected Fisher’s LSD. **i** Western blot analysis of the LIFR-KO and WT ES2 cells presenting inhibitory effect of LIFR-KO on LIFR downstream pathways including STAT3, AKT, ERK, and mTOR. Uncropped blots are provided. **j** ES2-WT and LIFR-KO cells (1 × 10^5^) labeled with luciferase were injected into the peritoneal cavity of female SCID mice. Tumor progression was monitored using Xenogen imaging system. **k** and **l** display the tumor weight and number of nodules respectively. Data presented as mean ± S.E.M., For tumor volume data, significance was determined by two-way ANOVA followed by Uncorrected Fisher’s LSD. For tumor weight and number of nodules data, *n* = 4 mice for WT and 5 mice for LIFR-KO; significance was determined by Two-tailed Unpaired *t* test. Experiments shown in (**c**, **f**, **i**, **j**, **k**, **l**) were done once. Numerical source data for (**e**, **h**, **j**, **k**, **l**) are provided. *p < 0.05, **p < 0.01, ****p < 0.0001.

To examine the association of LIF, OSM, and LIFR expression with survival of OCa patients, we used the Kaplan-Meier survival analysis tool (KMplot)^19^. The increased expression of LIFR, and its ligands (LIF, and OSM) was associated with poor progression-free survival (PFS) in OCa patients (Fig. 1 a, Supplementary Fig. 1a). To further evaluate the relevance of LIFR axis in chemotherapy resistance, we compared the expression of LIFR and its ligands LIF, and OSM in OCa that were treated with platinum or taxane chemotherapy using receiver operation characteristics (ROC) plotter, which links gene expression and response to therapy using transcriptomel data of OCa patients^19^. However, this database has limited number of tumor samples from clear cell, mucinous, and endometrioid OCa subtypes, hence, our analyses used only serous subtype of OCa. The results showed that expression levels of LIFR, and its ligands (LIF, and OSM) were significantly higher in chemotherapy non-responders when compared to responders (Fig. 1b, Supplementary Fig. 1b). To further examine whether alterations occur in the levels of LIFR ligands (LIF and OSM) in OCa, we examined their status using TNMplot analysis platform that enables comparison of gene expression between tumor and normal tissues using validated data bases^20^. Results showed that both LIFR ligands (LIF and OSM) are highly expressed in OCa compared to normal tissues (Supplementary Fig. 1c). These results suggest that LIFR ligands are highly expressed in OCa and LIFR autocrine loop may play a role in chemotherapy response.

### OCa cells exhibit LIF/LIFR autocrine signaling and LIFR is required for optimal growth of OCa in vitro and in vivo

To confirm existence of autocrine loop via secretion of LIF into the medium by OCa cells, we plated several established cell lines (OV90, TOV112D, TOV21G, IGROV1, ES2, OVSAHO, COV644) and primary OCa cell lines (OCa30, OCa39, OCa50), and let them grow to 70-80% confluency. Cells were washed with PBS and then cultured in no serum containing medium for 48 h, medium was collected, concentrated, and analyzed for the presence of LIF. Results showed detectable levels of LIF in the medium of all the OCa cell lines tested (Fig. 1c). We also confirmed expression of LIFR in these models using Western blotting of total lysates. Results showed detectable expression of LIFR in the OCa models tested (Fig. 1c). We also validated the expression of LIFR in human OCa tumors using tissue microarray. IHC results confirmed higher expression of LIFR in serous OCa (Fig. 1d, e) compared to the normal tissue. To provide genetic evidence that intrinsic LIF/LIFR signaling in OCa epithelial cells will benefit OCa progression, we generated LIFR knockout (KO) cells using CRISPR/Cas9 (Supplementary Fig. 1d**)**. Western blot analysis confirmed LIFR-KO in OCa cells (Fig. 1f, i). In biological assays, OV90 and OCa30 LIFR-KO cells showed reduced colony formation ability compared to the cells expressing vector (Fig. 1g, h). Further, Western blotting analyses using phospho-specific antibodies showed that LIFR-KO cells exhibit substantial reduction in LIF/LIFR downstream signaling pathways (Fig. 1i, Supplementary Fig. 1e). The changes in the LIF/LIFR downstream signaling pathways are not likely due to cell cycle alterations, as we did not see major changes in the levels of p21 and p27 in these clones (Supplementary Fig. 1e). To test the significance of LIFR signaling in the progression of OCa, ES2-vector or ES2-LIFR-KO cells (1 × 10^5^) were injected i.p. into the SCID mice and tumor growth was monitored. LIFR-KO resulted in significant reduction in tumor volume (~58% reduction), tumor weights and tumor nodules (Fig. 1j–l). Together, these data delineate that OCa cells secrete significant amount of LIF and express high levels of LIFR, suggesting a potential autocrine loop of LIF/LIFR that is needed for optimal growth of OCa.

### EC359 inhibits LIF/LIFR autocrine signaling

**Fig. 2:**
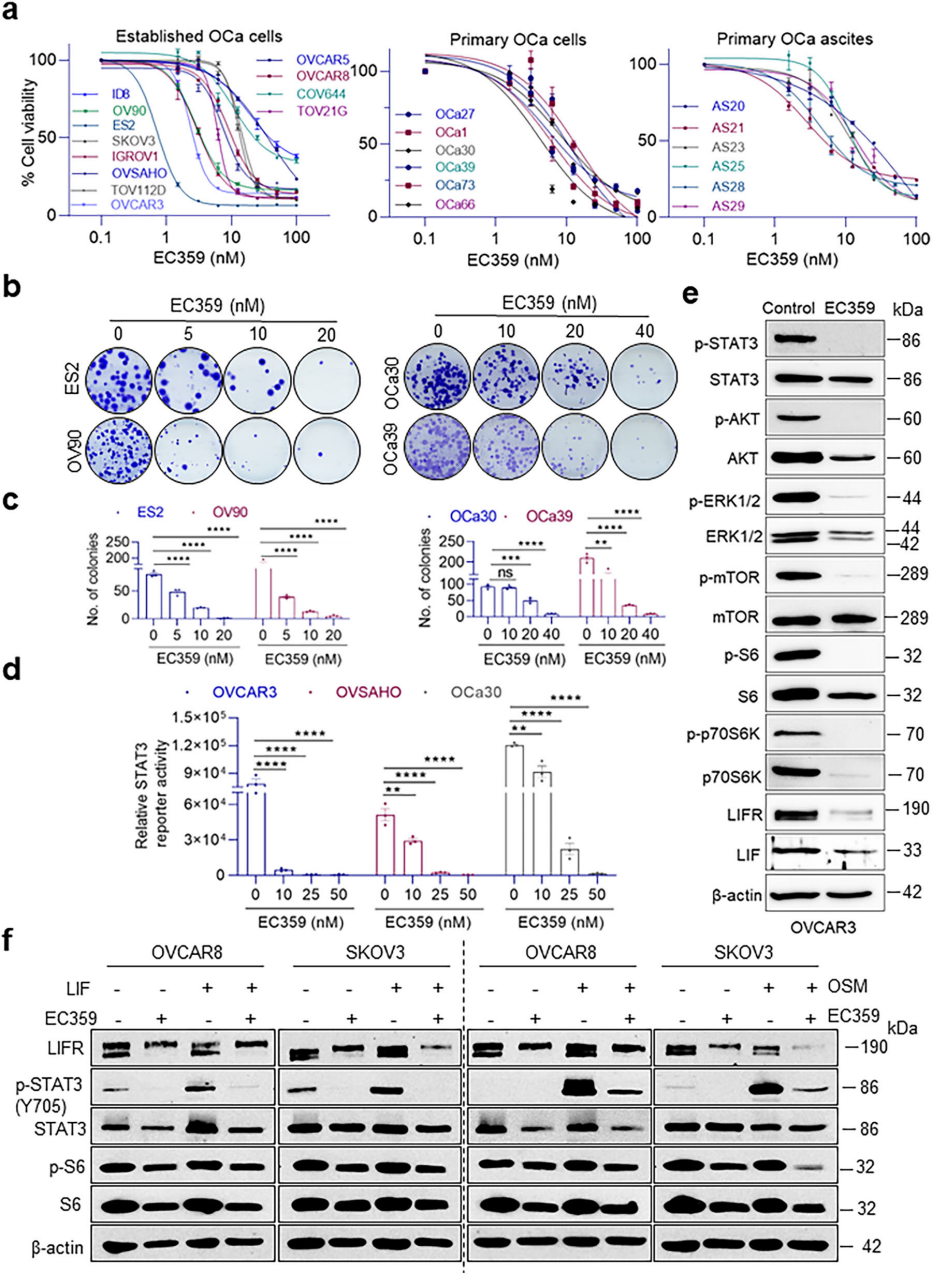
EC359 inhibits proliferation of OCa cells and LIFR downstream signaling. **a** Effect of EC359 treatment for four days on cell viability of different established and primary OCa cell lines isolated from solid tumor tissues or ascites of patients. Data presented as mean ± S.E.M., *n* = 3 biologically independent samples. **b** Images of the effect of different doses of EC359 on long-term clonogenic potential of OCa cells when 500 cells (ES2 and OV90) or 200 cells (OCa30 and OCa39) were treated with vehicle or EC359 for 4 days and then cultured for 10 days without the inhibitor. **c** represents quantification of colonies of ES2, OV90, OCa30, and OCa39 cells treated with vehicle or EC359. Data presented as mean ± S.E.M., *n* = 3 biologically independent samples. **d** STAT3 reporter assay displaying inhibitory effect of EC359 on the activity of STAT3 reporter in OVCAR3, OVSAHO, and OCa30 cell lines. Data presented as mean ± S.E.M., *n* = 3 biologically independent samples. For Fig, (**c**, **d**), significance was determined by two-way ANOVA followed by Uncorrected Fisher’s LSD. **e** Inhibitory effect of EC359 (60 nM) for 6 h on activation of LIFR-downstream signaling including STAT3, AKT, ERK, and mTOR on OVCAR3 cells determined by western blotting. **f** OVCAR8 and SKOV3 cells were serum starved for 24 h, pretreated with ± EC359 100 nM for 1 h and then treated with LIF (100 ng/ml) or OSM (10 ng/ml) for 10 h. LIFR downstream signaling was analyzed by western blotting. Western blots in each panel are derived from the same experiment and processed in parallel. Western blot experiments were repeated twice independently, with similar results. Numerical source data for (**a**, **c**, **d**) are provided. ns, not significant; **p < 0.01, ***p < 0.001, ****p < 0.0001.

We then performed cell viability assays using 24 OCa cell lines representing four different subtypes of OCa including 12 established, six primary tumor derived and six ascites-derived OCa cell lines to see if addition of LIFR inhibitor EC359 blocks LIF/LIFR autocrine signaling (Fig. 2a). With an IC_50_ of 5–50 nM, LIFR inhibitor EC359 significantly reduced the cell viability of 24 OCa cell lines. Using three normal model cell lines including human nontumorigenic immortalized ovarian surface epithelial cells (IOSE-80), human endometrial stromal cells (HESC), human epithelial kidney cells (HEK-293T), we found that EC359 has limited activity in these cell lines compared with OCa cells, thus indicating a potential therapeutic window for EC359 in treating OCa (Supplementary Fig. 1f). Similarly, EC359 treatment substantially reduced OCa cells colony formation ability (Fig. 2b, c). Furthermore, using multiple OCa cell lines stably expressing STAT3-Luciferase reporter, we demonstrated dose dependent reduction of STAT3 reporter activity (Fig. 2d). Accordingly, treatment of OCa cells with EC359 considerably reduced the activation of LIFR downstream signaling activated by its autocrine signaling (Fig. 2e) or when recombinant ligands (LIF and OSM) were added to the medium (Fig. 2f). Collectively, these results suggest that OCa cells exhibit LIF/LIFR autocrine loop and EC359 blocks this positive autocrine loop and that it potently reduces the growth of OCa cells in vitro.

### EC359 is more effective in blocking LIFR signaling in OCa compared to anti LIF antibody and STAT3/JAK inhibitors

**Fig. 3:**
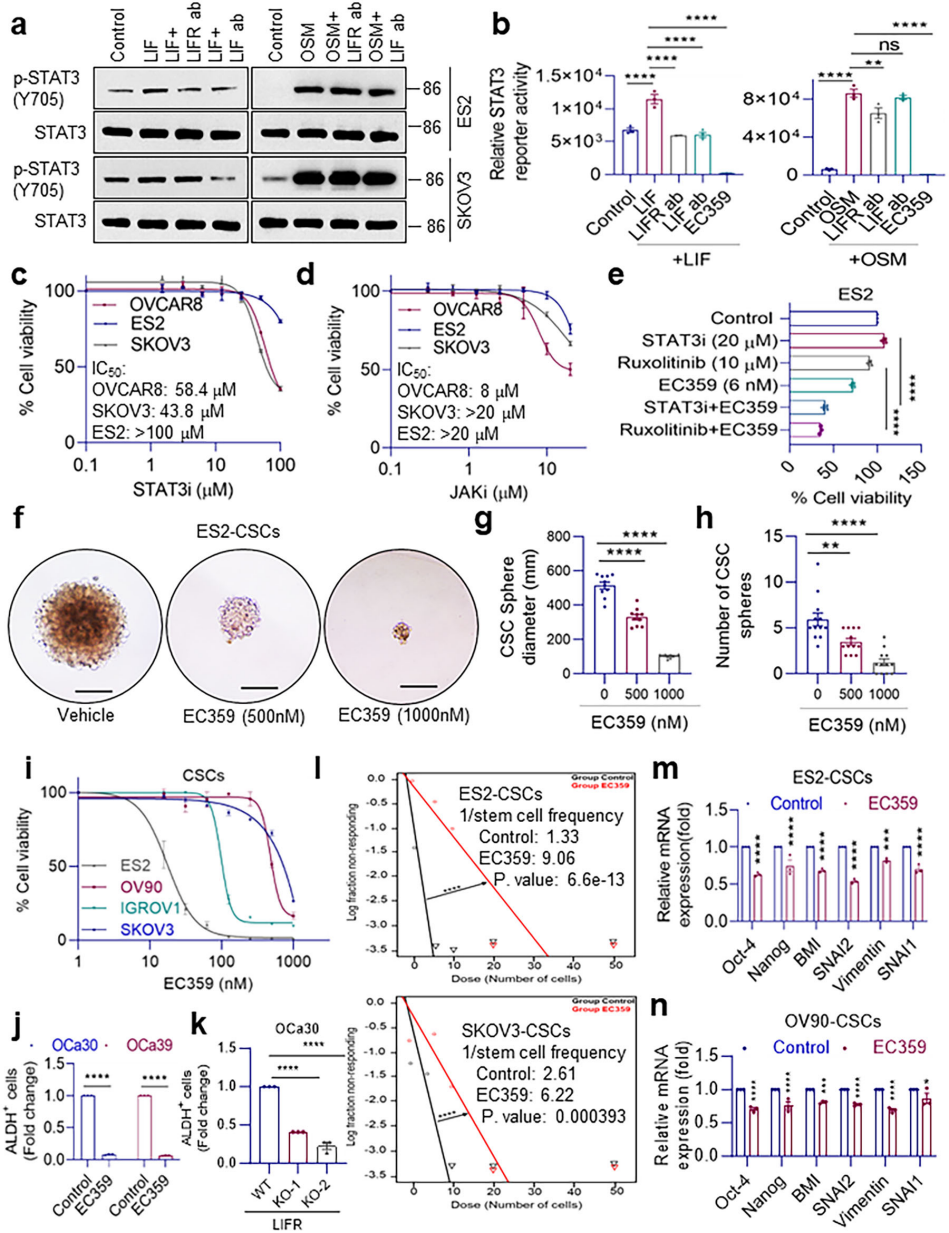
EC359 is more effective in blocking LIFR signaling compared to anti LIF antibody and STAT3/JAK inhibitors and suppresses stemness properties of OCa cells. **a** Changes in the phospho-STAT3 levels in ES2 and SKOV3 cells when treated with recombinant human LIF (100 ng/ml) or recombinant human Oncostatin M (OSM) (10 ng/ml) in the presence or absence of anti-LIFR (10 µg/ml) or anti-LIF (100 ng/ml) antibodies for 10 h. Western blots in each panel are derived from the same experiment and processed in parallel. **b** Reporter assay of SKOV3 cells stably expressing STAT3-Luciferase treated with human recombinant LIF (100 ng/ml), or recombinant human OSM (10 ng/ml) in the presence or absence of anti-LIFR (10 µg/ml) or anti-LIF (100 ng/ml) antibodies or EC359 (100 nM) for 20 h. **c** Cell viability analysis of OVCAR8, ES2, and SKOV3 cells treated with a serial dilution of STAT3 inhibitor NSC-74859 using MTT assay. IC_50_ values for each cell line are noted on the graph. **d** MTT assay measuring the effect of JAK inhibitor Ruxolitinib on cell viability of OVCAR8, ES2, and SKOV3 cells. IC_50_ values for each cell line for Ruxolitinib are stated on the graph. **e** Comparison of the effects of STAT3i NSC-74859 and JAKi Ruxolitinib alone or in combination with EC359 on cell viability of ES2 cells using MTT assay. **f** Representative sphere images of ES2 CSCs treated with vehicle or EC359 for two weeks performed by sphere formation assay. Scale bar represents 200 µm. **g** Bar graphs presenting quantification of spheres dimension, and (**h**) number of spheres formed. **i** SKOV3, OV90, ES2, and IGROV1 CSCs (ALDH^+^) were treated with EC359, and the cell viability was quantitated using CellTiter-Glo® assay. **j** Primary OCa cells (OCa30 and OCa39) were treated with EC359 (500 nM) for 24 h and the percentages of ALDH^+^ cells were determined using flow cytometry. **k** WT and LIFR-KO OCa30 cells were stained for ALDH^+^ cells and the percentages of ALDH^+^ cells were determined using flow cytometry. **l** Graphs displaying extreme limiting dilution assays. Stem cell frequency estimates (with confidence intervals) of ES2-CSCs and SKOV3-CSCs treated with vehicle or EC359 (1 µM) for 2 weeks generated through ELDA. Relative expression of stemness and EMT-related genes in ES2-CSCs (**m**) and OV90 CSCs (**n**) treated with EC359 (1 µM) for 24 h compared to the control groups. Data presented as mean ± S.E.M., *n* = 3 biologically independent samples. Significance was determined by two-way ANOVA followed by Uncorrected Fisher’s LSD. Numerical source data for (**b**–**e**, **g**–**n**) and uncropped Western blot images for (**a**) are provided. ns not significant; *p < 0.05, **p < 0.01, ***p < 0.001, ****p < 0.0001.

LIFR is activated by multiple ligands such as LIF, OSM that are widely expressed in OCa^21,22^. Western blot analyses and STAT3 reporter assays showed that LIF ab (10 µg/ml) is effective in reducing LIF (100 ng/ml)/LIFR mediated STAT3 signaling; however, LIF ab or LIFR ab treatment had limited effect on OSM/LIFR mediated STAT3 signaling (Fig. 3a, b). Uniquely, EC359 (100 nM) reduced activation of STAT3-luciferase reporter induced by LIF or OSM and that reduction was significantly more than that of LIF ab or LIFR ab (Fig. 3b). Since STAT3 is one of the major pathways activated by LIFR, we compared the efficacy of STAT3 and JAK inhibitors with EC359 using MTT assays. Results showed that STAT3i has an IC_50_ of 50-100 µM and JAKi has an IC_50_ of 8-50 µM (Fig. 3c, d) compared to EC359 with IC_50_ as low as 5-50 nM (Fig. 2a). Moreover, combination of EC359 (6 nM) with STAT3i or JAKi was able to dramatically reduce cell viability of ES2 cells compared to either one alone indicating potential of EC359 in enhancing the efficacy of STAT3i or JAKi (Fig. 3e). These pieces of evidence support the value of targeting STAT3 and JAK pathways in OCa; nevertheless, EC359 has a significant translational advantage over other inhibitors due to its low nM activity and low toxicity.

### EC359 treatment diminishes stemness of OCa

Aldehyde dehydrogenase (ALDH^+^) cancer stem cells (CSCs) isolated from ES2 cells were treated with EC359 and the sphere formation ability, sphere diameter and cell viability was determined. Results revealed that EC359 treatment significantly decreased CSCs diameter, number of spheres and reduced cell viability (Fig. 3f-i). We confirmed the effect of EC359 on cell viability using three additional OCa cells including OV90, SKOV3, IGROV1 (Fig. 3i). We then examined the effect of EC359 treatment on the status of the ALDH^+^ cells. Primary OCa cells (OCa30 and OCa39) were treated with EC359 and the proportion of ALDH^+^ cells were determined using FACS analyses after 24 h of treatment. Results showed a significant reduction in the percentage of ALDH^+^ cells in EC359 treated group compared to the vehicle treated control group (Fig. 3j). This is similar to the effect of LIFR-KO on the proportion of CSCs (Fig. 3k). To test the effect of EC359 on stem cell frequency of CSCs, we conducted extreme limiting dilution assays (ELDA). Results showed reduction in stem cell frequency in EC359 treated groups compared to the vehicle treated group (Fig. 3l). In addition, since LIF signaling regulates stemness transcription factors^23^, RT-qPCR analyses of EC359 treated CSCs isolated from ES2 and OV90 cells showed decreased expression of a number of genes implicated in stemness (Fig. 3m, n). This data indicates that LIFR inhibitor EC359 suppresses stemness properties of CSCs in OCa.

### EC359 promotes ferroptosis in OCa cells

**Fig. 4:**
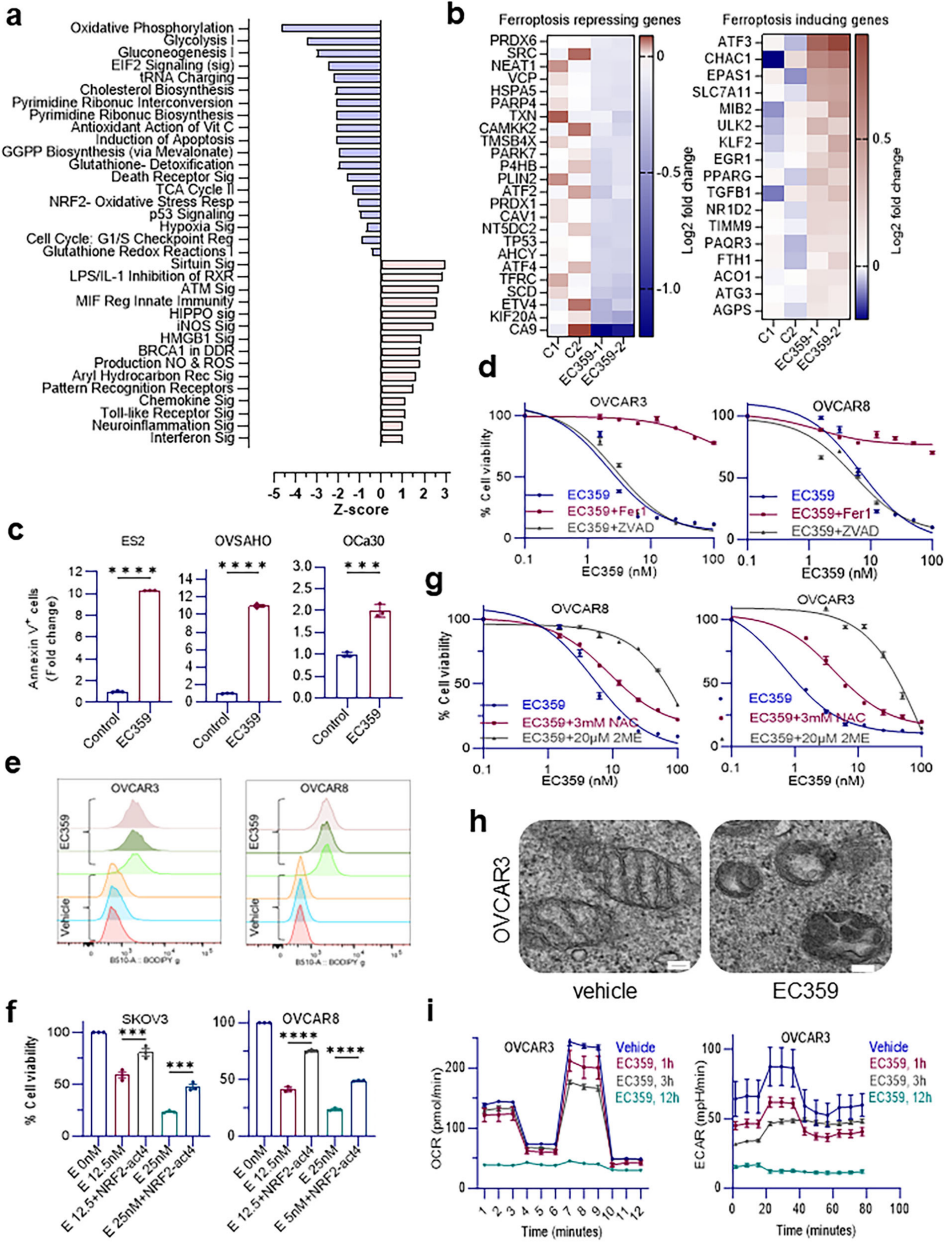
EC359 induces cell death through ferroptosis. **a** IPA of ES2 cells treated with EC359 (10 nM) for 12 h connotates downregulation of oxidative phosphorylation, glutathione-mediated detoxification, and NRF2-mediated oxidative stress response. **b** Heatmap visualization of data from RNA-Seq of ES2 cells treated with EC359 representing upregulation of ferroptosis inducing genes and downregulation of ferroptosis-repressing genes. *n* = 2 biologically independent samples. **c** Annexin V flow cytometry analysis of ES2, OVSAHO, and OCa30 cells treated with 100 nM of EC359 for 24 h shows induction of cell death by disruption of cell membrane integrity compared to the control groups. Data presented as mean ± S.E.M., *n* = 3 biologically independent samples. Significance was determined by Two-tailed Unpaired *t* test. **d** Cell viability assay of OVCAR3 and OVCAR8 cells treated with EC359 alone or in combination with ferroptosis and apoptosis inhibitors, Ferrostatin-1 (2 µM) and Z-VAD-FMK (10 µM) respectively for four days using MTT reagent. Data presented as mean ± S.E.M., *n* = 3 biologically independent samples. **e** Flow cytometric detection of lipid peroxidation using BODIPY™ 581/591 C11 probe in OVCAR3 and OVCAR8 cells treated with vehicle or EC359 (20 nM) for 12 h showing an increase in the green fluorescent (∼510 nm) intensity in EC359 treated cells. *n* = 3 biologically independent samples. **f** Cell viability measurement of SKOV3 and OCVAR3 cell lines treated with Vehicle (E 0 nM), or EC359 (E 12.5 nM, E 25 nM) in combination with NRF2-activator-4 (2 µM). **g** Cell viability assay of OVCAR3 and OVCAR8 cells subjected to EC359 treatment alone or in combination with NAC (3 mM) or 2 ME (20 µM) over a 4-day period using MTT reagent. Data presented as mean ± S.E.M., *n* = 3 biologically independent samples. Significance was determined by two-way ANOVA followed by Uncorrected Fisher’s LSD. **h** Transmission Electron Microscopy images of OVCAR3 cells treated with vehicle or 100 nM EC359 for 9 h. Scale bar = 100 nm. **i** Oxygen consumption rate (OCR) and extracellular acidification rate (ECAR) of OVCAR3 cells treated with vehicle or 100 nM of EC359 for 1, 3, or 12 h. Data presented as mean ± S.E.M., *n* = 3 biologically independent samples. Numerical source data for Fig. (**a**–**g**, **i**) and uncropped images for (**h**) are provided. ns not significant; ***p < 0.001, ****p < 0.0001.

To understand the molecular mechanism by which LIFR inhibitor EC359 diminishes cell viability of OCa cells, we profiled gene expression changes upon EC359 treatment using global RNA-seq analysis. EC359 treatment differentially regulated 1992 genes (p < 0.01). Ingenuity Pathway Analysis (IPA) of EC359 down regulated genes identified several pathways including oxidative phosphorylation, Glutathione signaling, JNK signaling, NRF2 signaling, ovarian cancer signaling, and hypoxia signaling (Fig. 4a). EC359 down regulated pathways such as NRF2 signaling, oxidative phosphorylation, and glutathione signaling play important roles as a defense mechanism against ferroptosis, a type of cell death that is characterized by lipid peroxidation and intracellular iron buildup. Additionally, examination of RNA-seq data confirmed upregulation of ferroptosis inducing genes and downregulation of ferroptosis repressing genes (Fig. 4b) demonstrating the failure of the ferroptosis defense mechanisms that leads to ferroptosis and cell death. We initially confirmed that EC359 promoted cell death of OCa cells using Annexin V assay which shows loss of cell membrane integrity as the early stage of cell death (Fig. 4c). Treatment of OCa cells with Ferrostatin-1 (Fer-1), a lipophilic radical scavenger and inhibitor of ferroptosis, but not with pan-caspase inhibitor z-VAD-FMK abolished EC359 mediated cell death confirming RNA sequencing results (Fig. 4d).

Lipid peroxidation is a hallmark of ferroptosis. Flow cytometry assessment of lipid peroxidation in cells through oxidation of BODIPY™ 581/591 C11 reagent also demonstrated ferroptosis (Fig. 4e)^24^. Since STAT3 is one of the downstream targets of LIFR, and positively modulates levels of SLC7A11 and GPX4^24^ we examined the expression levels of SLC7A11 and GPX4 in EC359 treated OCa cells. Western blotting analyses of EC359 treated OCa cells confirmed down regulation of SLC7A11 and GPX4. Similarly, LIFR-KO cells exhibited decreased levels of SLC7A11 and GPX4 (Supplementary Fig. 2a). A time course analysis on LIFR downstream signaling molecule STAT3 and ferroptosis markers showed that STAT3 downregulation occurs as early as 4 h after EC359 treatment, whereas substantial downregulation of ferroptosis markers (SLC7A11, GPX4) occurs after 6 h. These findings indicate that the suppression of LIFR downstream signaling occurs prior to the commencement of ferroptosis (Supplementary Fig. 2c).

To further understand the mechanism, we examined whether activation of STAT3, a down-stream effector of LIFR signaling rescues cells from EC359-mediated cell death. We generated ES2 model cells stably expressing constitutively active STAT3 (CA-Stat3-t2A-mCherry)^25^. The results showed that cells expressing CA-STAT3 reduced EC359-mediated cell death compared to parental vector-expressing cells (Supplementary Fig. 2c). Since our RNA-seq results showed that EC359 treatment downregulates NRF2 signaling, we examined whether activation of NRF2 rescues cells from EC359 mediated reduction in cell viability. Results confirmed that activation of NRF2 promotes resistance of OVCAR8 and SKOV3 cells to EC359-mediated decrease in cell viability (Fig. 4f). Since EC359 reduces the level of SLC7A11, we tested if bypassing system Xc^−^ by supplying cysteine for GSH synthesis can rescue cells from death. For these assays, we utilized cysteine prodrug *N*-acetyl cysteine (NAC), a commonly used antioxidant that is cell permeable, or the thiol reagent 2-mercaptoethanol (2ME) that can reduce cystine to cysteine extracellularly which is taken up by cells via neutral amino acid transporters^26^. Results using both OVCAR8 and OVCAR3 cells showed that NAC or 2ME treatment promotes resistance to EC359-mediated cytotoxicity (Fig. 4g). Collectively, these results suggest that LIF/LIFR autocrine signaling promotes cell survival by suppressing ferroptosis and EC359 mediated inhibition of LIFR downstream signaling such as STAT3, downregulates glutathione mediated defense mechanism allowing unresolved lipid peroxidation to cause catastrophic damage to the cells and eventually death.

### EC359-treatment decreases the glycolysis/mitochondrial respiration and calcium signaling in ovarian cancer cells in a time-dependent manner

To further understand the mechanism of EC359-mediated cytotoxicity, we used transmission electron microscopy (TEM) to examine the sub-cellular structures of OCa cells that had been treated with EC359. Results demonstrated that there was a loss of cristae and altered mitochondrial morphology (Fig. 4h, Supplementary Fig. 3) which are characteristics of ferroptosis. To further confirm whether EC359 decreases the metabolic activity of OCa cells, we assessed mitochondrial function and glycolytic rate by measuring oxygen consumption rate (OCR) and extracellular acidification rate (ECAR), respectively in OVCAR3 cells using Seahorse assay. Results revealed a notable and time-dependent reduction in basal ECAR, indicative of decreased lactate efflux when OVCAR3 cells were exposed to 100 nM of EC359 for 1, 3, and 12 h (Fig. 4i). Glycolysis has long been considered the major metabolic process for energy production and anabolic growth in cancer cells. A decrease in ECAR is associated with a decrease in cell activity and proliferation. As induced by EC359 treatment, a time-dependent decrease in ECAR is also correlated with a decrease in OCR maximal respiratory, spare respiratory capacity, and proton leak (Supplementary Fig. 2d). Mitochondrial spare respiratory capacity represents the possibility of the cell maintaining an increased ATP level under stress conditions. In addition, only after 12 h of treatment OVCAR3 cells showed a significant decrease in basal respiration, ATP production, coupling efficiency (%), and non-mitochondrial oxygen consumption (Supplementary Fig. 2d). It is known that besides energy-yielding function, mitochondria also provide building blocks for tumor anabolism, control redox, calcium homeostasis, participate in transcriptional regulation, and govern cell proliferation. Notably, OVCAR3 cells after 12 h of treatment with EC359 showed a decrease in OCR basal respiration, ATP production, and spare respiratory capacity, almost to 0, which could indicate pre-death conditions. Altogether, these data indicate that EC359 could decrease glycolysis and mitochondrial function of OVCAR3 cells in a time-dependent manner and could serve as an important drug for treating OCa.

As calcium signaling is critical for endoplasmic reticulum (ER), and mitochondrial function, as well as cell proliferation, we proceeded to evaluate calcium signaling in these cells. We introduced thapsigargin (Tg, 2 μM), a sarcoendoplasmic reticulum Ca^2+^ transport ATPase (SERCA) blocker that depletes intracellular ER Ca^2+^ stores and activates Ca^2+^ entry. Importantly, thapsigargin (Tg, first peak) induced an increase in [Ca^2+^]_i_ in untreated-control cells, which was significantly attenuated in OVCAR3 cells that were treated with EC359 (100 nM for 3 h) (Supplementary Fig. 2e). Subsequently, the addition of 1 mM external Ca^2+^, which initiates Ca^2+^ entry, was also significantly decreased in EC359-treated cells (Supplementary Fig. 2e). Together these results suggest that EC359 treatment leads to a decrease in ER and cytosolic calcium levels thereby compromising mitochondrial function and promoting cell death of cancer cells. Collectively, these results suggest that EC359 treatment contributed to induction of cell death of OCa cells by altering glycolysis/mitochondrial respiration and calcium signaling.

### Maximum tolerated dose (MTD) and dose range finding studies for EC359

To study MTD, we assessed the pharmacokinetics and toxicological profile of EC359 in SD rats following intraperitoneal and oral administration. Single oral doses of EC359 ranging from 10 to 30 mg/kg revealed dose proportional increase in exposure (C_max_ and AUC_0−t_). However, doses ranging from 30 to 100 mg/kg showed less than dose-proportional increase in exposure (Supplementary Fig. 4a, b). No accumulation was noted following repeated dose administration at doses of 10, 30, and 100 mg/kg. During single dose phase, all animals treated with 2.5 and 5 mg/kg/dose were found to be free from all visible clinical signs. Animals treated at 15 mg/kg were dull following 1 h post treatment and persisted for around 2 h. Dullness was observed in animals treated at 50 and 100 mg/kg starting 1 h post treatment and continued till the end of the day. All animals recovered from the observed clinical signs around 24 h after treatment. During the repeated dose phase, no clinical signs were observed in animals treated with 10 mg/kg/dose throughout the treatment period. Raised hair and dullness were observed on Day 1 post dose in male animals treated at 30 mg/kg/dose until sacrifice. Administration of test item of EC359 at 100 mg/kg body weight caused dullness, raising of hair (piloerection), diarrhea, lethargy, ptosis, prostration, abnormal gait, epistaxis and tremors from day 1/2 onwards and persisted continuously until sacrifice on day 6. During single dose phase, normal body weight gain was observed following treatment at around 48 h in animals treated up to 0, 2.5, 5, 15 and 50 mg/kg as compared to respective pre-exposure weights. The MTD recorded in single dose investigation phase is 100 mg/kg. There was no body weight gain in female animals treated at 100 mg/kg. However, animals treated at 300 mg/kg/dose exhibited a marginal decrease (<10%) in body weight following 48 h post dose. No significant decreases in body weight and food consumption were observed in animals treated at dose levels of 10 mg/kg when compared to the control group in repeated dose phase. No treatment related changes in the hematological, coagulation and clinical chemistry parameters were observed in any of the treated groups up to 30 mg/kg as compared to control group. No gross abnormalities were observed in any of the treated groups up to 30 mg/kg and control animals. Oral administration of EC359 for 7 consecutive days produced excessive toxicity along with significant decreases in body weight and feed consumption at 100 mg/kg resulting in early termination of the rats on Day 6. At 30 mg/kg, dullness and raised hair were observed. The dose of 10 mg/kg did not reveal any abnormalities attributable to treatment. Based on findings, the MTD of EC359 following repeated oral administration for 7 consecutive days was 30 mg/kg in SD rats under the tested conditions. These findings suggest a dose-dependent safety profile of EC359, with 30 mg/kg being the MTD in this study.

### EC359 reduces the growth of OCa xenograft tumors in vivo and ex vivo

**Fig. 5:**
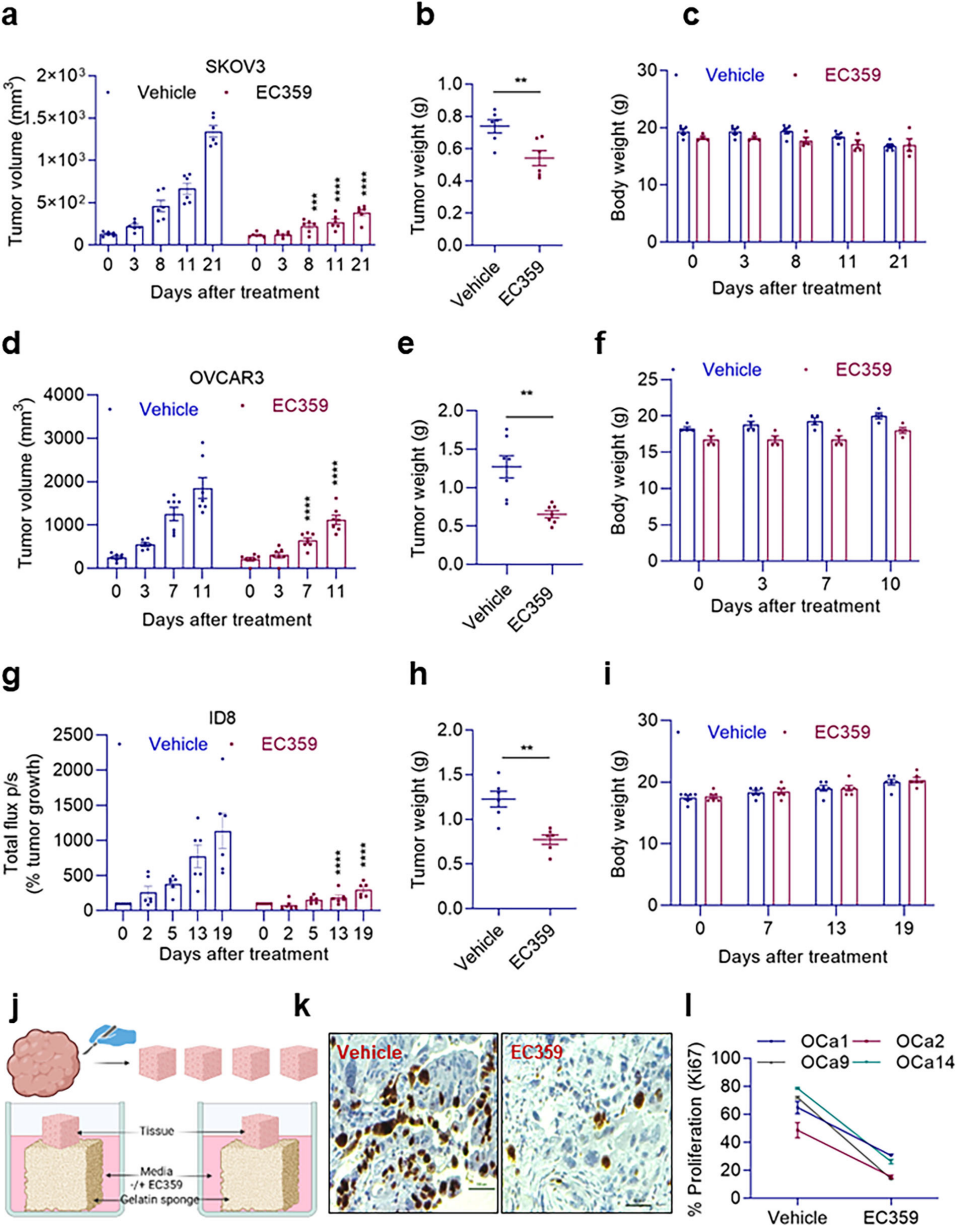
EC359 reduces tumor progression in cell-derived xenograft mouse models. Bar graphs presenting tumor volume of SKOV3 (**a**) and OVCAR3 (**d**) xenografts that were treated with vehicle or EC359 (5 mg/kg/day/s.c./5 days/week and 10 mg/kg/day/i.p./3 days/week respectively). Tumor weights of SKOV3 and OVCAR3 xenografts presented in (**b** and **e**) and changes in body weight for SKOV3 and OVCAR3 bearing SCID mice were shown in (**c** and **f**), respectively. Data presented as mean ± SEM, *n* = 6 tumors per group for (**a**), and *n* = 7 tumors per group for (**d**). Significance was determined by two-way ANOVA followed by Uncorrected Fisher’s LSD. Murine luciferase labeled ID8 OCa xenografts injected i.p., in syngeneic mice was treated with vehicle or EC359 (10 mg/kg/day/i.p). Tumor progression was monitored using Xenogen imaging system (**g**). **h** and **i** show tumor weight and body weight measurements of vehicle and EC359 treated mice. Data presented as mean ± SEM, *n* = 6 mice per group. Significance was determined by two-way ANOVA followed by Uncorrected Fisher’s LSD. **j** Schematic representation of ex vivo explant assay created with BioRender.com. **k** IHC image of tumor tissues treated with vehicle or EC359 and subjected to Ki67 immunostaining. **l** A quantification of the changes in the percentage of Ki67 positive cells in primary ovarian tumor tissue explants treated with vehicle or EC359 for 72 h. Data presented as mean ± SEM, *n* = 3 tumors per group. Numerical source data for (**a**–**i**), and (**l**) are provided. **p < 0.01, ***p < 0.001, ****p < 0.0001.

To evaluate the effectiveness of EC359 in inhibiting OCa tumor growth both in vivo and ex vivo we used various preclinical models. We established SKOV3 xenograft tumors in female SCID mice to assess the effectiveness of EC359 on OCa tumor growth in vivo using subcutaneous implantation of cells. Mice were randomly assigned to receive either vehicle or EC359. Compared to the vehicle treated group, EC359 therapy slowed the progression of the tumors and decreased tumor weights with no change in body weights (Fig. 5a–c). When compared to tumors treated with vehicle, EC359-treated tumors presented fewer proliferating cells (Ki67 positive cells) (Supplementary Fig. 4c). We validated these results using the OVCAR3 xenograft model as well. Results displayed that EC359 therapy also slowed the progression of the OVCAR3 tumors and decreased tumor weights with no change in body weights (Fig. 5d–f). We then tested the utility of EC359 using orthotopic murine ID8 OCa model cells labeled with luciferase reporter using C57BL/6 mice. Tumors were established by injecting ID8 cells into intraperitoneal cavity and tumor growth was monitored using Xenogen in vivo imaging system. Results exhibited that EC359 treatment significantly reduced the tumor volume and weight with no changes in body weight compared to vehicle treated group (Fig. 5g–i). Using an ex vivo culture model of primary OCa tumors, we further examined the effectiveness of EC359 on tumor tissue growth. Briefly, primary tumors were sliced into small pieces, placed on gelatin sponge soaked in the culture medium, and cultured for 72 h in the presence of EC359 or vehicle (Fig. 5j). Compared to vehicle-treated tumor tissues, proliferation of OCa explants treated with EC359 was markedly reduced (Fig. 5k, l). Similarly, in organoid assays using primary tumor tissues, EC359 treatment considerably reduced the cell viability of organoids compared to the vehicle (Supplementary Fig. 4d). Collectively, these results suggest that EC359 has a potent antitumor activity on OCa preclinical models in vivo and primary tissues ex vivo.

### EC359 treatment induces lymphocyte alterations in the tumor microenvironment

**Fig. 6:**
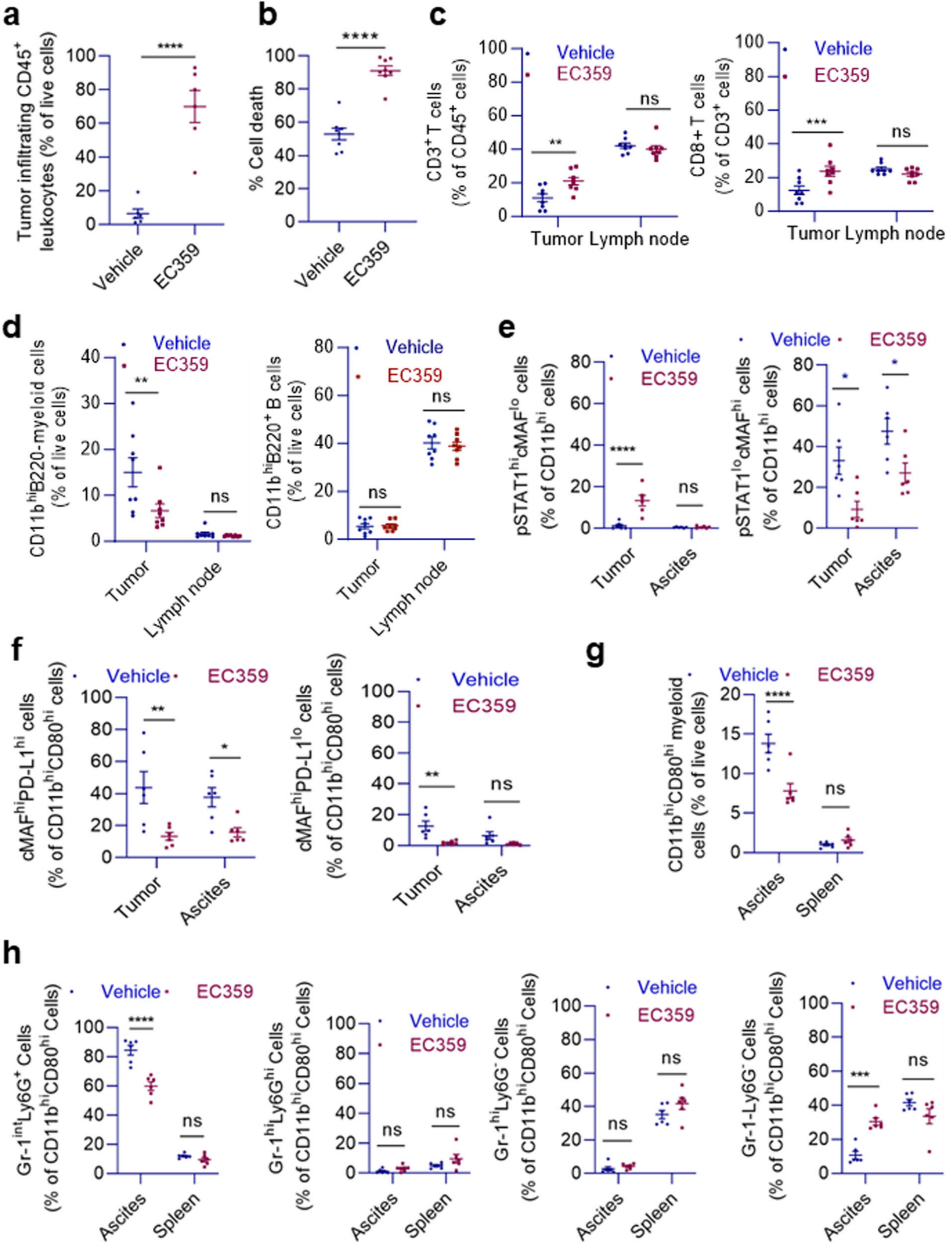
EC359 treatment induces lymphocyte alterations in the tumor microenvironment in a syngeneic OCa mouse model. **a** Flow cytometry analysis of (CD45^+^) leukocyte infiltration into ID8 tumors in vehicle-treated C57BL/6 mice or residual tumors in EC359-treated mice without prior leukocyte enrichment. **b** Flow cytometry analysis of the proportion of dead (FVD^+^) cells among GFP^+^ cells in OCa tumors, as derived from ID8 cells stably expressing GFP through lentiviral transduction, in mice treated with vehicle or EC359. **c** Flow cytometry analysis of the proportion of CD3^+^ T cells within (CD45^+^) leukocytes that infiltrated into (residual) ID8 tumor-infiltrating leukocytes (after pre-enrichment with Ficoll-Paque) or were present in draining mesenteric lymph node, as indicated, as well as the proportion of CD8^+^ T cells within CD3^+^ cells (right panels) in mice treated with vehicle or EC359. **d** Flow cytometry analysis of the proportion of CD11b^hi^B220^–^ myeloid cells and CD11b^–^B220^+^ B cells in (CD45^+^) leukocytes in ID8 tumors or lymph node, as in (**c**). **e** Flow cytometry analysis of the proportion of CD11b^+^ cells that expressed phosphorylated STAT1 (pSTAT1), a hallmark transcription factor of pro-inflammatory M1 macrophages, or cMAF, a hallmark transcription factor of anti-inflammatory M2 macrophages, in (residual) ID8 tumors (after pre-enrichment with Ficoll-Paque) or ascites in mice treated with vehicle or EC359. **f** Flow cytometry analysis of the expression of cMAF and the PD-L1 immune checkpoint in CD11b^+^CD80^hi^ myeloid cells in ID8 tumors or ascites, as in (**e**). **g**, **h** Flow cytometry analysis of the proportion of CD11b^+^CD80^hi^ myeloid cells in ascites and the spleen as well as the proportion of the four subsets based on the expression of Gr1 and Ly6G, as indicated, and their respective PD-L1 expression levels (bottom left) in ID8 cell-engrafted mice treated with vehicle or EC359. The Gr-1^int^Ly6G^+^ subset displayed the highest proportion and PD-L1 expression in ascites. Data presented as mean ± SEM, *n* = 6 mice per group. Significance was determined by Unpaired *t* test for (**a**, **b**) and two-way ANOVA followed by Šídák’s multiple comparisons test for (**c**–**g** ns not significant; Numerical source data for (**a**–**g**) are provided. *p < 0.05, **p < 0.01, ***p < 0.001, ****p < 0.0001.

To assess the effect of EC359 on the pattern of immune cells infiltrating tumors, we utilized a syngeneic mouse model using ID8 cell line and C57BL/6 immunocompetent mice. In association with the OCa tumor-killing effect of EC359, residual ID8 tumors in EC359-treated mice had massive infiltration of CD45^+^ leukocytes, which accounted for 75% of all live cells, as compared to only 5% in tumors in vehicle-treated mice (Fig. 6a, Supplementary Fig. 5a). This reflected over 90% of OCa cell death triggered by the EC359 treatment (Fig. 6b) that was concomitant with increased infiltration by CD3^+^ T cells and, CD8^+^ cytotoxic T lymphocytes (CTLs), as compared to leukocytes isolated from tumors in vehicle-treated mice (Fig. 6c, Supplementary Fig. 5b). Among lymphocytes that can modulate T cell functions in the tumor microenvironment, CD11b^+^ myeloid cells, but not B cells, were hampered in their infiltration by EC359 (Fig. 6d, Supplementary Fig. 5c), which, however, did not affect the proportions of T cells, B cells or myeloid cells in draining lymph nodes (Fig. 6c, d). The reduction of CD11b^+^ myeloid cells upon EC359 treatment was mainly due to the decrease in those expressing cMAF, the hallmark transcription factor of M2 macrophages, which suppress the antitumor immunity (Fig. 6e, Supplementary Fig. 5c). By contrast, CD11b^+^ cells expressing STAT1 phosphorylated at Tyr701 (pSTAT1), which is the hallmark transcription factor of inflammatory M1 macrophages, were increased (Fig. 6e, Supplementary Fig. 5d), likely also contributing to the tumor-killing effects. Among cMAF^hi^ CD11b^+^CD80^hi^ myeloid cells (macrophages), those expressing the PD-L1 immune checkpoint (and, therefore, could potently inhibit CTL activation) were reduced in EC359-treated mice (Fig. 6f, Supplementary Fig. 5e), also consistent with the reverse of immune suppression. In addition, cMAF^hi^ and cMAF^hi^PD-L1^hi^ macrophages were significantly reduced in ascites (Fig. 6e, f), the external environment of ID8 tumors that may also affect immune cell functions – pSTAT1^hi^CD11b^+^ cells were much fewer in ascites. Indeed, PD-L1^hi^ myeloid cells, particularly Gr-1^int^Ly6G^+^ cells, were decreased in ascites in mice treated with EC359, which also increased Gr-1^–^Ly6G^–^ cells and abrogated their PD-L1 expression (Fig. 6g, h, Supplementary Fig. 5f), emphasizing a role of EC359 in remodeling the external environment of OCa tumors. These findings suggest that EC359 treatment showed prominent effects on the immune cell infiltration pattern within the tumors, promoting the infiltration of anti-tumor immune cells while inhibiting immunosuppressive myeloid cell populations.

### EC359 reduced in vivo tumor progression in OCa PDX models

**Fig. 7:**
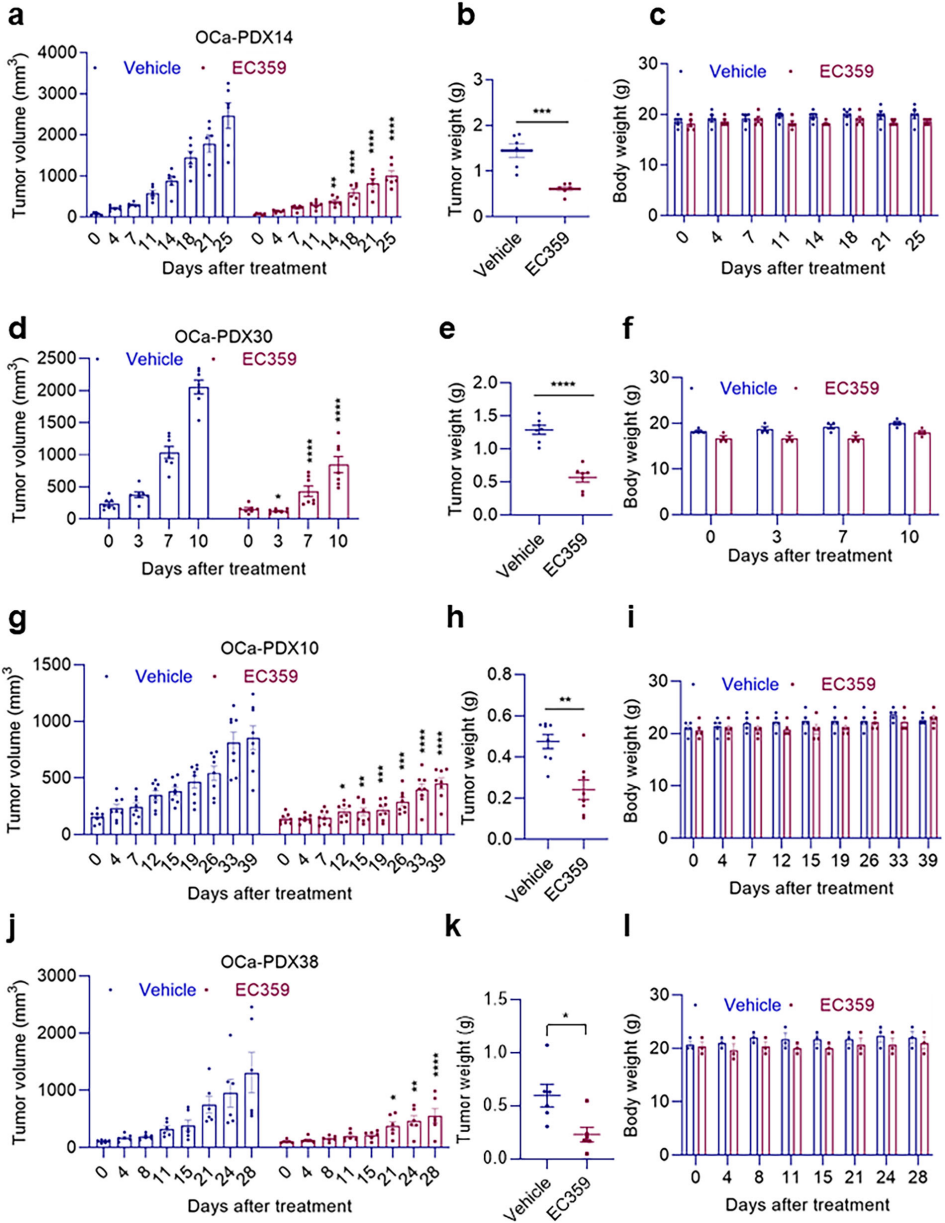
EC359 hinders tumor growth in patient-derived xenograft models. **a**, **d**, **g**, and **j** show tumor volume measurements in four different experiments of mice bearing four different PDX tumors (OCa-PDX14, OCa-PDX30, OCa-PDX10, and OCa-PDX38) treated with vehicle or EC359 (OCa-PDX14: 7.5 mg/kg/s.c./once/week; OCa-PDX30: 10 mg/kg/oral/3 days/week; OCa-PDX10: 2.5 mg/kg/i.p./every day; OCa-PDX38: 5 mg/kg/i.p./3 days/week). **b,** **e,** **h**, and **k** display changes in tumor weight at the end of the in vivo studies. Changes in the body weight of the four different in vivo PDX experiments are presented in (**c,** **f,** **i** and **l**). Data presented as mean ± SEM, *n* = 6 tumors per group for (**a**), *n* = 7 tumors per group for (**d**), *n* = 8 tumors per group for (**g**) and *n* = 6 tumors per group for (**j**). Significance was determined by two-way ANOVA followed by Uncorrected Fisher’s LSD. Numerical source data for (**a**–**l**) are provided. *p < 0.05, **p < 0.01, ***p < 0.001, ****p < 0.0001.

To test the effect of EC359 on a model that recapitulates patient responses to treatment we used patient-derived xenografts (PDXs) that maintain tumor heterogeneity. In this respect, we examined the effectiveness of EC359 in treating OCa using a cohort of PDXs that represented the endometrioid (PDX14), serous (PDX30), neuroendocrine (PDX10), and chemotherapy-resistant serous (PDX38) subtypes of OCa. As shown in Fig. 7a, d, g, and j, therapy with EC359 considerably slowed tumor growth in all four PDX models tested, decreased tumor weights (Fig. 7b, e, h, and k) and had no effect on body weights (Fig. 7c, f, i, and l). When compared to tumors treated with vehicle, IHC imaging revealed that EC359-treated tumors had fewer proliferating cells (Ki67 positive cells) (Supplementary Fig. 6a, b). These findings imply that EC359 has antitumor efficacy in OCa PDX tumors.

## Discussion

OCa is the most lethal of all gynecologic malignancies due to its insidious development and progression, often leading to its diagnosis at an advanced stage. The high probability of recurrence following the initial chemotherapy renders present treatment options ineffective, resulting in severe morbidity and short survival rates. To increase both overall survival and progression-free survival of OCa patients, there is a pressing clinical need to create new targeted agents. In this investigation, we identified the LIF/LIFR axis as a therapeutic target to treat OCa. Our research underscores the importance of the LIF/LIFR autocrine loop for OCa cell survival, stemness, tumor immunity, and progression.

LIF/LIFR axis has been connected to numerous hallmarks of cancer, including proliferation, avoiding immune system damage, chemoresistance, and overall patient survival^12,27,28^. An autocrine or paracrine loop of LIF/LIFR has been shown in different types of cancer. For example, LIF acts in a paracrine manner on pancreatic cancer cells to support tumor progression^29^. Paracrine/autocrine LIF/gp130/STAT3 pathways is involved in stemness and invasion of pancreatic cancer^30^. In head and neck as well as lung carcinomas, LIF is produced by tumor cells and induces fibroblast activation in a paracrine manner to promote an invasive TME^15^. LIF overexpression is shown to activate autocrine signaling and tumorigenesis of breast cancer cells^31^. OCa-associated mesenchymal stem cells are shown to secrete IL6 and LIF to induce tumor cell stemness^13^. These findings indicate that some cancers rely on autocrine or paracrine LIF signaling to maintain their growth and development. Our findings utilizing various LIFR-KO and OCa models showed that LIF is produced by OCa cells and is crucial for OCa proliferation and tumor development via LIFR in an autocrine way.

LIF blockade by neutralizing antibodies^29^ or engineered ligand trap^32^ demonstrated utility of blocking LIF/LIFR axis for cancer treatment^33^. We recently developed and characterized LIFR inhibitor, EC359, that selectively binds to the LIFR and blocks binding of its ligands^34^. Functionality and specificity of EC359 was validated using triple-negative breast cancer^34,35^ and endometrial cancer^36,37^. Other published studies also demonstrated the efficacy of EC359 on suppressing pancreatic cancer^38,39^ and renal cancer cells^40^. MTD and dose range finding studies established 30 mg/kg dose of EC359 as a well-tolerated dose with no observable toxicity. In this study utilizing multiple OCa cells and tumor tissues, we provided evidence that EC359 potently reduces OCa cell viability and progression in vivo.

LIF/LIFR axis is implicated in the maintenance of stem cells^23,41^, is linked to cancer stem cell enrichment in tumors^13,28,42^, epithelial-mesenchymal transition^39,43–45^ and chemoresistance^46–49^. LIF/LIFR pathway was recently identified as a potential inducer of serous OCa upon chronic Chlamydia infection by increasing stemness in fallopian tube organoids^50^. Here, we showed that genetic ablation of LIFR or its pharmacological inhibition reduced the abundance of CSCs in OCa as well as stemness properties. Additionally, EC359 is efficient in inhibiting the progression of chemotherapy resistant OCa. The role of LIF/LIFR autocrine signaling in tumor recurrence is substantial since following chemotherapy, residual CSCs might repopulate tumor once again utilizing positive autocrine loop without the need of paracrine factors from the host tissue. Our findings imply that EC359 performs well as a small molecule inhibitor of LIF/LIFR signaling. These results suggest a possibility of inhibiting the LIF/LIFR signaling system concurrent with chemotherapy and/or post-chemo as maintenance therapy to improve the efficacy of current standard of care and patient’s outcome.

Generation of reactive oxygen species (ROS) is a byproduct of cellular metabolism and normal cellular activities. However, cells evolutionary acquired different antioxidant defense systems to counteract harmful effects of ROS on cell survival. ROS (e.g., H_2_O_2_) can engage in the Fenton catalytic reaction with iron ions to generate highly cytotoxic hydroxyl radicals (•OH), which then attack polyunsaturated fatty acids in the cellular lipid bilayers (e.g., cell membrane). This process in the absence or malfunction of antioxidant defense system leads to ferroptotic cell death^51–53^. One of the key ferroptosis defense mechanisms is the glutathione peroxidase 4 (GPX4)–reduced glutathione (GSH) system^53^. Our results showed that EC359 downregulates SLC7A11 and GPX4 which suppresses GSH mediated defense system leading to lipid peroxidation and cell death. Rescue experiments with the NRF2 activator, CA-STAT3 overexpression or bypassing SLC7A11 demonstrated that EC359 suppresses GSH defense system partly by inhibition of STAT3 signaling, which subsequently downregulates NRF2 expression and consequent down regulation of GPX4 and SLC7A11. These findings match with the recent reports that EC359 induces ferroptosis in renal cancer cells by GPX4 inactivation and GSH depletion^40^. Furthermore, RNA-seq analysis, lipid peroxidation assay, TEM analysis, cellular respiration assay, and calcium imaging findings suggest that EC359 treatment induces cell death in OCa cells through a multifaceted mechanism involving alterations in glycolysis, mitochondrial function, and calcium signaling. However, the possibility of involvement of other LIFR downstream signaling pathways in regulation of ferroptosis defense system or direct targeting of components of GSH antioxidant defense system by EC359 has yet to be explored.

LIF/LIFR signaling contributes to altered TME^15–17^ and plays a role in modulating multiple immune cell types present in TME including effector T cells, regulatory T cells, macrophages^17^, and myeloid cells which lead to immune suppression^18^. LIF regulates CXCL9 in tumor-associated macrophages and prevents CD8^+^ T cell tumor-infiltration impairing anti-PD1 therapy. The combination of LIF neutralizing antibodies with the inhibition of the PD1 immune checkpoint promotes tumor regression, immunological memory, and an increase in overall survival^54^. It was demonstrated that LIF mediate M2 macrophage repolarization and inhibited T cell function in gastric cancer^55^. LIF treatment is shown to promote macrophages to acquire immunosuppressive capacity^56^. Interestingly, MSC-1 (a humanized monoclonal antibody that binds to LIF) treatment drove TAMs to obtain antitumor and proinflammatory function in syngeneic colon cancer mouse models^56^. Moreover, phase I clinical trial of MSC-1 for advanced solid tumors showed that the treatment increased M1:M2 ratio and decreased levels of STAT3 phosphorylation^57^. Our studies using EC359 also demonstrated the beneficial effect of blocking LIF signaling in modulating TME including enhanced recruitment of cytotoxic T cells and increased M1:M2 ratio. These data imply that EC359 has the potential to remodel the TME and enhance anti-tumor immune responses. Therefore, targeting LIF/LIFR axis can be a promising approach to suppress resistance to immunotherapy and improve efficacy of immune checkpoint blockade to broaden the clinical utility of immunotherapy^58^. However, future studies are needed to examine the beneficial effect of EC359 combination therapy with immunotherapy.

In summary, our data indicates that pharmacological inhibition of a positive autocrine loop of LIF/LIFR promotes ferroptosis and can inhibit OCa proliferation in vitro and tumor progression in vivo. Our results also offer EC359 as a new tool for targeted therapy of OCa and to improve patient’s outcome.

## Methods

### Cell culture and reagents

OVCAR3 (HGSOC), ES2 (LGSOC), SKOV3 (CCOC), TOV21G (CCOC), TOV112D (ENOC), OV90 (MOC), COV644 (MOC), HEK-293T cells were received from the American Type Culture Collection (ATCC, Manassas, VA). OVSAHO (HGSOC) was purchased from AcceGen™ and cultured using RPMI-1640 medium supplemented with 10% FBS (Sigma) and Gibco™ Antibiotic-Antimycotic. OVCAR8 (LGSOC) and OVCAR5 (HGSOC) cell line was purchased from NCI DCTD repository. IGROV1 (ENOC) cell line was procured from Dr. Sood (MD Anderson Cancer Center, Houston). ID8agg, an epithelial mouse serous ovarian cancer cell line (C57BL/6 background) was cultured in RPMI-1640 medium supplemented with 10% FBS^59^. Recent publications were used to classify existing cells into subtypes^60,61^. OCa tissue-derived primary cells, organoids, explants, xenografts (OCa1, OCa2, OCa9, OCa10, OCa14, OCa27, OCa30, OCa38, OCa39, OCa45, OCa50, OCa66, and OCa73), ascites derived cells (AS20, 21, 23, 25, 28, and 29) and primary human endometrial stromal cells (HESC) were obtained from the Ob/Gyn tissue core that had received IRB approval (Supplementary Table 1). Immortalized nontumorigenic ovarian surface epithelial-derived cell line (IOSE-80) was previously described^62^. There was no mycoplasma infection in any of the model cells used. The identity of the cells was also verified by STR DNA profiling. All antibodies used in this research were purchased from Cell Signaling Technology except LIF, LIFR (Santa Cruz), and Vinculin (Sigma) (Supplementary Table 2). The Ki67 antibody (ab1667) was purchased from Abcam (Cambridge, MA). LIFR-KO OCa cells were generated using lentiviral transduction of Cas9 and by using validated LIFR targeting gRNAs as described^34^. Constitutively active STAT3 was overexpressed using Stat3-C Flag pRc/CMV plasmid (Addgene plasmid # 8722; RRID:Addgene_8722)^63^.

### Cell viability, colony formation, and cell death assays

The cell viability rates of the control and EC359 treated cell lines were assessed by MTT assays as described^64,65^. Effect of EC359 on colony formation and cell death was determined using established methods as described^34^.

### Lipid peroxidation assay

Lipid peroxidation was analyzed by flow cytometry. Briefly, cells were seeded at a density of 2 × 10^5^ per well in 60 mm petri dishes and grown overnight to let them attach in RPMI 1640 medium supplemented with 10% FBS and antibiotics. Cell plates were treated with vehicle (DMSO) or EC359 (100 nM) for 12 h. BODIPY™ 581/591 C11 reagent 10 mM (Image-iT™ Lipid Peroxidation Kit, Invitrogen™) was added to the cell plates (10 μM final concentration) 2 h before analysis. Then, cells were washed with PBS, treated with trypsin and resuspended in culture medium for flow cytometry analysis. The flow cytometer was used to analyze the oxidation of BODIPY C11, which is detected by a shift in the fluorescence emission peak from red (590 nm) to green (510 nm).

### Western blotting

OCa cells were subjected to cell lysis using RIPA buffer containing protease and phosphatase inhibitors followed by Western blot analysis. For the autocrine loop studies, 70–80% confluent 100 mm plates of cells were washed twice with PBS and serum free RPMI-1640 medium was added to the plates. Cells were incubated in humidified CO_2_ incubator for 48 h. Next, conditioned medium (CM) was collected and centrifuged at 3000 RPM, for 5 min to spin down cell debris. Then conditioned medium was concentrated ~20 times using Amicon® Ultra-4 Centrifugal Filter Units (10,000 Dalton molecular weight cutoff). 40 μl of the concentrated CM was used to perform Western blotting. Cells cultured in 10% FBS were used for LIFR expression using Western blotting. All blots or gels derive from the same experiment and that they were processed in parallel. Uncropped scans of the most important blots were included as Supplementary Figure in the Supplementary Information.

### ALDEFLUOR, sphere formation, limiting dilution assays, and quantitative real time-PCR

Cancer stem cells (CSCs) were isolated based on their aldehyde dehydrogenase (ALDH) enzymatic activity using the ALDEFLUOR™ kit (STEMCELL Technologies), according to the manufacturer’s instruction. We used FACSAria cell sorter (BD Biosciences) to separate brightly fluorescent ALDH-expressing cells. In brief, 1 × 10^6^ cells were suspended in 1 ml ALDEFLUOR buffer and 1 μl ALDEFLUOR reagent for 30 min at 37 °C. ALDH1A1-bright cells were detected in the green fluorescence channel. Specific ALDH1A1 inhibitor diethylaminobenzaldehyde (DEAB) was used as negative control to set the gates defining the ALDH1A1-positive region. Next, cells were washed and cultured in DMEM–F12 medium (Gibco™) containing B27 supplement (Gibco™), 20 ng/ml EGF, 10 ng/ml bFGF (R&D Systems) and antibiotics as cancer stem cell medium. In the sphere formation assay, ALDH^+^ cells were cultured in cancer stem cell medium, and 50 cells were seeded on 96 well ultra-low attachment plates (Corning) in six replicate wells. Two weeks later, the number of spheres formed was recorded, and sphere dimensions were measured using ImageJ software, and cell viability was quantified using CellTiter-Glo® reagent according to the manufacturer (Promega). For extreme limiting dilution assay, CSCs were seeded in decreasing numbers (50, 20, 10, 5, and 1 cells/well) in 96 well ultra-low attachment plates and treated with vehicle or EC359. After 10 days, the number of wells containing spheres per each plating density was recorded, and stem cell frequency between vehicle and treatment groups was calculated using ELDA analysis software (https://bioinf.wehi.edu.au/software/elda/)^66^. Reverse Transcription quantitative PCR (RT-qPCR) was used to validate a subset of genes, together with total RNA extracted from OV90 and ES2 CSCs. Primer sequences are shown in Supplementary Table 3. RT-qPCR was performed in CFX96 Real-Time PCR System (Bio-Rad) using SYBR Green Master Mix (Applied Biosystems™). The delta-delta-CT approach was used to calculate the difference in fold change after normalizing the data to GAPDH or β-Actin.

### RNA sequencing and analysis

Following the manufacturer’s instructions, total RNA was extracted from ES2 cells that had been treated with EC359 (10 nM) for 12 h (Qiagen, Valencia, CA). RNA sequencing and analysis were conducted by UT Health San Antonio sequencing core using established protocols^64,65^. RNA-seq data that has been deposited with GEO accession number GSE236743. The raw reads were aligned to the reference human genome (UCSC hg19) with TopHat2^67^. Genes were annotated (using NCBI RefSeq) and quantified by HTSeq^68^, and DESeq^69^ was used to identify differentially expressed genes and significant genes with fold change > 1 and multiple-test adjusted *p* value < 0.01 were used for interpreting the biological pathways using IPA.

### Reporter assays

STAT3 Firefly Luciferase Reporter Lentivirus (Kerafast) was used to generate stable STAT3-luciferase cells. Antibiotic selection was done using Puromycin and Luciferase activity of control and EC359 treated STAT3-Luciferase^+^ cells (100 nM for 6 h) were measured using ultra-sensitive luminometer (EG&G Berthold).

### Immunohistochemistry (IHC)

IHC using Ki67 was done as described previously^70^. The proliferative index was calculated using Ki67 positive cells in five randomly selected microscopic fields at 20X per slide.

### OCR and ECAR assay

The oxygen consumption rate (OCR) and extracellular acidification rate (ECAR), of OVCAR3 cell line were determined by Agilent Seahorse XF Pro Analyzer using Agilent XF Cell Mito Stress Test kit (Agilent, Santa Clara, CA, USA) according to the manufacturer’s instructions. Briefly, OVCAR3 cells were seeded in Seahorse XF Pro plates at 30,000 cells/well. Cells treated with EC359 (100 nM) for 0 h, 1 h, 3 h, and 12 h were used for Seahorse assay. Firstly, the OVCAR3 cell medium was replaced by the conditional Agilent DMEM medium (medium without FBS and sodium bicarbonate, but with added 1 mM pyruvate and 2 mM glutamate) and incubated in CO_2_-free incubator for 1 h before completion of probe cartridge calibration. Secondly, the baseline of OCR and ECAR were recorded. Measurements were performed after the injection of three compounds affecting bioenergetics: 1 µM oligomycin (Sigma, St Louis, MO, USA), 1 µM carbonyl cyanide 4-(trifluoromethoxy) phenylhydrazone (Sigma) and 1 µM Rotenone + 1 µM antimycin A (Sigma). Upon completion of the Agilent XF Cell Mito Stress Test analysis, cells were lysed to calculate the protein concentration using a Bio-Rad Protein Assay kit (Bio-Rad, Hercules, CA, USA). The result was normalized with the protein OD value of the corresponding well.

### Intracellular calcium ([Ca2^+^]i) measurement

Cells were grown on 35 mm glass-bottomed culture dishes (MatTek, Ashland, MA) overnight at 37 °C in a tissue culture incubator. Cells were supplemented with fresh media and treated with 2 mM Fura-2-AM (Abcam) for 45 min. To evaluate intracellular Ca2^+^ the culture dishes were gently washed with Ca2^+^-free SES buffer and placed under the microscope for calcium measurement. Changes in Fura2-AM fluorescence in single cells were measured by the addition of Thapsigargin (1 mM) in a Ca2^+^-free SES buffer using a TILL Photonics spectrofluorometer (TILL Photonics In., Eugene, OR). To measure ER calcium release Thapsigargin (2 mM) was added in the absence of external calcium. For calcium entry 1 mM Ca2+ was added and images were acquired every 5 s. A ratio of 340/360 was used to measure changes in cytosolic calcium levels and quantified from 50 to 200 cells for each condition.

### Maximum tolerated dose (MTD) and dose range finding studies

To identify MTD, target organs of toxicity and the toxicokinetic profile of EC359, we conducted the toxicity of EC359 by single and by repeated oral administration for 7 consecutive days to Sprague Dawley (SD) rats using a commercial company (Vimta Labs, Hyderabad, India). During the single dose phase, two males and two females per group were used. Six doses were administered in the MTD phase in a stepwise manner. Dosing was initiated with 2.5 mg/kg/dose followed by escalating doses of 5, 15, 50, 100 and 300 mg/kg/dose to separate groups of animals respectively. During repeated dose phase (7 days repeated administration), eight groups (G1B to G4B [main study] and G1BTK to G4BTK) of SD rats were used. Groups G1B to G4B (main groups) comprised 4 males and 4 females per group which were used for toxicity evaluations. Groups G2BTK to G4BTK comprised 12 males and 12 females and Group 1BTK comprised 3 males and 3 females which, served as satellite groups and were used for toxicokinetic (TK) evaluation. Groups G1B and G1BTK were treated with the vehicle control alone for 7 consecutive days (Supplementary Fig. 4a). Due to unexpected clinical signs at 100 mg/kg (G4B and G4BTK), dosing was discontinued on day 5 and all surviving animals were sacrificed early on Day 6. Dose formulations were prepared in a vehicle containing 58.5% Labrasol ALF, 22.5% Labrafil M, 1944 CS, 9% Capryol 90 and 10% water q.s. TK evaluations were carried out for all satellite TK groups on day 1 of treatment. However, on Day 7, TK evaluation was carried out in animals treated at 10 and 30 mg/kg/dose only, as the other groups were terminated on Day 5 of treatment due to severe toxicity.

### In vivo orthotopic tumor model

All animal studies were carried out once we received UTHSA IACUC approval. Charles River supplied us with female SCID mice aged 6–8 weeks. For xenograft investigations, ES2 (1 × 10^5^ cells) and ID8 (4 × 10^6^) cells stably expressing GFP-Luciferase were intraperitoneally injected into female SCID mice. Following the development of tumors, treatment groups for either the vehicle or EC359 were assigned at random. The number of mice required to show treatment impact was determined based on our pre-existing data as well as published findings. The calculations are based on the unpaired data model, power = 0.8, *p* value = 0.05. Using the Xenogen in vivo imaging technology, tumor growth in mice was monitored. The body weight, tumor weight, number of metastatic tumor nodules, and volume of the ascites fluid were measured at the completion of the treatment period. At the end of the experiment, mice were anesthetized with isoflurane inhalation followed by cervical dislocation.

### In vivo CDX and PDX tumor models

PDX tumors were procured from the Ob/Gyn tissue core. Female SCID mice were used as the recipients of 2 mm^3^ implants containing CDX (SKOV3, OVCAR3) or PDX (OCa10, OCa14, OCa30, OCa38) tumor tissue for ectopic tumor model experiments. When tumors were detectable in size, mice were split between control and treatment groups (each group having *n* = 6 to 8 tumors). The EC359 (2.5, 5, 7.5, or 10 mg/kg either sub cutaneous (s.c.), intraperitoneal (i.p.) or oral) was administered to the treatment groups while the vehicle (s.c. and i.p. in 0.3% hydroxypropyl cellulose or oral gavage in 30% Captisol) was given to the control group. Toxicological consequences on the mice were observed every day. Using a digital caliper, tumor development was monitored every 3–4 days. To prepare the tumors for IHC staining, they were removed, weighed, and processed after the mice were euthanized at the conclusion of each experiment. At the end of the experiment, mice were anesthetized with isoflurane inhalation followed by cervical dislocation.

### Patient-derived explant and organoid studies

Excised cancer tissues were processed and grown ex vivo for patient-derived explant (PDEx) experiments as previously described^34,36^. The study was conducted in accordance with the Declaration of Helsinki and approved by the Institutional Review Board (or Ethics Committee) of University of Texas Health San Antonio (HSC20190695N and 9 March 2019). Following IRB approval, de-identified OCa tissues were collected from the UTHSA Ob/Gyn after written informed consent from patients. Tumor samples were removed and chopped into small pieces before being incubated for 24 h on gelatin sponges in culture media containing 10% FBS. Tissues were treated with vehicle or EC359 in culture media for 72 h before being fixed in 10% buffered formalin overnight at 4 °C and processed into paraffin blocks. Following that, sections were processed for Ki67 immunohistochemical examination. Patient-derived organoids (PDO) were grown from de-identified OCa tissues according to the ATCC culture instructions (https://www.atcc.org/en/Guides.aspx). PDOs were collected from Matrigel and dissociated into single cells using Organoid Harvesting Solution (Cultrex™) and mechanical dispersion for cell viability experiments. The cell suspension was resuspended in 70% Matrigel before seeding 5 × 10^3^ cells/10 µl drop per well of a 96-well plate. After adding culture medium, organoids were allowed to grow for one week. The organoid cultures were subjected to a concentration dilution series of EC359 in triplicate. Cell viability was determined after 7 days of treatment using the Promega® CellTiter-Glo® 3D-Superior Cell Viability Assay reagent (Promega, Madison, WI, USA). The luminescence intensity was measured using a GloMax® Discover System (Promega, Madison, WI, USA).

### Lymphocyte preparation and analysis by flow cytometry

Single-cell suspensions were prepared from pooled and minced ID8 tumors, ascites, draining mesenteric lymph nodes and the spleen using a 70 μm cell strainer. Cells were collected in RPMI-1640 medium supplemented with 10% FBS, 1% penicillin–streptomycin/amphotericin B and resuspended in ACK Lysis Buffer (Lonzo) to lyse red blood cells. After quenching with RPMI-FBS, cells were resuspended in PBS for immediate staining and flow cytometry analysis. In most experiments involving tumor-infiltrating lymphocytes, leukocytes from ID8 tumors underwent additional enrichment by a Ficoll-Paque gradient (GE Healthcare, Cat. # 17-0891-01) following the manufacturer’s instructions.

For staining, cells were first stained for 20 min in Hank’s Buffered Salt Solution plus 0.1% bovine serum albumin (BSA-HBSS) with fluorochrome-labeled mAbs to surface markers in the presence of mAb Clone 2.4G2, which blocks FcγIII and FcγII receptors, and 7-AAD or a fluorochrome-conjugated fixable viability dye (FVD, Supplementary Table 4). After washing, cells were either resuspended in HBSS for FACS analysis in an LSRII (BD) or proceeded for intracellular staining of phosphorylated STAT1 (pSTAT1) and cMAF. For intracellular staining, cells were resuspended in the BD Cytofix/Cytoperm™ buffer (250 μl) and incubated at 4 °C for 20 min. After washing twice with the BD Perm/Wash™ buffer, cells were counted again, and 1 × 10^6^ cells were resuspended in 100 μl of BD Cytofix/Cytoperm™ buffer for staining with fluorochrome-labeled anti-pSTAT1 and/or anti-cMAF mAbs at 4 °C for 30 min (Supplementary Table 4). After washing with BD Perm/Wash™ buffer, cells were analyzed by FACS in an LSRII. All data were analyzed by FlowJo^®^ (BD).

### Databases and statistical analyses

In order to examine alterations in the levels of LIF, LIFR, and OSM in OCa, we employed the TNM plot analysis tool (https://tnmplot.com/analysis/)^20^. This tool utilizes a validated database to evaluate variations in gene expression between tumor and normal tissues. OCa patients’ progression-free survival was analyzed using a Kaplan Meier plotter^19^. A database containing ROC plotter was utilized to compare the expression levels of chemotherapy-resistant and sensitive malignancies^71^. Statistical differences between groups were analyzed with unpaired Student’s *t* test and one-way or two-way ANOVA using GraphPad Prism 9 software. All the data represented in plots are shown as means ± SE. A *p* value of *p* < 0.05 was considered statistically significant.

### Reporting summary

Further information on research design is available in the Nature Research Reporting Summary linked to this article.

### Supplementary information

Supplementary material

Reporting summary

## Data Availability

All the data produced in this investigation is incorporated in the primary paper as well as in supplementary material. The corresponding author will handle and fulfill all reasonable requests for resources and reagents. The materials and data will be provided upon request once a material transfer agreement has been finalized. RNA-seq data that has been deposited with GEO accession number GSE236743.

## Code availability

All relevant codes are available from the corresponding authors upon reasonable request.
