# Supplementary material for: Pharmacological inhibition of the LIF/LIFR autocrine loop reveals vulnerability of ovarian cancer cells to ferroptosis
Source: NPJ Precis Oncol. 2024 May 24;8:118. doi: 10.1038/s41698-024-00612-y (PMC11126619; doi:10.1038/s41698-024-00612-y)
Supplement: Supplementary file 1 — Supplementary material [file 41698_2024_612_MOESM1_ESM.pdf]

Supplementary Fig. 1

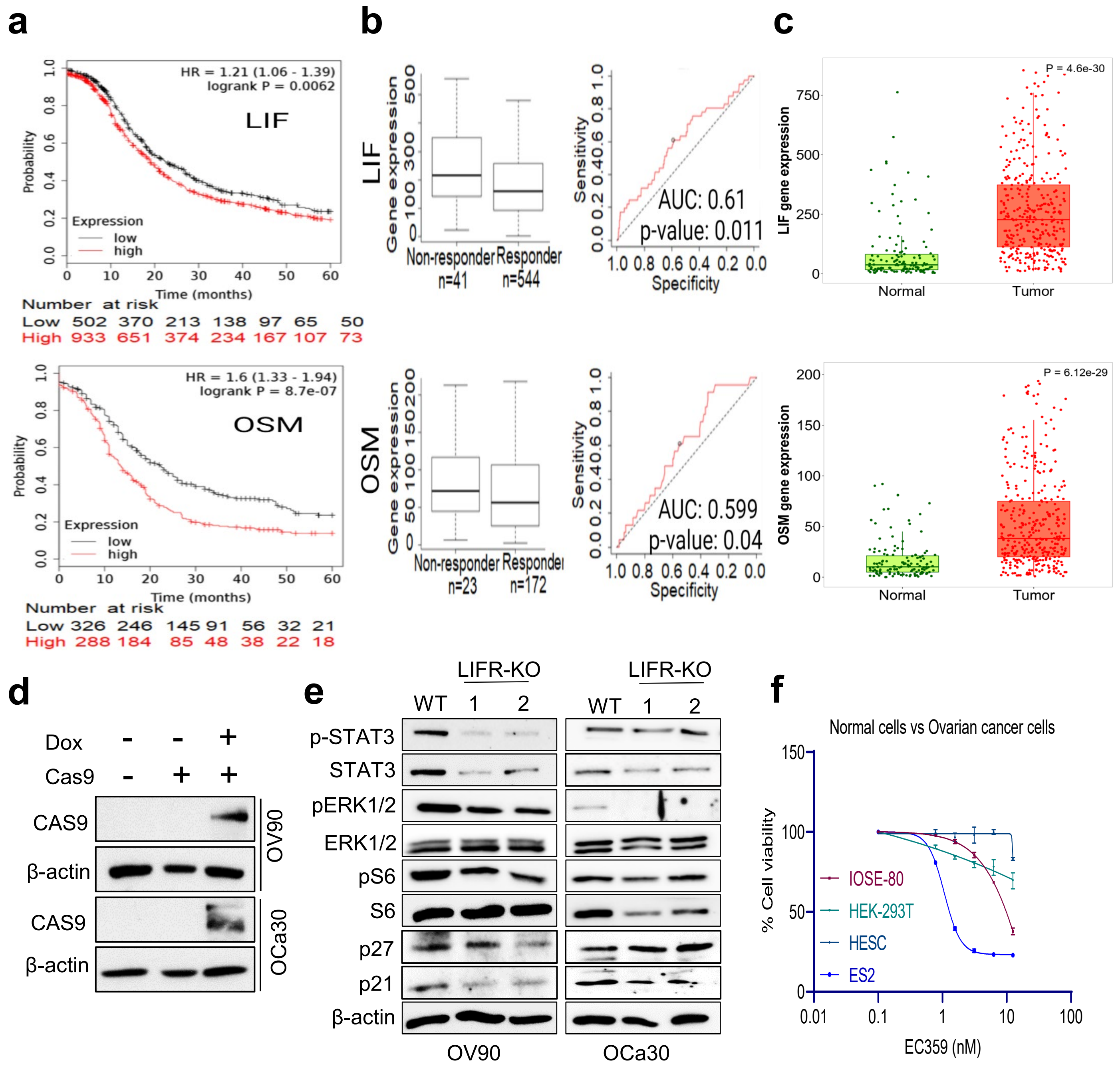

Supplementary Fig. 1. a, Kaplan-Meier survival analysis of OCa stratified by LIF and OSM gene expression levels. **b**, Box plots and ROC curves of LIF and OSM were generated using RFS at 6-month cohort. Only samples with serous histology (grade 3) and those treated with platinum and taxane combined therapy were included in the analysis. **c,** A boxplot of LIF and OSM gene expression when comparing paired normal (n=133) and OCa tumors (n=374) gene array data based on TNMplot database. **d**, Western blot representation of Doxycycline inducible expression of Cas9 enzyme in OV90 and OCa30 empty vector (first column), and stable iCas9 cell lines in the absence (middle column) and presence of Doxycycline (right column). **e,** The effect of LIFR-KO with two sgRNAs targeting LIFR in OV90, and OCa30 cell lines on downstream signaling and levels of p27, p21 are shown using Western blotting. f, Effect of EC359 on the cell viability of normal human ovarian epithelial cells (IOSE), human endometrial stromal cells (HESC), human epithelial kidney cells (HEK-293T) in comparison with OCa cell line ES2 measured by MTT assay.

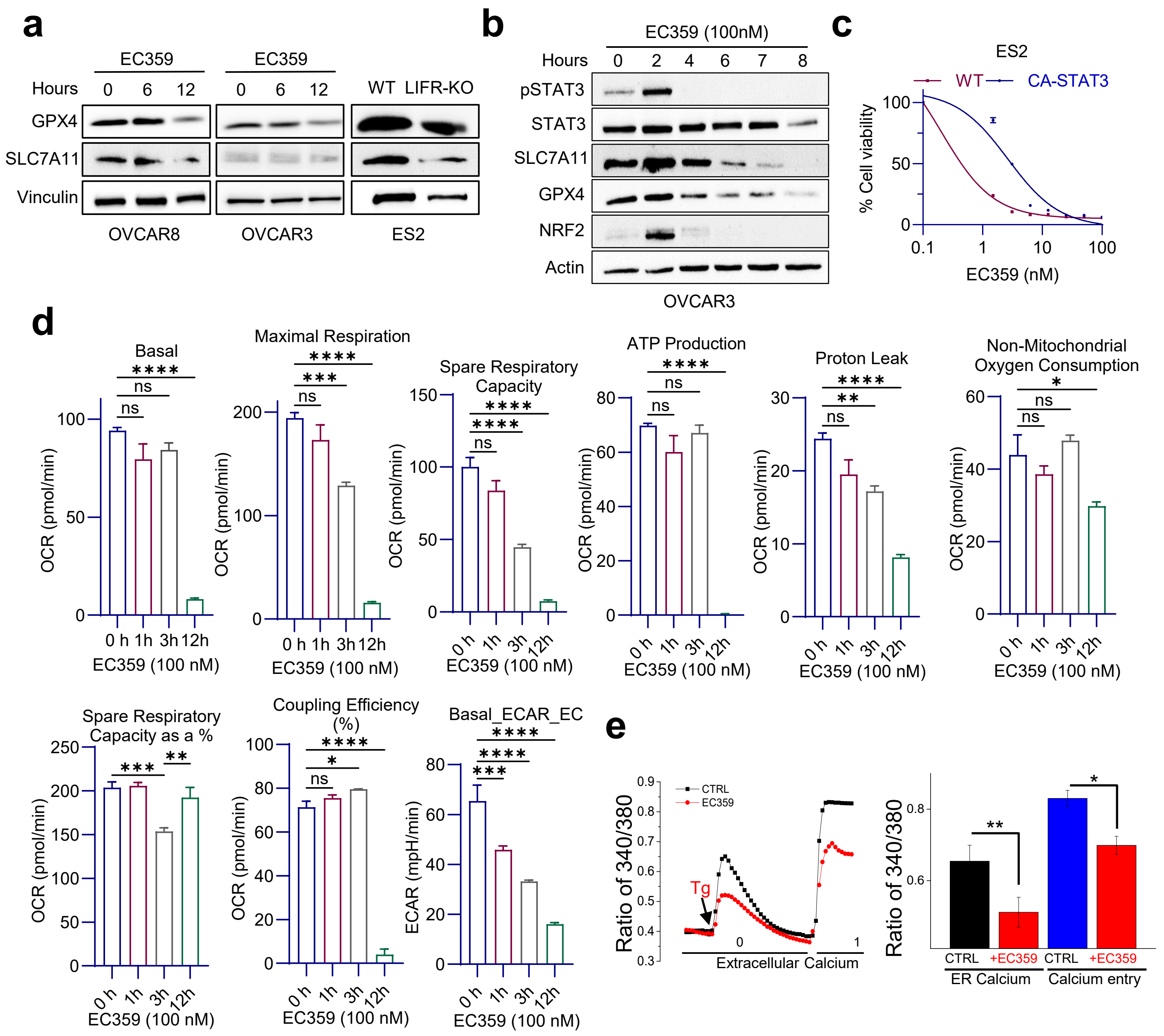

Supplementary Fig 2. a, Western blot presentation of changes in the GPX4, and SLC7A11 expression in OVCAR3, OVCAR8 cells when treated with EC359 (20 nM) for 6 and 12 hours as well as in ES2 LIFR-KO cells. b, Kinetics of inhibition of STAT3 and ferroptosis markers after EC359 (100 nM) treatment was analyzed by Western blotting. **c**, Cell viability of ES2 wildtype (WT) and ES2 cells overexpressing constitutively active STAT3 (CA-STAT3) treated with a serial dilution of EC359 for 4 days measured using MTT assay. **d,** Quantification of basal respiration, maximal respiration, spare respiratory capacity, ATP production, proton leak, and non-mitochondrial oxygen consumption. Quantification of basal ECAR. The 6 key parameters for mitochondrial respiration were calculated from OCR. **e,** Individual calcium traces in control and EC359 treated cells (3 hrs.) showing changes in intracellular calcium levels. Quantification of ER calcium release and calcium entry in control and EC359 cells (50-200 cells) are shown as a bar graph. *p<0.05, **p<0.01, *** p<0.001 and **** p<0.0001 as determined by ordinary one-way ANOVA test. n = 3-4 in each group.

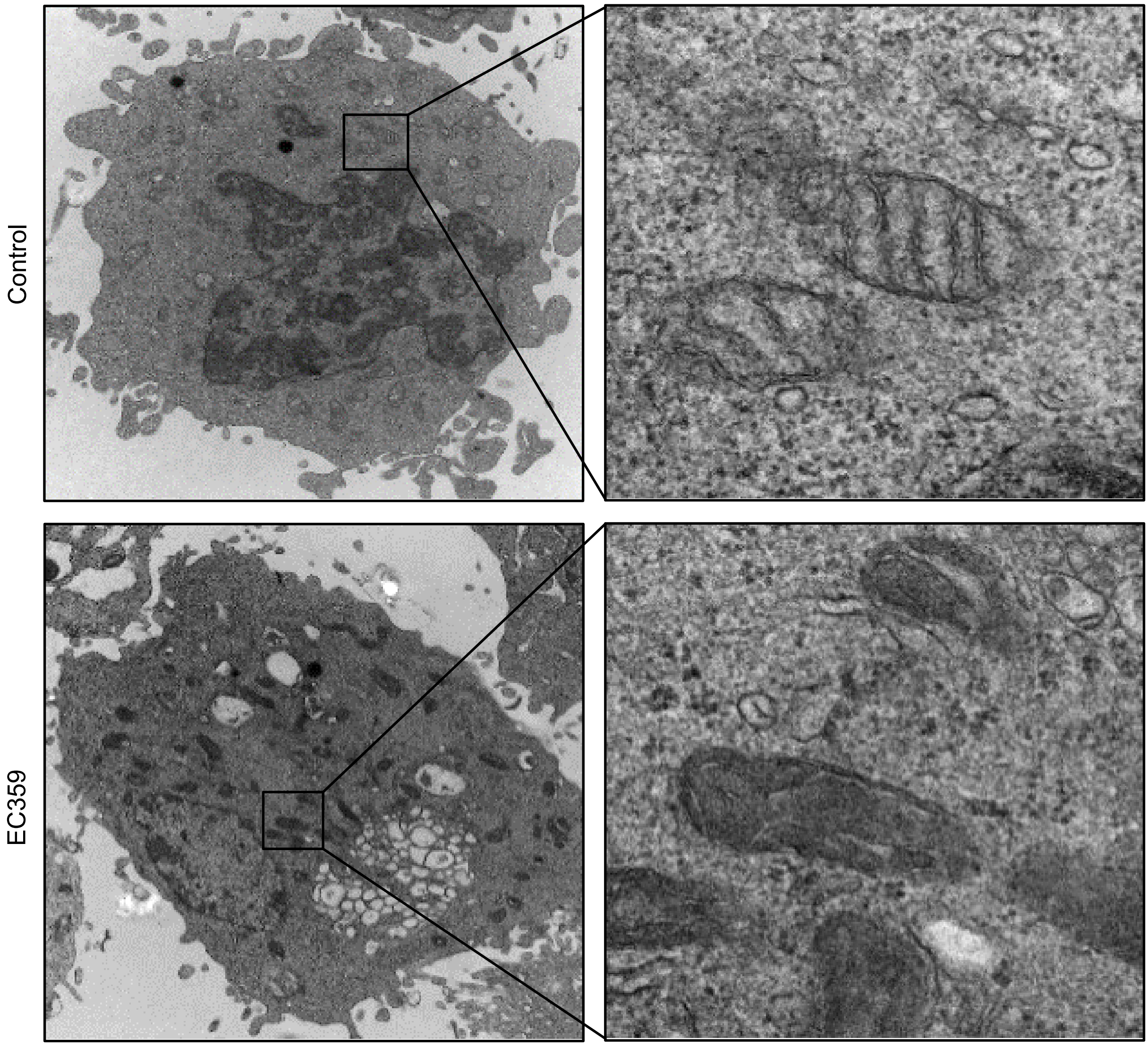

Supplementary Fig 3. Transmission Electron Microscopy images of OVCAR3 cells treated with vehicle or 100 nM EC359 for 9 hours. Magnification 10,000X (left images) and 100,000X (right images)

a

Treatment and Dose levels	MTD Phase: Single dose escalation phase<br>G1A: 0 mg/kg b.wt./dose<br>G2A: 2.5 mg/kg b.wt./dose<br>G3A: 5 mg/kg b.wt./dose<br>G4A: 15 mg/kg b.wt./dose<br>G5A: 50 mg/kg b.wt./dose<br>G6A: 100 mg/kg b.wt./dose<br>G6A1: 300 mg/kg b.wt./dose<br>DRF Phase: 5 to 7 Day repeated administration<br>Groups G1B and G1BTK: 0 mg/kg b.wt./dose<br>Groups G2B and G2BTK: 10 mg/kg b.wt./dose<br>Groups G3B and G3BTK: 30 mg/kg b.wt./dose<br>Groups G4B and G4BTK: 100 mg/kg b.wt./dose
Number of Animals	MTD Phase: 28 (14 males & 14 females; 2 animals/sex/group)<br>DRF Phase: 110 (55 males & 55 females; Main group: 4/sex/group; TK group: 12/sex/group: TK control group; 3/sex)

b

TK parameters	Day 1					
	10 mg/kg/dose		30 mg/kg/dose		100 mg/kg/dose	
	Males	Female	Males	Female	Males	Female
T_{max}	8.00	4.00	8.00	4.00	8.00	4.00
C_{max}	1050.48	1311.64	3210.14	3239.51	5231.40	4318.10
AUC_{0-t} (ng.h/mL)	14327.50	16033.17	56675.93	41309.39	72830.04	42118.85

TK parameters	Day 7				Day 5	
	10 mg/kg/dose		30 mg/kg/dose		100 mg/kg/dose	
	Males	Female	Males	Female	Males	Female
T_{max}	8.00	4.00	8.00	4.00	8.00	4.00
C_{max}	486.60	1351.83	1682.33	2887.09	1436.31	1806.75
AUC_{0-t} (ng.h/mL)	6265.67	15355.54	21973.82	35970.07	23544.60	20899.98

c

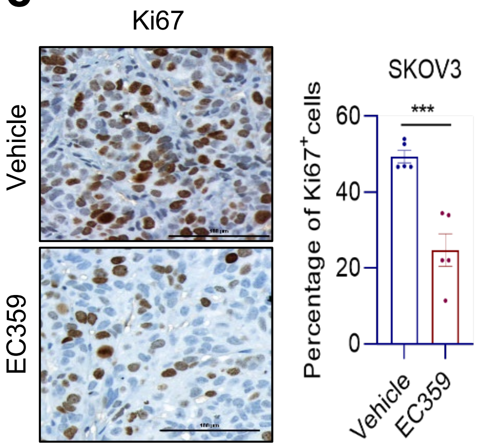

d

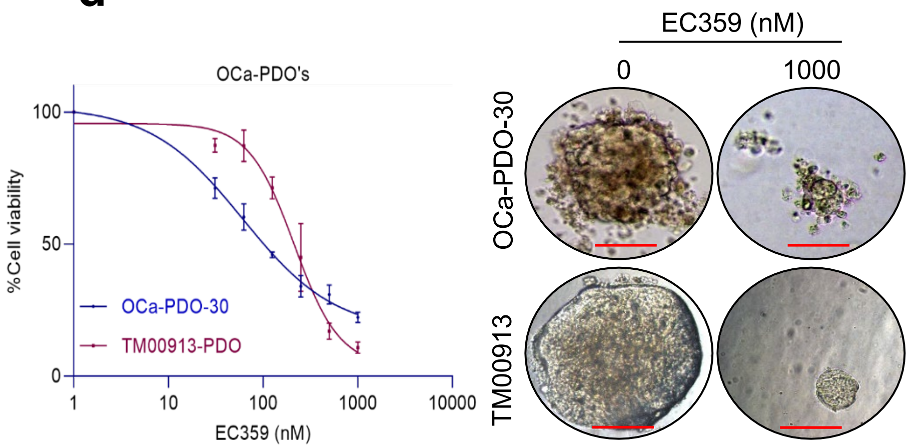

Supplementary Fig. 4. a, List of the doses and animals used for MTD and dose range finding studies. b, toxicokinetic data was listed. c, Images of SKOV3-CDX models immune-stained for Ki67 proliferative marker. Bar graphs represent comparisons of the percentages of Ki67 positive cells in the vehicle or EC359 treated xenografts. d, CellTiter-Glo® assay showing the effect of indicated dose of EC359 on OCa-PDO's for 7 days (Left panel). Representative images of OCa-PDO's treated with or without EC359 for 7 days are shown (Right panel). Scale bar represents 100 µm.

Supplementary Fig. 5

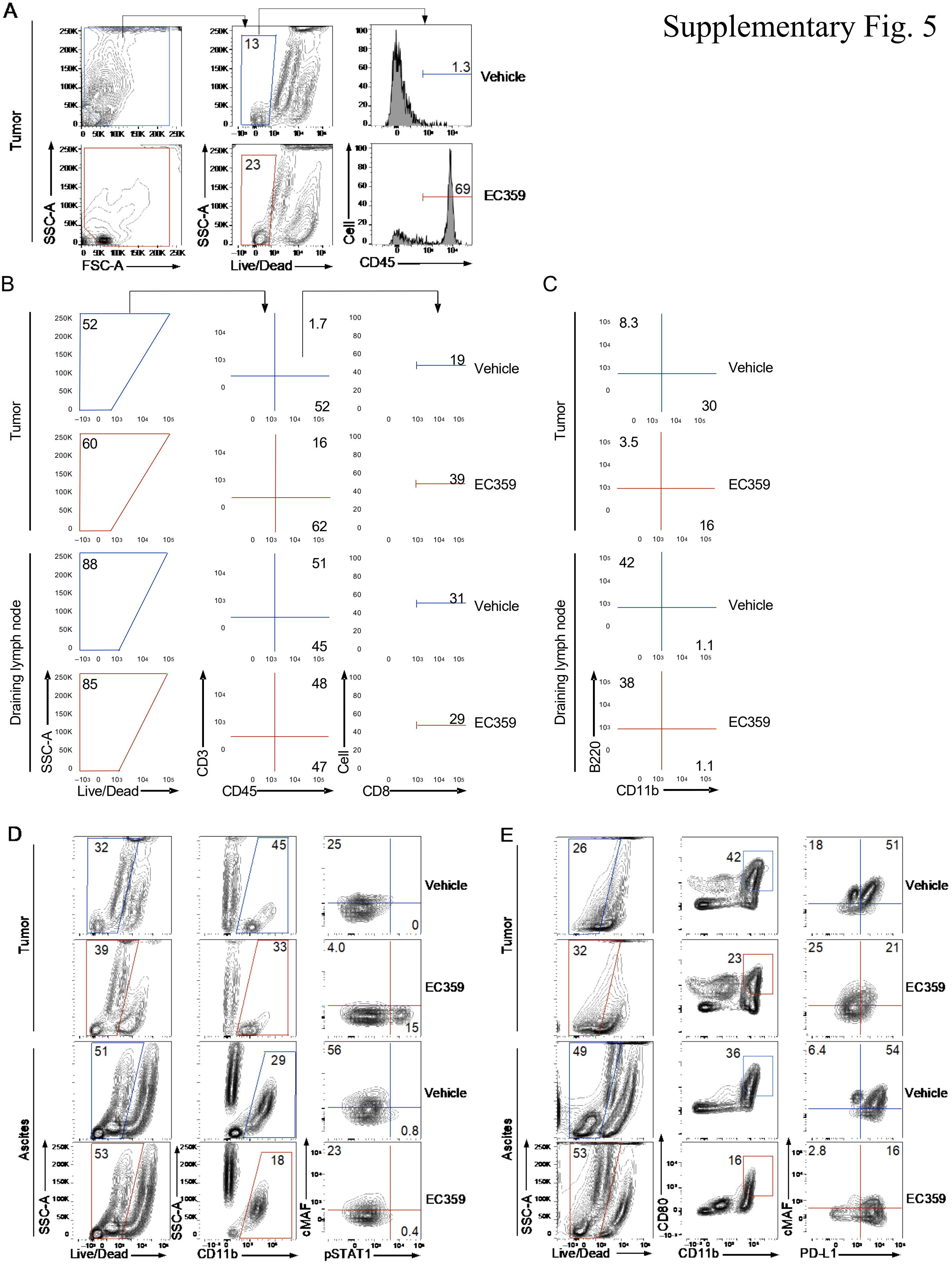

F

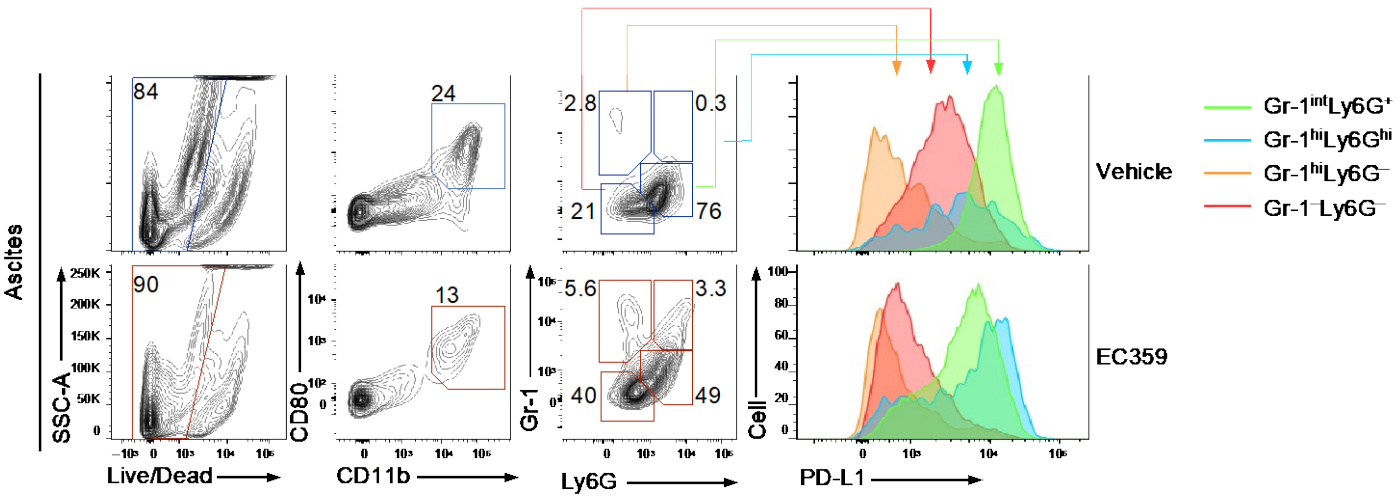

Supplementary Fig. 5. EC359 treatment induces lymphocyte alterations in the tumor microenvironment and external environment in a syngeneic OCa mouse model. A, flow cytometry analysis of ($CD45^+$) leukocyte infiltration into ID8 tumors in vehicle-treated C57BL/6 mice or residual tumors in EC359-treated mice without prior leukocyte enrichment. **B,** flow cytometry analysis of the proportion of $CD3^+$ T cells (middle panels) within ($CD45^+$) leukocytes that infiltrated into (residual) ID8 tumor-infiltrating leukocytes (after pre-enrichment with Ficoll-Paque) or were present in draining mesenteric lymph node, as indicated, as well as the proportion of $CD8^+$ T cells within $CD3^+$ cells (right panels) in mice treated with vehicle or EC359. **C,** flow cytometry analysis of the proportion of $CD11b^{hi}B220^-$ myeloid cells and $CD11b^-B220^+$ B cells in ($CD45^+$) leukocytes in ID8 tumors or lymph node, as in **B**. **D,** flow cytometry analysis of the proportion of $CD11b^+$ cells that expressed phosphorylated STAT1 (pSTAT1), a hallmark transcription factor of pro-inflammatory M1 macrophages, or cMAF, a hallmark transcription factor of anti-inflammatory M2 macrophages, in (residual) ID8 tumors (after pre-enrichment with Ficoll-Paque) or ascites in mice treated with vehicle or EC359. **E,** flow cytometry analysis of the expression of cMAF and the PD-L1 immune checkpoint in $CD11b^+CD80^{hi}$ myeloid cells in ID8 tumors or ascites, as in **D**. **F,** flow cytometry analysis of the proportion of $CD11b^+CD80^{hi}$ myeloid cells in ascites and the spleen (the second panels) as well as of the 4 subsets based on the expression of Gr1 and Ly6G, as indicated (the third panels) and their respective PD-L1 expression levels (the fourth panels) in ID8 cell-engrafted mice treated with vehicle or EC359. The $Gr\text{-}1^{int}Ly6G^+$ subset displayed the highest proportion and PD-L1 expression in ascites

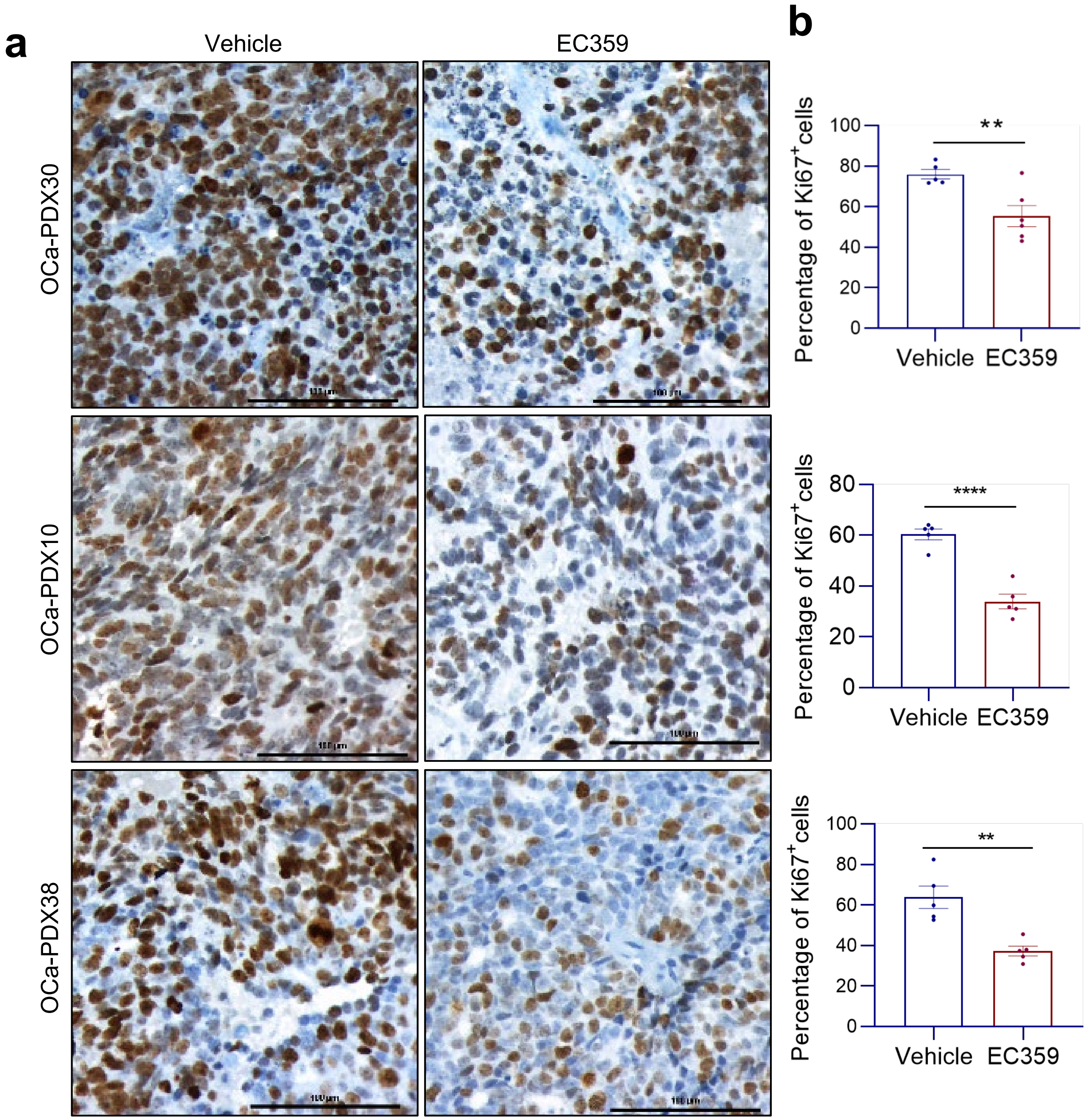

Supplementary Fig. 6. a, IHC images of OCa-PDX30, OCa-PDX10 and OCa-PDX38 models immune-stained for Ki67 proliferative marker. **b,** Bar graphs represent comparisons of the percentages of Ki67 positive cells in the vehicle or EC359 treated xenografts.

Supplementary Table 1.
List of primary OCa cells and tumors used for the study

Number	Histology	Grade	Stage	Stains
OCa1	High-grade serous	HIgh	IIIC	Not available
OCa2	High grade serous	High	IIIC	Not available
OCa9	Serous- LMP w/HGS	Low	IIIA	Ki67 low rate in low grade portions, but markedly increased in high grade portions, P53 and p16 shows diffuse staining in LMP areas, WT1 diffusely positive
OCa10	Neuroendocrine	G3	IIIC1	Positive for synaptophysin and CD56 diffusely positive, chromogranin negative
OCa14	Endometrioid	3	IB	Positive for CK7, ER, PR< CK5/6 and CA125, Neg for CK20, GATA-3, p53 and p63
AS20	High grade serous	High	IIIC	Positive for PAX8, WT-1 and P53(strong and diffuse), Neg for Napsin
AS21	High grade serous	High	IIIC	Positive for CA125, ER, PAX8, WT1 and P53, Neg for p16
AS23	Endometrioid	3	IC	Positive for CA125, p53, ER (weak), PR focally pos, Neg for vimentin
AS25	High grade serous	High	IIIC	Positive for WT1, PAX8, ER and P16, Neg for p53 and napsin
OCa27	High grade serous	High	IIIC	Positive for WT1(patchy), P16, ER(weak), Negative for p53, chromogranin, synaptophysin
AS28	High grade serous	High	IV	Positive for p53, WT1, ER and PAX8, Calretinin highlights mesothelial cells
AS29	High grade serous	High	IIIC	Positive for CA125, PAX8 and WT1, Neg for ER and p53
OCa30	High grade serous	High	IIIC	Not available
OCa38	Serous papillary- High grade	High	IVB	Not available
OCa39	High grade serous	High	IB	Positive for WT1 and p53, CA125(luminal staining)
OCa45	High grade serous	High	IIIC	Positive for ER, WT1, PAX8, CA125, CK7 (weak), Neg for CK20 and P53
OCa50	High grade serous	High	IIIC	Positive PAX8, WT1, ER, CK7 focally positive, CK20 negative
OCa66	Mucinous adenocarcinoma	Low	IA	PDL1 0%, CDKN2A loss, CDKN28 loss, KRAS G12D, MTAP loss, MYC amp, STK11 loss, TP53 splice site 672+1G>A
OCa73	Mucinous carcinoma	Low	IA	CD-X2 showing strong positivity, CK20 showing strong but patchy positivity and CK7 showing diffuse moderate staining
TM00913	Ovary serous adenocarcinoma	High	AJCC IV	https://tumor.informatics.jax.org/mtbwi/pdxDetails.do?modelID=TM00913

Supplementary Table 2.
List of the antibodies used for western blot analysis

Name	Company	Catalogue number	Antibody dilutions
mTOR	Cell Signaling	2972S	1:1000
p-mTOR (2448)(49F9)	Cell Signaling	2976S	1:1000
p70 S6 Kinase	Cell Signaling	9202	1:1000
phospho-p70 S6 Kinase	Cell Signaling	9205	1:1000
AKT	Cell Signaling	9272S	1:1000
pAKT (S473)	Cell Signaling	1:1000	1:1000
pSTAT3 (Y705)	Cell Signaling	9131S	1:1000
STAT3 79D7	Cell Signaling	1:1000	1:1000
p44/42MAPK (total)	Cell Signaling	#9102	1:1000
Phospho-p44/42MAPK	Cell Signaling	9101	1:1000
S6 ribosomal protein	Cell Signaling	2217	1:1000
p-S6 ribosomal protein	Cell Signaling	4858S	1:1000
LIF	Santa Cruz Biotechnology	sc-515931	1:500
LIFR	Santa Cruz Biotechnology	sc-659	1:500
Cas9	Cell Signaling	14697	1:1000
GPX4	Cell Signaling	52455	1:1000
NRF2	Cell Signaling	12721	1:1000
xCT/SLC7A11	Cell Signaling	12691	1:1000
p27	Santa Cruz Biotechnology	SC-528	1:1000
p21	Cell Signaling	2947P	1:1000
Vinculin	Sigma	V9264-200UL	1:1000
beta-actin	Cell Signaling	8457S	1:1000

Supplementary Table 3.
Primer sequences of the genes used in this manuscript for RT-qPCR

Gene name	Forward primer	Reverse primer
Oct4	GGAGGAAGCTGACAACAATGAAA	GGCCTGCACGAGGGTTT
GAPDH	GGAGCGAGATCCCTCCAAAAT	GGCTGTTGTCATACTTCTCATGG
Beta actin	GTGGGCATGGGTCAGAAG	TCCATCACGATGCCAGTG
SNAI2	CGAACTGGACACACATACAGTG	CTGAGGATCTCTGGTTGTGGT
Nanog	ACAACTGGCCGAAGAATAGCA	GGTTCCCAGTCGGGTTCAC
BMI1	CCACCTGATGTGTGTGCTTTG	TTCAGTAGTGGTCTGGTCTTGT
Vimentin	AGTCCACTGAGTACCGGAGAC	CATTTCACGCATCTGGCGTTC
SNAI1	CCCCAATCGGAAGCCTAACT	GCTGGAAGGTAAACTCTGGATTAGA

Supplementary Table 4.
Monoclonal antibodies to murine antigens and reagents for flow cytometry

Fluorochrome-conjugated monoclonal antibodies (mAbs) to murine (mu) antigens		
α–muCD45-Pacific Blue™	BioLegend	Cat. # 103126 (Clone 30-F11)
α–muCD45-biotin	BioLegend	Cat. # 103104 (Clone 30-F11)
α–muCD3-APC-Cy7	BioLegend	Cat. # 100221 (Clone 17A2)
α–muCD8b-PE	eBioscience	Cat. # 25-0083-82 (Clone H35-17.2)
α–muCD11b-APC	BioLegend	Cat. # 101212 (Clone M1/70)
α–muCD11b-biotin	BioLegend	Cat. # 101204 (Clone M1/70)
α–muB220-APC	BioLegend	Cat. # 103212 (Clone RA3-6B2)
α–muCD80-PE	BD Biosciences	Cat. # 553769 (Clone 16-10A1)
α–muPhosphoSTAT1 (Tyr701)-PE	Cell Signaling	Cat. # 8062 (Clone 58D6)
α–mucMAF-PerCP-eFluor™710	eBioscience	Cat. # 46-9855-42 (Clone sym0F1)
α–muPD-L1-PerCP-eFluor™710	ThermoFisher	Cat. # 46-5983-42 (Clone MIH5)
α–muPD-L1-Brilliant Violet 421™	BioLegend	Cat. # 124315 (Clone 10F.9G2)
α–muLy6G-Pacific Blue™	BioLegend	Cat. # 127612 (Clone 1A8)
α–muGr-1-APC	BioLegend	Cat. # 108412 (Clone RB6-8C5)
Other reagents		
Fixable Viability Dye eFlour™ 780	eBioscience	Cat. # 65-0865-14
Fixable Viability Dye eFlour™ 506	eBioscience	Cat. # 65-0866-14
7-AAD (7-Aminoactinomycin D)	SigmaAldrich	Cat. # A9400
APC-Streptavidin	BioLegend	Cat. # 405207
PE/Cy7-Streptavidin	BioLegend	Cat. # 405206

Fig. 1c uncropped blots

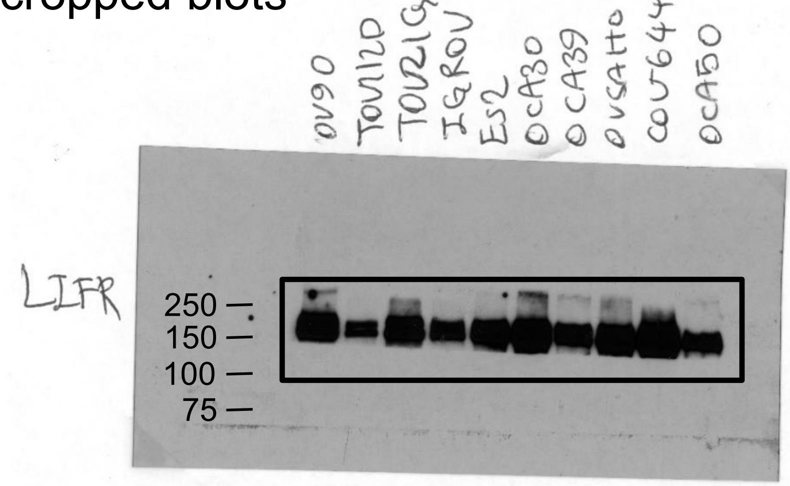

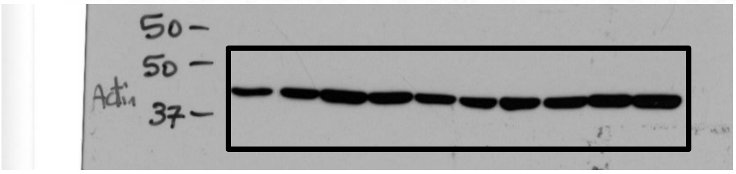

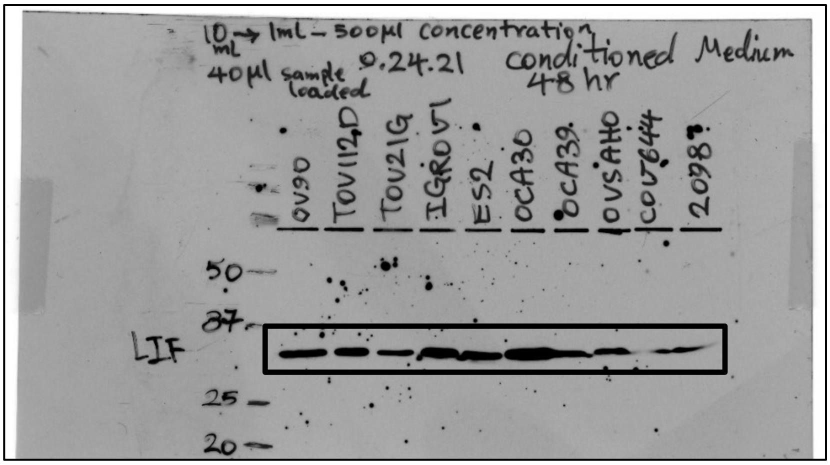

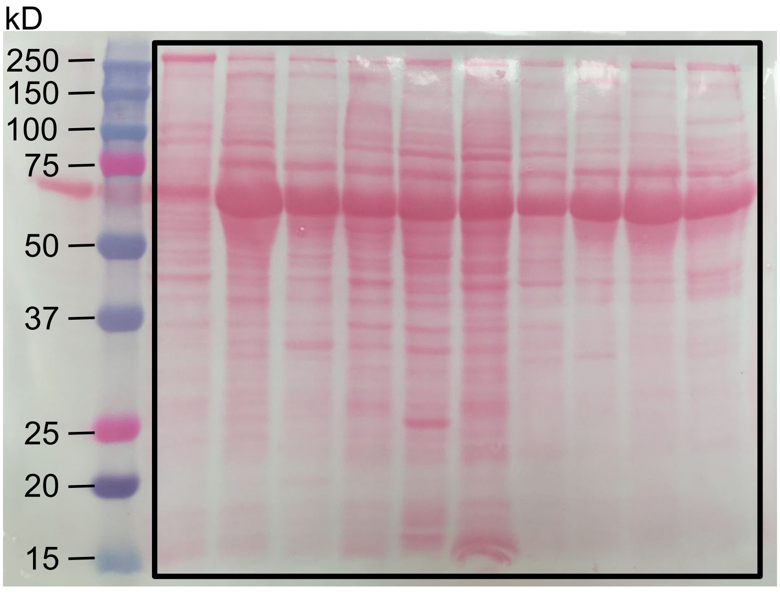

Fig. 1f uncropped blots

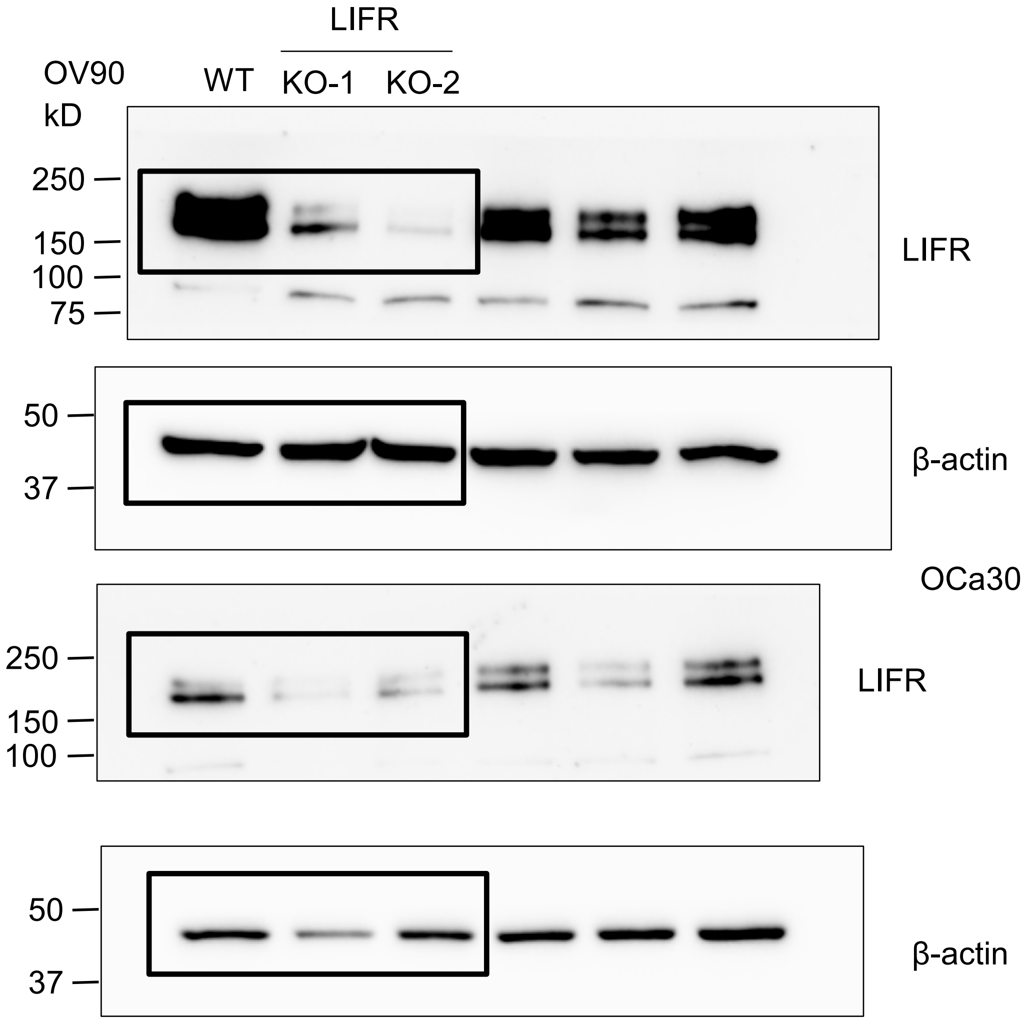

Fig. 1i uncropped blots

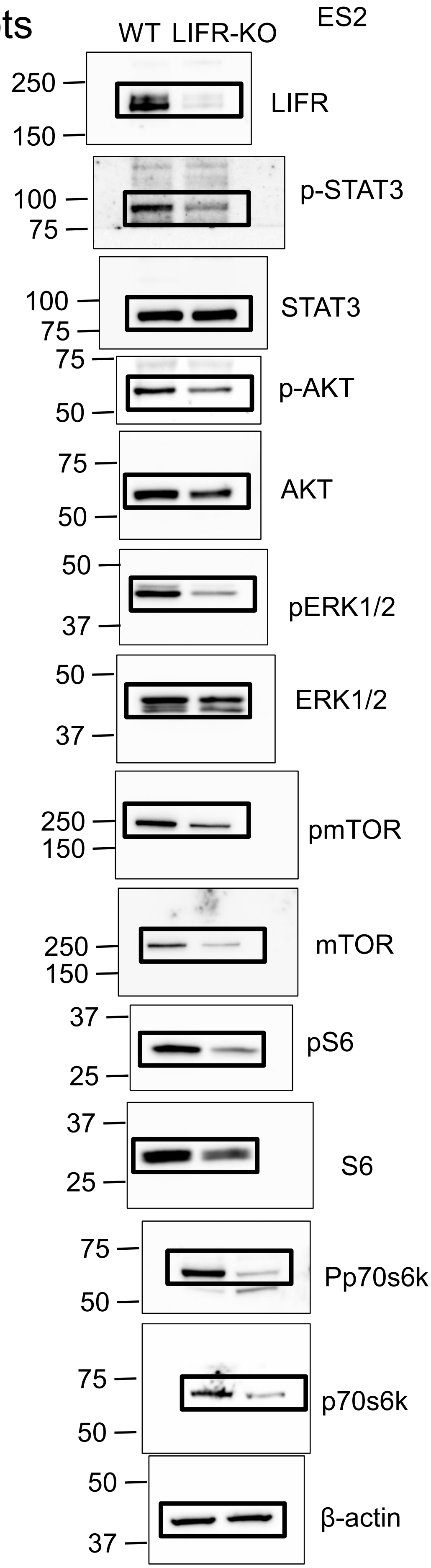

Fig. 2e uncropped blots

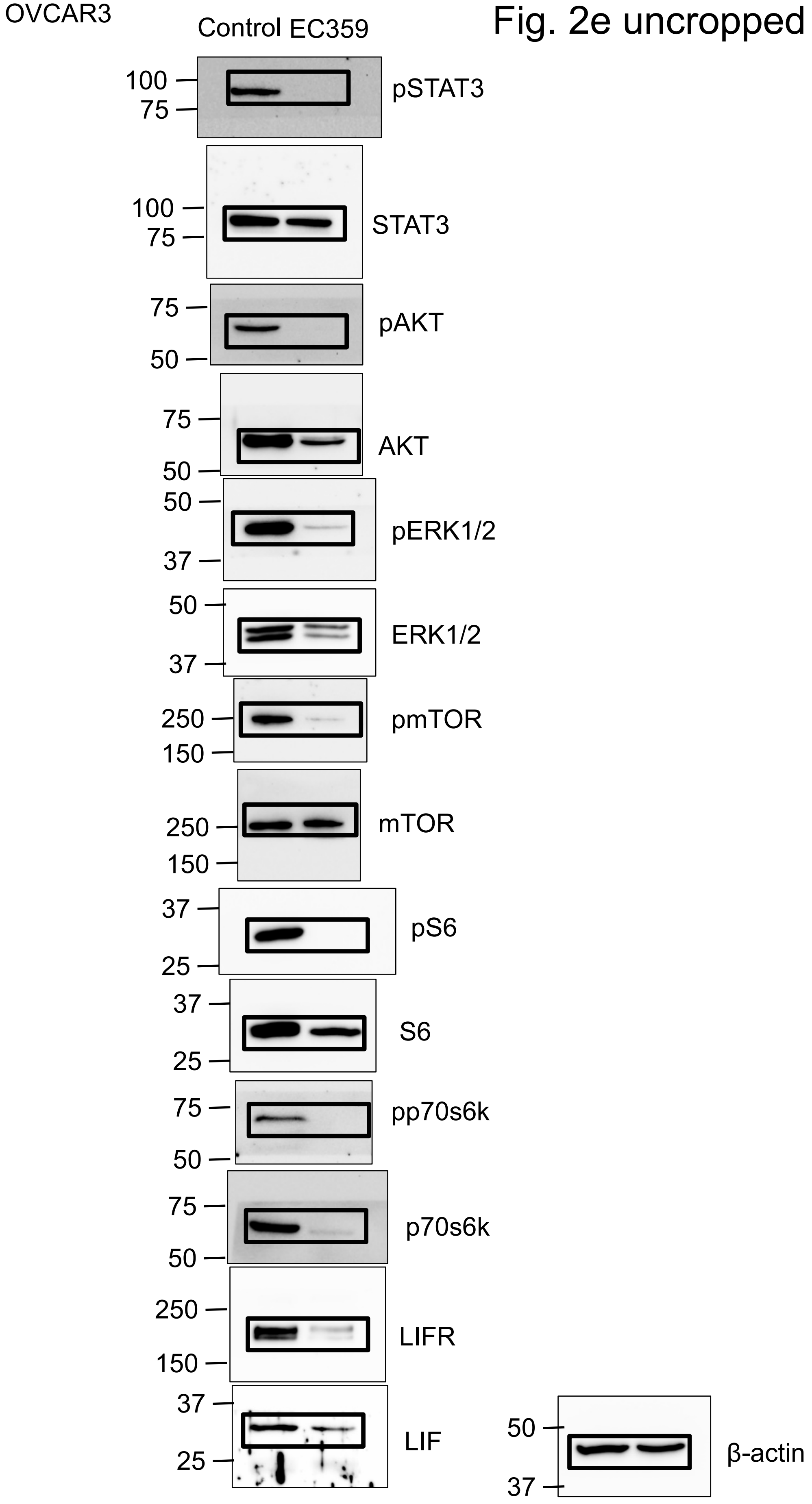

Fig. 2f uncropped blots

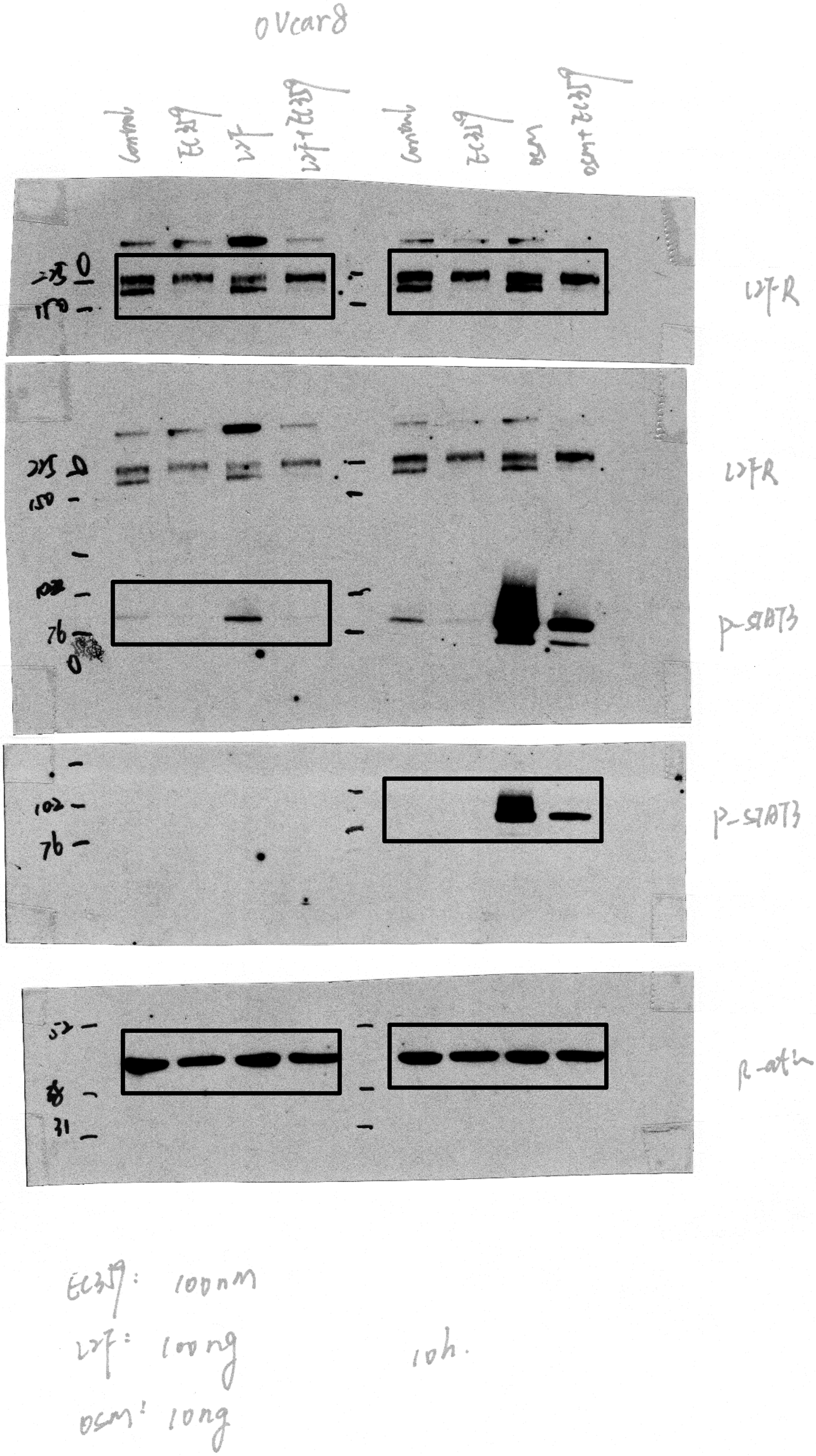

Fig. 2f uncropped blots

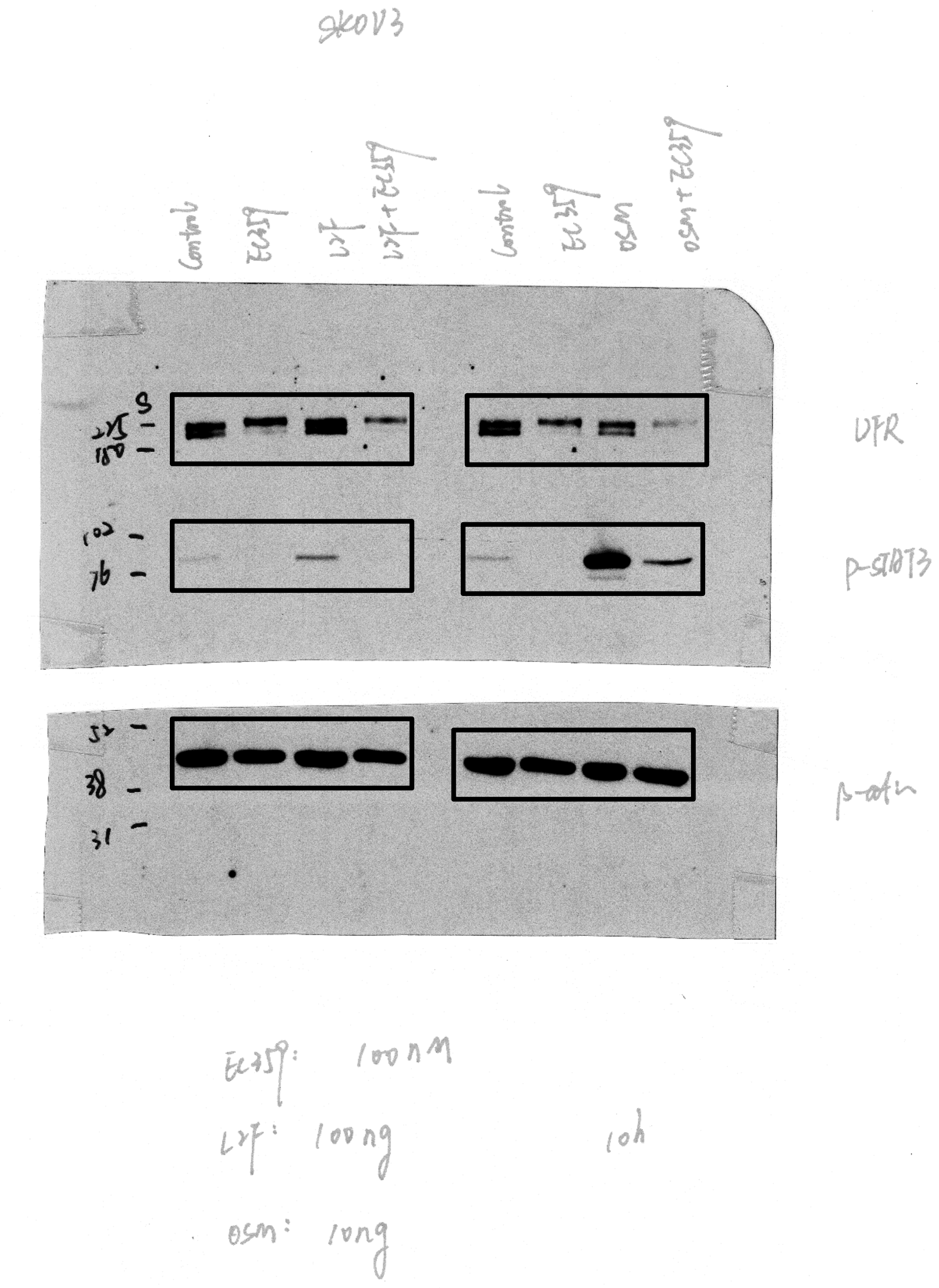

Fig. 2f uncropped blots

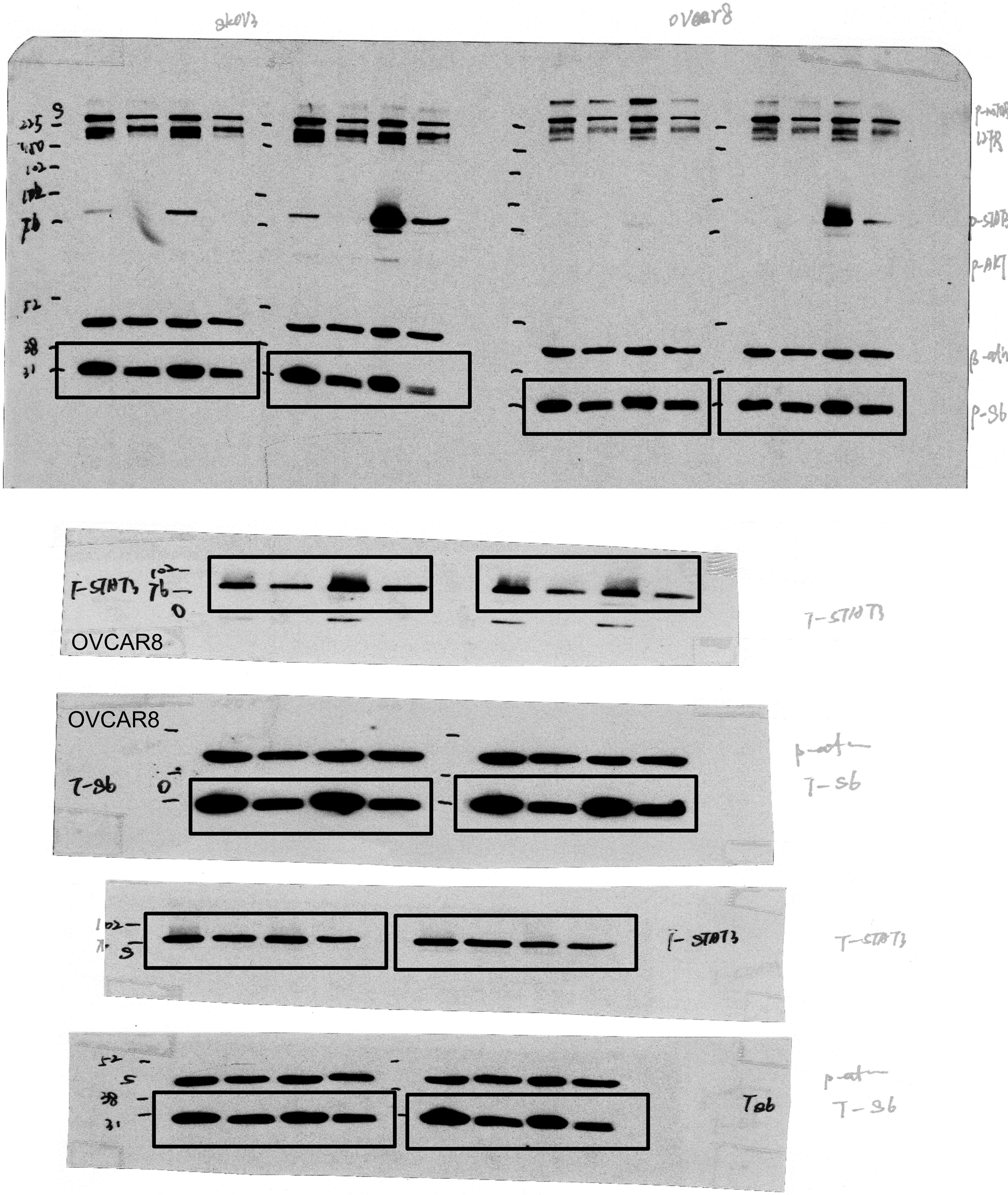

Fig. 3a uncropped blots

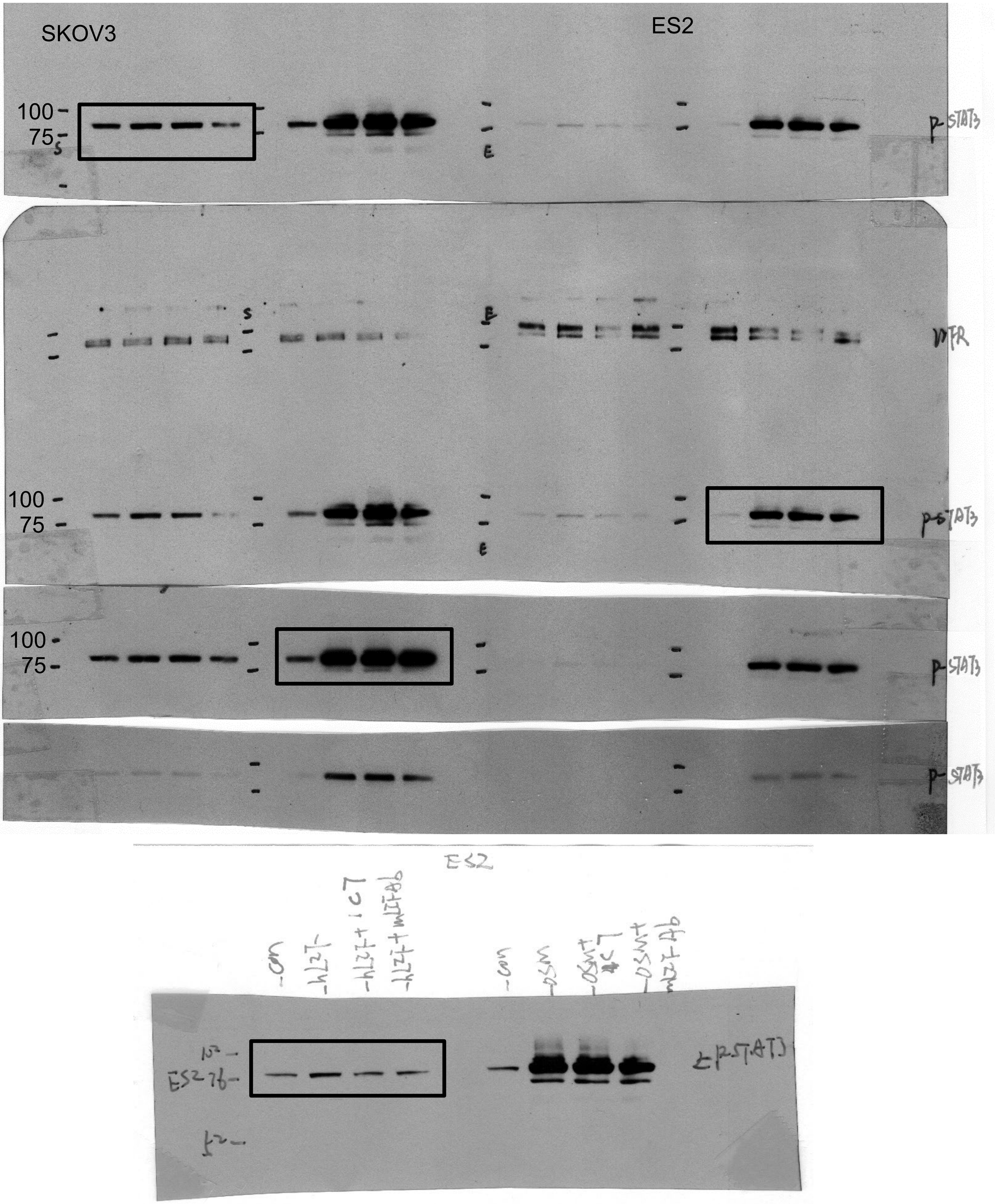

Fig. 3a uncropped blots

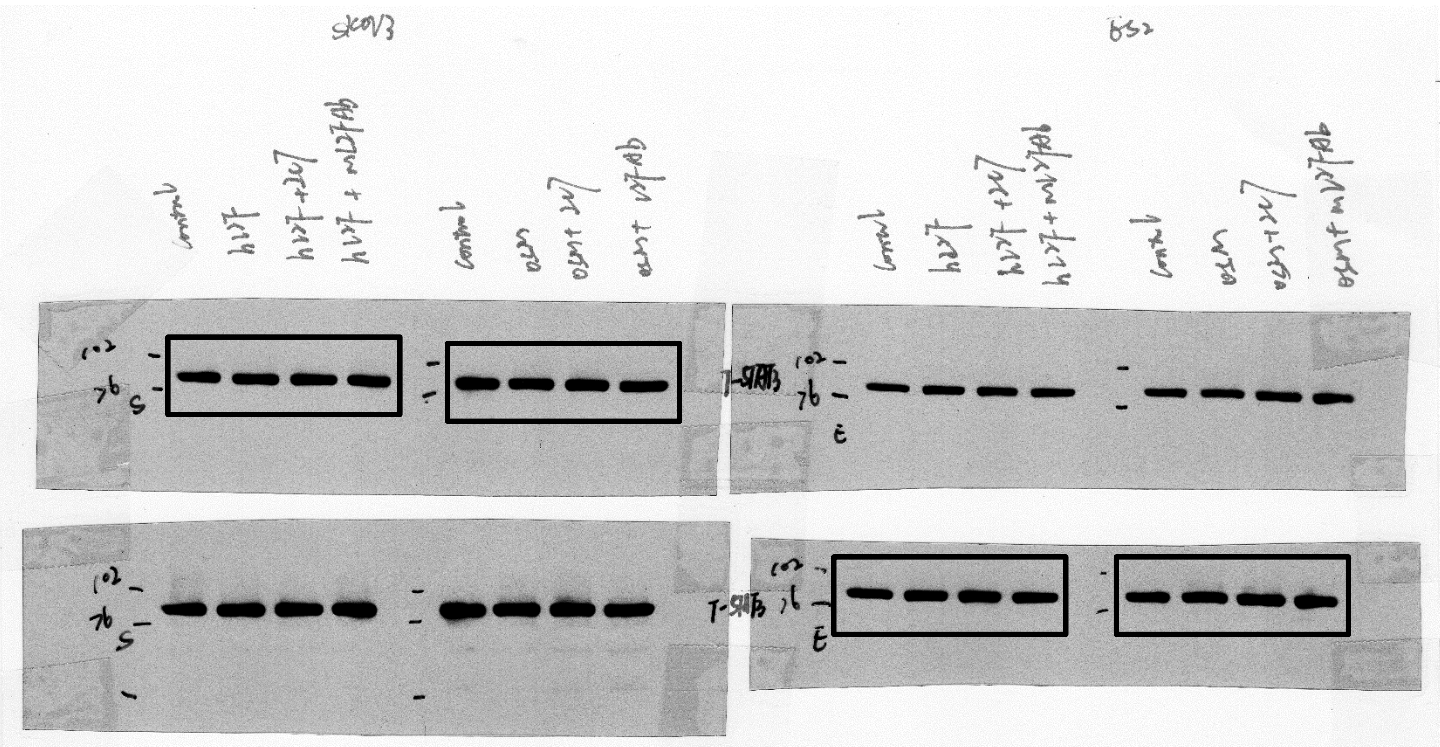

Fig. 1e

Normal (8)	Serous (51)
2	2
3	2
3	3
2	3
1	3
2	2
2	3
2	3
	3
	2
	3
	3
	3
	3
	3
	3
	2
	2
	3
	2
	3
	2
	2
	2
	2
	1
	2
	3
	3
	3
	2
	3
	2
	3
	3
	3
	3
	3
	3
	3
	3
	2
	3
	3
	3
	3
	3
	2
	3
	3
	3

Fig. 1h

	WT			LIFR-KO-1			LIFR-KO-2		
OV90	184	193	189	130	134	124	74	66	60
OCa-30	156	149	155	80	97	73	85	87	79

Fig. 1j

Days after injection	WT					LIFR-KO				
1	1.13E+08	1.51E+08	99100000	2.26E+08	1.92E+08	22600000	6020000	57800000	21200000	44300
4	5.36E+08	3.29E+08	1.27E+08	4.4E+08	1.48E+08	68200000	39300000	585000	18300000	265000
8	17000000	8.3E+08	1.88E+08	6060000	1.11E+09	4.53E+08	3.2E+08	3.63E+08	88200000	27300
12	2.78E+09	7.98E+08	9.39E+08	1.4E+09	7.73E+08	1.11E+09	7.78E+08	6.35E+08	3.17E+08	137000
16	6.99E+09	4.15E+09	3.59E+09	1.64E+09	19900000	3.13E+09	1.82E+09	2.31E+09	9.12E+08	785000
19	1.22E+10	1.38E+10	7.72E+09	2.02E+10	1.19E+10	1.07E+10	7.23E+09	5.47E+09	2.75E+09	1730000
23	3.08E+10	2.87E+10	2.04E+10	4.86E+10	3.48E+10	1.47E+10	1.94E+10	2.55E+10	6.47E+09	8270000
26	6.97E+10	4.68E+10	4.4E+10		5.96E+10	3.08E+10	3.32E+10	3.39E+10	1.61E+10	20300000

Fig. 1k

WT	LIFR-KO
0.738	0.38
0.598	0.392
0.46	0.41
0.412	0.096
	0.15

Fig. 1l

WT	LIFR-KO
66	23
56	32
36	28
49	8
	10

Fig. 2a

Established OCa cells

nM	OVCAR5			OVCAR8			SKOV3			COV644		
0.1	100	100	100	100	100	100	100	100	100	100	100	100
1.5	100.6444	89.15845	95.18575	91.48376	94.29324	95.52239	97.28916	97.13855	96.08434	100.2809	105.7584	108.2865
3.1	103.9992	93.42305	93.59363	87.79631	87.26953	86.04039	99.24699	99.39759	104.6687	104.9157	103.6517	106.1798
6.25	92.34268	82.44882	88.19181	74.10009	74.89025	72.95874	95.93373	99.09639	98.94578	79.21348	67.83708	78.37079
12.5	72.83927	76.36467	76.36467	43.28358	40.64969	37.13784	53.61446	60.24096	56.92771	64.04494	68.67978	71.20787
25	59.70432	59.47688	57.99848	22.65145	24.93415	22.56365	17.46988	22.74096	20.48193	52.66854	47.61236	50.14045
50	42.64594	45.14784	40.82638	17.64706	14.3108	12.81826	10.99398	11.14458	12.3494	37.92135	35.81461	37.5
100	23.65428	23.93859	23.4837	15.71554	14.57419	15.10097	10.69277	9.939759	10.24096	33.70787	38.3427	34.12921

ES2			TOV21G			OV90			ID8		
100	100	100	100	100	100	100	100	100	100	100	100
19.51893	22.47634	18.45426	97.24907	95.46468	93.45725	81.03757	81.84258	86.40429	100	100	100
9.108833	9.463722	10.05521	96.13383	94.34944	98.14126	57.15564	52.32558	53.39893	100	100	100
6.388013	6.506309	6.269716	59.10781	46.6171	50.85502	35.95707	31.12701	24.95528	100	100	100
6.624606	6.624606	6.388013	19.18216	13.829	13.60595	22.27191	19.32021	20.12522	100	100	100
6.269716	6.388013	7.097792	10.92937	11.37546	11.15242	17.17352	16.63685	16.10018	60.17845	52.64375	64.34237
7.216088	7.334385	7.452681	12.26766	12.49071	12.04461	17.7102	17.97853	17.17352	49.96695	44.01851	43.91937
6.624606	6.506309	7.216088	11.59851	11.59851	11.15242	16.36852	15.83184	16.90519	39.75545	36.18638	39.45803

OVCAR3			OVSAHO			IGROV1			TOV112D		
100	100	100	100	100	100	100	100	100	100	100	100
88.43826	85.35109	81.90073	89.94083	92.01183	91.42012	87.28929	86.37813	68.70159	96.54047	103.3943	101.436
41.04116	37.2276	36.86441	78.69822	80.76923	86.68639	49.93166	56.12756	57.03872	98.10705	104.1775	93.60313
18.15981	17.61501	16.88862	66.86391	67.75148	72.48521	26.24146	25.14806	28.61048	89.88251	93.99478	90.6658
17.97821	16.70702	16.16223	27.81065	31.36095	34.61538	14.57859	17.12984	14.39636	60.90078	67.36292	67.55875
15.25424	16.16223	15.79903	17.75148	19.23077	21.59763	12.3918	11.11617	11.48064	22.91123	18.79896	17.23238
13.80145	12.53027	12.34867	20.71006	20.71006	21.59763	12.02733	13.12073	10.75171	14.29504	11.94517	12.33681
11.98547	11.44068	11.80387	17.45562	16.86391	16.56805	12.02733	9.658314	10.02278	11.16188	11.55352	10.18277

Primary OCa cells

nM	OCa27			OCa1			OCa30			OCa39		
0.1	100	100	100	100	100	100	100	100	100	100	100	100
1.5625	98.06378	97.03872	90.88838	100	100	100	98.63014	100.0428	98.37329	100.6552	102.1464	90.82693
3.125	75.85421	88.49658	91.57175	114.7548	102.7292	105.9275	78.08219	77.69692	84.24658	92.25034	102.8242	91.23362
6.25	42.36902	43.05239	48.17768	90.1919	75.09595	68.0597	16.18151	19.7774	21.31849	80.65974	77.94849	84.45549
12.5	33.48519	32.80182	28.70159	48.61407	46.3113	52.32409	10.14555	10.53082	10.27397	33.3484	30.90827	35.8563
25	25.62642	24.94305	26.30979	12.79318	14.58422	20.72495	8.476027	8.604452	8.604452	7.659286	5.964754	9.624944
50	23.23462	19.81777	19.81777	8.187633	8.443497	7.93177	7.705479	7.833904	8.090753	4.74469	5.354722	5.964754
100	17.76765	18.10934	18.10934	10.23454	10.49041	10.36247	6.421233	5.650685	6.035959	4.066878	4.20244	3.931315

OCa73			OCa66		
100	100	100	100	100	100
95.88785	87.33645	92.38318	91.45768	91.22257	96.63009
90.28037	75.14019	86.63551	90.04702	82.99373	93.80878
54.53271	37.85047	48.08411	63.0094	64.65517	67.47649
23.13084	19.48598	20.04673	36.2069	37.38245	34.32602
10.51402	9.252336	9.11215	28.9185	23.04075	21.15987
9.53271	9.11215	9.11215	18.33856	18.33856	18.33856
7.570093	7.850467	7.71028	16.45768	16.69279	17.39812

Fig. 2a

Primary OCa Ascites

nM	AS20			AS21			AS23			AS25		
0.1	100	100	100	100	100	100	100	100	100	100	100	100
1.5625	85.71429	82.31707	82.18641	77.22372	73.58491	67.52022	91.03194	91.76904	85.31941	88.92385	106.8597	120.8307
3.125	88.58885	75.78397	78.65854	60.24259	55.79515	77.62803	99.87715	92.32187	77.57985	103.8389	104.9717	92.69981
6.25	80.61847	71.60279	74.47735	42.45283	42.04852	44.47439	67.44472	67.81327	66.89189	69.85525	80.80554	83.25991
12.5	62.06446	63.24042	59.84321	32.74933	33.55795	33.15364	57.49386	52.33415	52.33415	50.97546	53.61863	56.2618
25	48.08362	47.95296	49.91289	32.34501	33.15364	32.34501	25.61425	24.69287	23.7715	25.11013	25.29893	28.5085
50	25.21777	26.91638	25.08711	27.49326	26.28032	27.08895	16.21622	16.21622	16.76904	16.61422	18.12461	16.61422
100	15.28746	14.1115	13.45819	24.66307	24.66307	23.85445	12.71499	12.34644	13.08354	13.02706	13.97105	14.72624

AS28			AS29		
100	100	100	100	100	100
89.56715	85.90455	75.91565	88.17829	83.72093	87.5969
76.58158	77.91343	54.93896	81.97674	86.43411	88.95349
53.94007	51.94229	34.96115	89.92248	87.0155	74.22481
47.94673	35.29412	30.29967	47.09302	41.66667	42.44186
30.96559	26.30411	26.30411	41.08527	47.28682	31.78295
22.64151	19.9778	20.97669	14.53488	14.53488	16.47287
20.31077	22.30855	20.64373	13.37209	11.62791	13.17829

Fig. 2c

ES2			
contrl	86	78	76
5nM	41	52	52
10nM	19	20	21
20nM	0	2	2

OV90			
contrl	156	195	174
5nM	36	39	43
10nM	12	13	15
20nM	7	4	5

OCa30			
contrl	99	93	84
10nM	96	89	84
20nM	50	58	41
40nM	9	10	9

OCa39			
contrl	116	103	120
10nM	81	91	74
20nM	34	35	38
40nM	9	8	10

Fig. 2d

EC359 (nM)

	0			10			25			50		
OVCAR3/STAT3/Luc	69666.26	77939.55	88070.73	5970.444	3488.735	4496.394	823.456	695.5416	784.4885	1153.812	278.9854	729.0542

	0			10			25			50		
OVSAHO/STAT3/Luc	60582.31	50556.64	42620.51	32869.2	28519.41	26804.03	2764.575	2085.758	2465.07	415.9735	498.3395	385.8945

	0			10			25			50		
OCa30-STAT3-LUC	119085.2	119908	122836.4	81577.97	89426.97	103356	31586.81	21169.08	14043.25	2112.977	1354.213	1441.588

Fig. 3b

SKOV3-STAT3-Luc				
Control	LIF	LIFR ab	LIF ab	EC359
6547.934	11442.92	5999.927	5291.284	175.4715
6326.705	10225.08	5918.958	6032.885	162.977
7400.431	12685.44		6731.497	239.0361

SKOV3-STAT3-Luc				
Control	OSM	LIFR ab	LIF ab	EC359
6662.965	84233.26	56125.7	85481.97	377.0807
7054.506	79936.58	75187.62	77719	231.9196
4502.131	94206.82	63851.21	81824.37	214.4082

Fig. 3c

STAT3i

uM	ES2			OVCAR8			SKOV3		
0.1	100	100	100	100	100	100	100	100	100
1.5	96.86051	102.1049	106.0649	105.5744	100.0485	102.8114	108.4703	113.9697	106.3843
3.125	101.6768	101.0346	103.8173	107.6103	103.8294	101.0664	115.2971	107.7118	104.6776
6.25	94.61291	99.42918	99.1081	101.0664	98.0126	96.84925	101.4539	102.402	104.2984
12.5	90.33179	101.7838	100.3924	104.8473	99.17596	97.28551	102.5917	113.0215	106.574
25	92.15127	98.89404	100.0714	95.54048	99.75763	96.55841	98.04046	100.3161	89.31732
50	95.04103	93.64966	97.82376	72.27339	73.00048	72.56423	59.92415	62.19975	59.35525
100	80.59222	78.13057	81.34142	36.2094	34.46437	35.04605	32.61694	36.97851	37.16814

Fig. 3d

JACi

uM	ES2			OVCAR8			SKOV3		
0.1	100	100	100	100	100	100	100	100	100
0.3	97.95918	105.3061	104.8609	99.34241	103.2879	100.7515	102.4744	98.3102	100.4828
0.625	102.3006	103.1911	102.5232	99.76515	96.10146	98.91968	105.1901	103.0175	98.12915
1.25	99.74026	97.73655	100.6308	94.97417	102.7243	95.53781	102.2933	99.7586	99.3965
2.5	97.4026	101.8553	99.18367	95.81963	90.04227	100.6106	104.828	101.7502	100.8449
5	98.51577	101.6327	102.4119	72.14655	102.3016	103.147	86.90404	93.6029	94.14605
10	93.39518	97.17996	101.744	56.5054	63.69187	69.18741	82.19674	86.36089	87.08509
20	70.3525	75.58442	81.81818	42.6961	55.94176	51.57351	63.91068	68.43693	67.35063

Fig. 3e

	Control			STAT3i(20μM)			Ruxolitinib(10μM)		
Control	100	100	100						
STAT3i				110.0794	109.4836	104.1212			
Ruxolitinib							88.05369	94.09396	92.34899
EC359									

	EC359(6nM)			STAT3i+EC359			Ruxolitinib+EC359		
Control									
STAT3i									
Ruxolitinib									
EC359	74.52454	68.53722	73.67927	36.34558	42.75074	43.49553	34.22819	35.43624	36.37584

Fig. 3g

ES2-CSCs spheres

0	500	1000
536.61	321.315	108.81
477.555	374.79	112.065
580.32	345.96	114.39
389.67	274.35	90.21
577.065	314.34	100.44
567.3	264.585	106.02
436.635	272.025	102.765
568.23	349.215	112.995
488.715	427.335	81.375
515.22	345.96	110.205

Fig. 3h

ES2-CSCs spheres

0	500	1000
5	5	0
6	3	1
4	2	0
12	5	1
5	3	4
9	5	3
6	5	1
5	2	2
4	3	2
3	2	0
7	4	1
5	3	0

Fig. 3i

nM	SKOV3			OV90			ES2			IGOVR1		
1	100	100	100	100	100	100	100	100	100	100	100	100
15.625	99.44853	96.72564	96.20864	102.4578	102.4792	92.62663	46.59631	51.15614	76.29481	100	100	100
31.25	91.93474	95.45037	98.27665	98.63219	84.50524	89.72003	15.91646	19.37626	28.19261	100	100	100
62.5	92.04963	91.17647	90.65947	87.04851	104.7446	94.44326	6.873381	13.30429	11.64426	98.90158	93.89813	92.9492
125	83.31801	85.78814	90.16544	98.95277	99.03826	99.50844	2.360295	2.427253	2.763937	23.18351	33.80384	25.85152
250	81.00873	81.59467	82.96186	104.0821	87.68968	95.36226	1.255345	1.519105	1.519105	15.17875	15.42744	15.57712
500	74.62086	74.26471	68.55469	53.17376	47.51015	53.6012	1.564943	1.377659	1.821316	10.73784	10.39791	11.81808
1000	26.84972	27.59651	25.26425	21.16905	15.00107	13.55418	0.981878	1.018245	1.176501	9.966674	10.25073	8.681401

Fig. 3j

	OCa30			OCa39		
Control	1	1	1	1	1	1
EC359	0.088889	0.066667	0.088889	0.071111	0.066667	0.057778

Fig. 3k

foldchange

WT	LIFR-KO-1	LIFR-KO-2
1	0.409091	0.272727
1	0.409091	0.136364
1	0.409091	0.272727

Fig. 3l

ES2 ELDA

Dose	Tested	Response	Group
1	16	12	Control
5	16	16	Control
10	16	16	Control
20	16	16	Control
50	16	16	Control
1	16	2	EC359
5	16	5	EC359
10	16	10	EC359
20	16	16	EC359
50	16	16	EC359

SKOV3 ELDA			
Dose	Tested	Response	Group
1	16	11	Control
5	16	12	Control
10	16	16	Control
20	16	16	Control
50	16	16	Control
1	16	6	EC359
5	16	6	EC359
10	16	13	EC359
20	16	16	EC359
50	16	16	EC359

Fig. 3m

ES2-CSCs

	Control			EC359		
Oct-4	1	1	1	0.656837	0.624784	0.602423
Nanog	1	1	1	0.890956	0.695959	0.636621
BMI	1	1	1	0.65805	0.676632	0.710129
SNAI2	1	1	1	0.532514	0.572209	0.507324
Vimentin	1	1	1	0.787338	0.817051	0.84713
SNAI1	1	1	1	0.655213	0.673146	0.764826

Fig. 3n

OV90-CSCs	Control			EC359		
Oct-4	1	1	1	0.658486	0.719797	0.739556
Nanog	1	1	1	0.743128	0.859563	0.669996
BMI	1	1	1	0.81641	0.825273	0.786792
SNAI2	1	1	1	0.751204	0.794982	0.778501
Vimentin	1	1	1	0.69533	0.727441	0.646109
SNAI1	1	1	1	0.782245	1.021521	0.796206

Fig. 4a

Pathways	-log (p value)	Z-score
Oxidative Phosphorylation	9.39	-4.667
Glycolysis I	5.25	-3.464
Gluconeogenesis I	2.95	-3
EIF2 Signaling	11.5	-2.466
Superpathway of Cholesterol Biosynthesis	2.1	-2.121
Pyrimidine Ribonucleotides Interconversion	0.967	-2.121
Pyrimidine Ribonucleotides De Novo Biosynthesis	0.885	-2.121
Glutathione-mediated Detoxification	0.36	-2
Phospholipase C Signaling	1.01	-1.671
VEGF Signaling	1.06	-1.604
Mitotic Roles of Polo-Like Kinase	5.46	-1.508
PI3K/AKT Signaling	1.32	-1.414
CXCR4 Signaling	1.72	-1.225
SAPK/JNK Signaling	1.52	-1.213
Integrin Signaling	3.78	-1.121
NRF2-mediated Oxidative Stress Response	8.53	-1.095
AMPK Signaling	0.742	-1.091
Cell Cycle: G1/S Checkpoint Regulation	2.33	-0.905
Ovarian Cancer Signaling	1.22	-0.832
Hypoxia Signaling in the Cardiovascular System	6.2	-0.707
Sirtuin Signaling Pathway	7.57	2.967
LPS/IL-1 Mediated Inhibition of RXR Function	0	2.887
ATM Signaling	7.1	2.711
HIPPO signaling	1.34	2.53
HMGB1 Signaling	1.8	1.877
Role of BRCA1 in DNA Damage Response	9.46	1.789
Production of Nitric Oxide and Reactive Oxygen Species in Macrophages	0	1.789
Aryl Hydrocarbon Receptor Signaling	3.3	1.633
iNOS Signaling	0.967	2.449
Chemokine Signaling	1.11	1.155

Fig. 4b

Ferroptosis inducing genes

	C1	C2	EC359-1	EC359-2
ATF3	0.054911	-0.05708	0.757947	0.909868
CHAC1	-0.20906	0.182567	0.576223	0.653117
EPAS1	0.085255	-0.09061	0.445028	0.468852
SLC7A11	0.027332	-0.02786	0.416002	0.445841
MIB2	-0.08991	0.084635	0.150723	0.484213
ULK2	-0.06035	0.057927	0.354252	0.235368
KLF2	-0.04186	0.040681	0.134079	0.413143
EGR1	-0.0156	0.015434	0.215239	0.320869
PPARG	0.040842	-0.04203	0.310344	0.210876
TGFB1	-0.11027	0.102435	0.208948	0.308264
NR1D2	0.030197	-0.03084	0.198099	0.201888
TIMM9	0.012869	-0.01299	0.199064	0.18481
PAQR3	0.056446	-0.05875	0.143487	0.139164
FTH1	0.0509	-0.05276	0.059383	0.214298
ACO1	-0.00944	0.009379	0.128612	0.129923
ATG3	0.009073	-0.00913	0.139022	0.094039
AGPS	0.033369	-0.03416	0.118092	0.092151

Ferroptosis repressing genes

	C1	C2	EC359-1	EC359-2
PRDX6	-0.00144	0.00144	-0.09948	-0.12333
SRC	-0.07337	0.069816	-0.11869	-0.1172
NEAT1	0.050274	-0.05209	-0.1243	-0.13737
VCP	0.015434	-0.0156	-0.1226	-0.13972
HSPA5	0.005457	-0.00548	-0.12972	-0.15218
PARP4	0.01611	-0.01629	-0.14478	-0.13892
TXN	0.075039	-0.07916	-0.06697	-0.23074
CAMKK2	-0.06582	0.062945	-0.10967	-0.19087
TMSB4X	0.020488	-0.02078	-0.15011	-0.14968
PARK7	-0.00918	0.009117	-0.18464	-0.14627
P4HB	-0.02724	0.026736	-0.20382	-0.14388
PLIN2	0.051517	-0.05342	-0.20084	-0.15044
ATF2	-0.04255	0.04133	-0.25823	-0.11255
PRDX1	0.013935	-0.01407	-0.17232	-0.19762
CAV1	0.013969	-0.01411	-0.17099	-0.20639
NT5DC2	-0.02404	0.023645	-0.20334	-0.19793
TP53	0.001872	-0.00187	-0.21745	-0.19328
AHCY	0.004377	-0.00439	-0.19427	-0.22101
ATF4	-0.0318	0.031111	-0.20585	-0.21255
TFRC	0.038207	-0.03925	-0.21342	-0.26205
SCD	0.018811	-0.01906	-0.25874	-0.29583
ETV4	-0.06791	0.06486	-0.35323	-0.2327
KIF20A	-0.0136	0.013469	-0.36578	-0.43342
CA9	-0.09445	0.088646	-1.20384	-1.08972

Fig. 4c

ES2	
Control	EC359
0.971831	10.2993
1.088028	10.2993
0.940141	10.28873

OVSAHO	
Control	EC359
1.034483	11.27586
0.965517	10.75862
1.034483	11

OCa30	
Control	EC359
0.969231	1.938462
0.969231	1.892308
1.061538	2.169231

Fig. 4d

OVCAR3									
	EC359			EC359+Fer1			EC359+ZVAD		
0.1	100	100	100	100	100	100	100	100	100
1.56	88.43826	85.35109	81.90073	101.6949	100.4237	95.15738	75	85.35109	76.99758
3.125	41.04116	37.2276	36.86441	97.15496	96.24697	97.15496	61.01695	56.6586	61.01695
6.25	18.15981	17.61501	16.88862	95.33898	92.07022	93.523	20.88378	20.33898	20.33898
12.5	17.97821	16.70702	16.16223	93.523	98.24455	100.2421	17.25182	17.25182	16.16223
25	15.25424	16.16223	15.79903	88.98305	89.52785	91.52542	13.25666	12.34867	12.16707
50	13.80145	12.53027	12.34867	83.53511	81.71913	80.62954	11.98547	12.34867	11.80387
100	11.98547	11.44068	11.80387	78.45036	75.90799	79.53995	11.25908	11.44068	11.25908

OVCAR8	EC359			EC359+Fer1			EC359+ZVAD		
0.1	100	100	100	100	100	100	100	100	100
1.56	97.49227	97.08004	100.7901	87.08348	91.10271	88.11405	66.78117	64.41086	68.73926
3.125	83.78564	91.82412	90.8966	82.44589	83.37341	82.44589	71.3157	71.72793	71.3157
6.25	55.13569	50.08588	59.56716	78.11749	79.86946	77.08691	47.50945	53.69289	54.31123
12.5	21.22982	22.05428	25.45517	79.97252	86.15596	88.32016	29.06218	28.75301	32.1539
25	20.71453	20.40536	19.16867	82.96118	83.78564	79.35417	21.64205	23.39402	20.71453
50	16.38612	14.63415	17.51975	75.54105	79.25112	79.56029	14.11886	15.25249	15.35555
100	9.584335	10.51185	10.71797	70.07901	68.53315	72.55239	9.79045	10.20268	10.71797

Fig. 4e

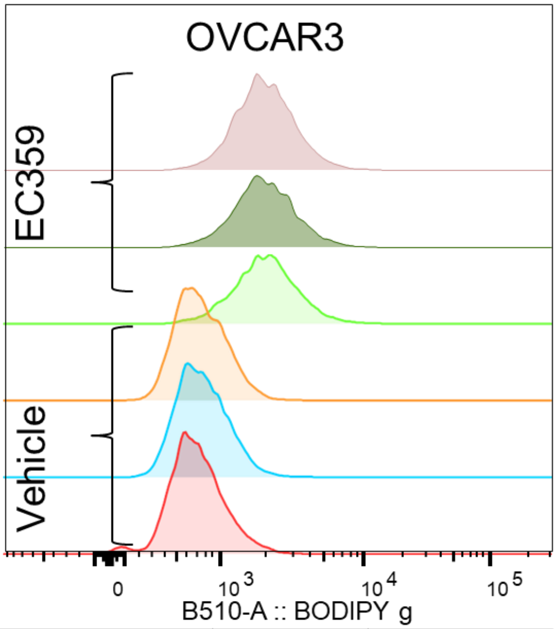

	SampleID	Count	Mean : B510-A
	6	7084	2226
	5	5528	2238
	4	5471	2296
	3	7620	813
	2	7658	836
	1	7893	773

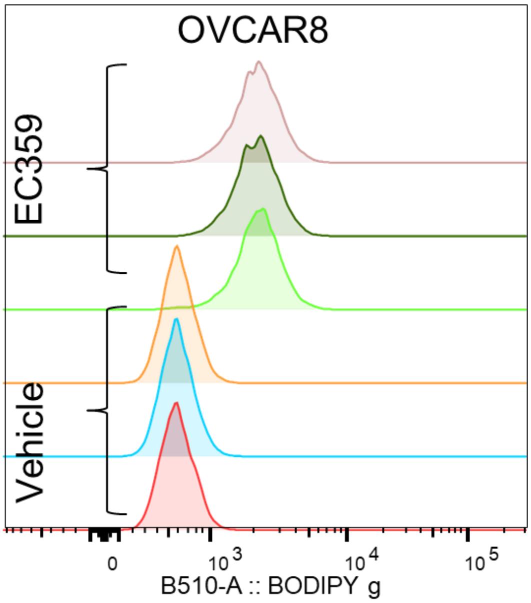

	SampleID	Count	Mean : B510-A
	12	9463	2265
	11	9455	2223
	10	9375	2290
	9	9532	626
	8	9564	616
	7	9083	612

Fig. 4f

SKOV3

EC359 0nM			EC359 12.5nM			EC359 12.5+NRF2-act4			EC359 25nM			EC359 25nM+NRF2-act4		
100	100	100	63.95112	58.85947	53.76782	87.04918	80.40984	75	25.05092	22.60692	23.21792	45.7377	52.13115	44.7541

OVCA
R8

EC359 0nM			EC359 12.5nM			EC359 12.5+NRF2-act4			EC359 25nM			EC359 25nM+NRF2-act4		
100	100	100	43.7947	39.75599	40.89188	76.23604	74.00319	75.11962	23.47497	24.86327	22.71771	49.2823	49.12281	48.48485

Fig. 4g
OVCAR8

	EC359			EC359+3mM NAC			EC359+20μM 2ME		
0.1	100	100	100	100	100	100	100	100	100
1.5	96.76938	93.19085	91.99801	88.06384	89.31298	83.90007	95.51375	90.44863	93.34298
3.1	68.88668	78.13121	73.80716	80.36086	81.19362	79.73629	95.22431	95.07959	91.46165
6.25	35.93439	43.38966	42.04771	65.37127	63.08119	63.70576	88.13314	90.30391	83.50217
12.5	22.51491	24.60239	23.26044	53.7127	46.84247	51.42262	82.19971	83.35745	81.3314
25	14.16501	13.56859	12.37575	39.55586	36.22484	36.22484	76.411	78.00289	76.98987
50	10.73559	11.0338	11.92843	29.97918	29.14643	29.35461	59.04486	62.22865	59.47902
100	9.691849	9.244533	8.946322	21.65163	21.85982	22.90076	32.99566	33.42981	34.44284

OVCAR3	EC359			EC359+3mM NAC			EC359+20μM 2ME		
0.1	100	100	100	100	100	100	100	100	100
1.5	44.0407	45.34884	45.56686	86.51575	83.85827	85.9252	111.3325	109.6513	110.2117
3.1	29.65116	25.50872	27.03488	66.43701	62.8937	74.11417	112.2665	104.9813	110.9589
6.25	15.47965	19.18605	17.87791	49.01575	47.24409	47.53937	96.38854	94.14695	92.09215
12.5	18.09593	15.47965	17.87791	35.1378	34.84252	33.07087	91.15816	96.94894	95.82814
25	12.64535	12.86337	13.0814	31.00394	27.46063	23.0315	70.98381	76.7746	69.67621
50	12.86337	12.64535	12.86337	20.96457	25.3937	24.2126	43.89788	40.90909	35.6787
100	10.68314	11.11919	10.68314	18.89764	20.37402	19.48819	16.43836	14.19676	13.44956

Fig. 4i

Time (minutes)	OVCAR3				OVCAR3 + 100 nM EC359, 1h			
1.552233	49.95907	51.48759	54.00101	101.5438	48.99089	50.51266	42.00249	38.36445
8.42959	53.30357	53.71542	55.49913	102.4945	51.11763	51.98449	43.0282	40.06932
15.299504	53.21756	53.65134	54.73805	101.8285	50.63203	51.37233	42.55355	40.00446
22.270543	70.32052	73.69083	76.46972	129.0334	67.61586	68.68661	56.14759	54.56625
29.141571	71.2927	73.51859	76.57233	128.2174	66.73875	68.75519	57.07141	54.79096
36.00928	70.73981	72.53226	75.59635	126.754	66.36113	67.90137	56.44419	53.63534
42.96697	45.43658	48.18773	49.16836	93.87119	44.44392	46.3234	37.00624	35.72282
49.837266	43.85775	46.90047	44.82324	81.64827	41.19286	41.59807	33.40878	32.30905
56.703495	41.82192	45.69405	43.799	78.29951	41.52127	40.67045	32.29172	31.29804
63.663413	47.79342	48.89462	49.13715	85.88123	43.46305	44.03206	35.55901	34.20915
70.542216	49.02975	49.96054	50.20022	84.32201	43.29117	43.9752	35.37271	33.49081
77.431775	51.19552	52.29756	51.58372	84.38212	45.549	45.95886	36.50396	34.52545

Time (minutes)	OVCAR3 + 100 nM EC359, 3h				OVCAR3 + 100 nM EC359, 12h			
1.552233	30.56164	33.9003	30.70439	31.20577	15.91928	17.83995	13.00121	14.00099
8.42959	33.24155	36.10752	33.55635	32.48504	17.46457	19.36352	13.59887	15.12297
15.299504	33.6775	36.81483	33.76301	32.53803	17.46678	19.56656	13.94044	15.34304
22.270543	42.34614	48.73957	47.74982	46.47517	12.64085	15.04638	9.017039	11.04165
29.141571	43.81994	51.48022	49.70405	47.04416	12.83089	15.29436	9.424519	10.75203
36.00928	45.07888	51.8679	50.16719	47.20624	13.47655	15.17705	10.29876	11.11889
42.96697	47.05514	52.37848	50.09091	46.66561	11.87645	13.81432	10.19821	10.48592
49.837266	45.40534	51.06571	47.35926	43.85803	11.82106	13.55071	9.299404	9.678113
56.703495	45.27636	50.47603	46.55659	42.7566	11.99482	13.37277	9.118498	9.239941
63.663413	44.30511	49.67743	47.28939	43.64366	11.52933	13.30978	9.15512	9.819807
70.542216	46.55839	51.21866	47.21066	44.30512	12.63231	13.61987	8.996397	9.85152
77.431775	47.94101	51.9151	48.38057	44.51161	13.17141	14.37011	9.704965	10.19626

Fig. 5a

Days after treatment	Vehicle						EC359					
1	93.15	160.55	104.28	153	124.03	143.65	167.21	93.64	120.09	112.27	116.06	99.24
3	138.75	208.09	198.58	309.02	224.26	267.19	115.2	154.21	119.42	75.43	126	164.15
8	282.534	446.9275	306.128	646.07	416.25	668.54	250.488	249.953	157.696	96.77	304	275.63
11	502.51	784.15	488.2	817.22	560.95	836.37	231.23	401.54	226.15	160.2	274.6	328.54
21	1196.42	1558.2	1245.18	1168.35	1384.21	1508.3	365.04	455.99	440.9	209.2	402.19	419.9

Fig. 5b

Vehicle	EC359
0.664	0.439
0.765	0.664
0.574	0.463
0.776	0.42
0.843	0.675
0.81	0.584

Fig. 5c

Days after treatment	Vehicle					EC359			
1	19.8	18.8	17.9	20.3	20.1	19	17.9	18.2	17.8
3	19.8	18.8	17.9	20.3	20.1	19	17.9	18.2	17.8
8	19.6	19.3	17.6	20.4	20.3	19.4	17.4	16.9	17.2
11	18.4	18.3	17.1	19.4	19.1	19	17.5	16	16.1
21	16	17	16	18	17	20	17	16	15

Fig. 5d

Days after treatment	Vehicle						
0	313.45	243.09	316.37	246.05	191.1	368.55	119.42
3	664.85	694.01	526.9	441.1	589.84	541.02	414.74
7	1533.68	1696.31	884.17	753.77	820.82	1534.83	1534.83
11	1752.19	2599.05	1425.41	1291.3	1556.07	2899.71	1437.5

Days after treatment	EC359						
0	0	323.78	271.48	215.14	265.72	215.14	211.72
3	0	533.02	291.96	422.05	264.38	382.23	275.2
7	357.64	631.38	553.76	789.57	552.25	822.02	786.89
11	787.18	1316.97	907.2	1128.96	960.76	1620.43	1115.4

Fig. 5e

Vehicle	EC359
1.38	0.743
1.373	0.651
0.79	0.749
1.76	0.48
1.67	0.81
1.09	0.55
0.84	0.58

Fig. 5f

Days after treatment	Vehicle				EC359			
0	18	18	18	19	18	17	16	16
3	18	18	20	19	18	17	16	16
7	18	19	20	20	18	16	17	16
10	19	20	21	20	19	18	18	17

Fig. 5g

Days after treatment	Vehicle					
1	100	100	100	100	100	100
2	128	171	488	556	85.9	144
5	442	341	409	494	157	449
13	274	1320	522	1000	525	1000
19	799	2160	1350	1380	594	539

Days after treatment	EC359					
1	100	100	100	100	100	100
2	38.9	46.3	97.4	34.7	60.5	203
5	102	110	187	171	145	235
13	160	173	143	200	358	90.3
19	192	361	208	231	433	355

Fig. 5h

Vehicle	EC359
1.08	0.902
1.345	0.684
1.302	0.816
0.898	0.859
1.211	0.82
1.524	0.554

Fig. 5 i

Days after treatment	Vehicle						EC359					
1	17	16	18	18	18	18	19	18	17	17	18	17
7	18	17	19	19	18	19	20	18	18	19	19	17
13	19	17	19	20	19	20	21	19	18	18	19	19
19	21	18	20	20	21	20	22	21	20	20	20	19

Fig. 5l

Ki67

	OCa1			OCa2			OCa9			OCa14		
Vehicle	59.24	61.9	73.49	42.55	43.82	59.68	70.97	71.56	73.6	77.39	79.02	79.46
EC359	30.3	29.44	32.23	17.79	14.77	13.84	13.79	14.12	13.87	26.67	28.7	23.35

Fig. 6a

CD45	Vehicle						EC359					
Tumor infiltrating cells	19.2	6.8	5.1	1.3	1.8	3.98	89	57	70	93	80	30.6

Fig. 6b

tumor cell death	Vehicle								EC359							
GFP+ cells	53	55	56	57	42	47	72	41	88	90	74	99.1	98.5	97.3	90.3	91.4

Fig. 6c

Tumor CD3

	Vehicles								EC359							
Tumor	13.46154	5.936073	15.38462	19.11765	19.64286	3.225806	8.163265	3.225806	17.3913	23.23944	19.68504	28.93082	29.82456	16.66667	11.22449	21.51899
Lymph node	37.37374	36.45833	40.625	42.85714	50	42.26804	46.46465	41.48936	41.05263	33.67347	40.625	53.125	38.20225	35.86957	37.63441	40.42553

Tumor CD8	Vehicle								EC359							
Tumor	10.8	19.8	7.44	4.71	11	24.2	4.66	17.3	11	22	24	29	17.2	25	22.9	39.3
Lymph node	31	24	24	22	24	29	22	24	21	25	25	26	22	23	17	17

Fig. 6d

Tumor B220-CD11bhi myeloid cells

	Vehicle								EC359							
Tumor	17.8	22.9	19.1	6.34	9.16	5.59	9.06	30.1	3.1	16	6.01	3.58	4.2	8.56	6.93	4.64
Lymph node	1.59	1.23	1.15	3.98	1.65	1	0.96	1.26	1.34	0.92	0.96	1.22	1.14	1.05	1.37	1.27

Tumor CD11b-B220+ B cells

	Vehicle								EC359							
Tumor	2.4	9.2	8.95	3.09	6.62	8.3	1.2	4.1	5.1	3.5	5.8	4.6	8.9	8.8	3.4	4.9
Lymph node	33.7	49.4	43.5	36.8	35.8	43.1	48.4	31.3	39.1	31.5	38.5	44	45.9	41.6	37.3	33.3

Fig. 6e
TumorAscites Cd11bhi
pSTAT1hicMAFlo

	Vehicle						EC359					
Tumor	2.1	0	0.58	0	0	4.35	14.6	12.8	23	8.89	15.9	4.76
Ascites	0.32	0.54	0.091	0.76	0.33	0.51	0.88	0.55	0.049	0.051	0.35	1.31

TumorAscites Cd11bhi
pSTAT1locMAFhi

	Vehicle						EC359					
Tumor	42.9	24.7	23.8	60.9	15.8	30.4	3.96	17.4	3.86	0.48	4.73	25
Ascites	33.8	27.2	69	54.1	44.8	56.3	22.9	47.7	35.4	17.6	17	21.7

Fig. 6f
TumorAscites Cd11bhi
cMAFhiPD-L1hi

	Vehicle						EC359					
Tumor	39	20.5	77.9	16.3	42.9	65.7	18.9	11.2	7.61	14.8	21.1	6.13
Ascites	53.9	40	51	25	40.3	15.7	11	28.6	11.1	16.5	17.2	10.4

TumorAscites Cd11bhi
cMAFhiPD-L1lo

	Vehicle						EC359					
Tumor	4.79	24.7	9.05	9.67	20	7.1	0.33	3.7	2.03	1.03	2.74	0.3
Ascites	6.42	3.84	18.3	1.46	5.61	2.84	0.4	1.63	0.12	1.8	0.3	2.06

Fig. 6g

Myeloid cells Cd11bhi

	Vehicle						EC359					
Ascites	11.8	17.8	12.7	10.4	16.6	13.5	7.52	6.83	6.59	6.95	6.33	12.5
Spleen	0.71	0.93	0.49	1.06	1.39	1.29	0.97	2.97	2.11	1.56	1.28	0.59

Cd11bhi subset M-MDSC
Gr1int Ly6G+

	Vehicle						EC359					
Ascites	90.2	89.6	76.2	90.9	86.9	72.4	67.4	59.2	64.7	48.5	64.1	54.7
Spleen	12.1	12.1	10	14.6	11.5	11.1	6.86	4.52	12.2	9.81	8.35	14.5

Cd11bhi subset PMN MDSC
Gr1lhiLy6Ghi

	Vehicle						EC359					
Ascites	0.26	0.37	0.39	0.92	1.96	3.75	0.33	6.41	3.55	3.3	1.72	3.44
Spleen	4.72	6.46	7.18	5.34	3.22	2.88	6.44	22.7	8.69	7.27	1.77	10.9

Cd11bhi subset macrophages
Gr1lhiLy6G-

	Vehicle						EC359					
Ascites	1.07	1.71	2.41	0.96	2.04	8.65	1.94	4.83	2.74	5.58	4.32	5.81
Spleen	29.3	37.2	29.4	30.8	41.8	42.1	43	52.8	38.6	45	43.3	27.3

Cd11bhi subset unknown
Gr1l-Ly6G-

	Vehicle						EC359					
Ascites	7.88	7.86	20.2	6.51	7.97	13.8	28.9	26.6	27.2	39.9	27.8	32.5
Spleen	46.9	37.8	47.3	42.7	37.2	38.1	39.2	12.8	34	33.1	41.6	40.6

Fig. 7a

Days after treatment	Vehicle						EC359					
0	52.65	54.21	50.78	80	118.8	115.35	56.45	33.6	79.33	94.77	79.77	83.82
4	228.27	194.4	143.36	186.2	292.78	262.4	89.38	145.93	162.73	173.78	150.28	126.85
7	294.4	384.17	265.6	249.44	282.91	320.34	100.35	212.54	264.38	251.98	240.12	269
11	732.74	798.06	349.27	530	601.86	454.07	145.41	348.48	425.25	310.46	242.59	327.65
14	974.68	1182.76	382.93	793.5	1016.45	944.34	237.16	325.42	535.55	322.75	363.97	485
18	1314.64	1566.36	876.1	1228.8	1923.79	1744.28	306.56	504.75	746.81	814.09	458.4	773.66
21	1923.2	2326.5	1013.64	1274.88	2157.54	1977.22	363.38	1045.57	900.42	807.91	624.26	1156.55
25	2736.11	3251.38	1327.1	1742.78	3077.85	2664.29	681.41	1273.61	849.14	901.55	884.35	1449.08

Fig. 7b

Tumor weight

Vehicle	EC359
1.618	0.385
1.776	0.629
0.912	0.717
1.089	0.626
1.806	0.576
1.492	0.724

Fig. 7c

Days after treatment	Vehicle					EC359				
0	19	20	19	18	17	19	17	18	20	17
4	19	21	20	19	17	19	18	18	20	18
7	20	20	20	19	17	19	19	18	21	18
11	20	20	21	20	18	18	17	18	20	18
14	19	20	21	20	18	18	18	18	19	18
18	20	20	21	21	18	19	18	18	21	19
21	20	20	22	20	17	19	18	18	19	18
25	20	20	22	21	17	19	18	18	19	19

Fig7d

	Vehicle							EC359						
0	199.8375	163.35	285.8935	289.338	141.5375	176.157	396.9	122.4	75.843	120.9325	171.5	154.8705	273.78	157.115
3	387.2	371.712	196	387.2	358.706	589.8375	348.174	111.392	80.0565	137.216	110.808	159.3595	159.744	108
7	1283.854	1225.804	651.688	899.2235	874.64	1333.08	976.5625	292.032	228.528	665.5	240.4305	603.432	726.7165	265.7205
10	2086.641	1873.95	1533.042	2246.063	2304.682	2342.26	1983.384	618.3832	484.416	1340.96	715.084	1081.724	1127.844	545.7535

Fig. 7e
Tumor weight

Vehicle	EC359
1.54	0.56
1.42	0.35
1.01	0.63
1.3	0.64
1.28	0.81
1.36	0.66
1.1	0.3

Fig. 7f

body weight		Vehicle				EC359			
	0	18	18	18	19	18	17	16	16
	3	18	18	20	19	18	17	16	16
	7	18	19	20	20	18	16	17	16
	10	19	20	21	20	19	18	18	17

Fig. 7g

Days after treatment	Vehicle								EC359							
0	233.93	92.7	141.54	77.25	153.6	184.32	181.3	184	222.16	160.55	101.4	92.26	106.17	120.6	136.8	176.42
4	303.24	206.68	102.6	127.8	202.55	194.48	310.46	406.27	196.46	87.48	152.1	107.91	124.2	134.95	138.6	167.71
7	333.04	215.6	156.82	98.31	175.71	256.61	305.94	404.6	247.5	150.82	206.68	78.73	82.87	93.31	202.34	142.88
12	441.8	285.95	180.59	211.72	405.77	482.45	451.25	325.82	302.69	246.42	172.65	109.8	115.2	160.74	252.33	261.61
15	509.28	368	287.71	233.93	392.09	529.92	437.38	309.08	330.77	309.3	135.82	122.4	136.93	93.64	284.06	213.15
19	596.76	521.32	279.94	220.32	357.64	612.86	550.85	580.61	317.2	325.13	180.22	97.56	124.65	257.03	266.81	186.29
26	640.45	545.72	320.57	282.12	428.69	732.02	720.78	686.82	340.13	473.81	222.21	198.45	183.75	239.7	365.1	299.57
33	1017.55	990.98	499.55	482.45	652.45	1010.32	1139.91	718.85	484.44	617.46	346.56	132.62	314.41	402.69	480.63	403.97
39	899.43	1242.19	561.09	389.23	805.75	1123.2	1101.42	719.79	575.16	577.37	423.77	181.3	465.83	345.6	563.11	478.33

Fig. 7h

Vehicle	EC359
0.556	0.506
0.554	0.327
0.561	0.186
0.304	0.116
0.392	0.163
0.482	0.104
0.548	0.222
0.399	0.3

Fig. 7i

Days after treatment	Vehicle					EC359				
0	21	19	22	22	21	21	20	23	20	19
4	22	19	23	22	21	21	21	23	20	19
7	22	20	24	23	21	21	21	23	20	19
12	23	20	24	22	22	21	20	23	20	20
15	23	20	25	22	22	21	21	24	19	19
19	23	20	25	22	22	21	21	23	20	20
26	23	20	25	22	22	22	23	24	21	21
33	24	22	25	24	23	21	23	25	21	21
39	24	22	22	23	22	23	23	25	22	21

Fig. 7j

Days after treatment	Vehicle						EC359					
0	128.37	83.82	123.46	124.93	71.66	138.38	100.95	88.77	106.17	161.84	87.81	106.17
4	166.09	267.33	206.68	99.09	166.64	137.31	125.32	93.99	113.4	231.23	118.8	121.09
8	268.36	216.56	208.09	171.4	156.82	135.17	137.5	195.27	83.19	219.49	141.31	166.64
11	447.17	510	346.8	258.57	222.38	189.22	146.2	330.67	120.09	258.57	166.46	171.09
15	658.32	707.85	322.75	210.94	272.73	137.31	167.45	296.45	90.94	251.98	233.93	261.61
21	1381.21	951.67	462.4	536.99	491.86	678.98	250.47	607.5	120.09	407.93	314.93	575.24
24	1965.84	1120.97	468.51	435.22	571.44	1149.32	405.67	740.05	109.65	471.3	393.35	672.3
28	2458.95	1352.47	600.85	552.25	651.69	2250.01	459.27	861.98	107.91	506.25	402.18	986.12

Fig. 7k

Vehicle	EC359
1.073	0.169
0.635	0.546
0.435	0.046
0.305	0.232
0.631	0.173
0.501	0.204

Fig. 7l

Body weight						
	Vehicle			EC359 (5 mg/kg body wt.)		
0	20	20	22	19	22	20
4	21	20	22	19	22	18
8	22	21	23	19	22	20
11	21	20	24	19	21	20
15	22	20	23	20	21	19
21	22	20	23	20	23	19
24	23	20	24	20	23	19
28	22	20	24	21	23	19
