# Supplementary material for: Pharmacological inhibition of the LIF/LIFR autocrine loop reveals vulnerability of ovarian cancer cells to ferroptosis
Source: NPJ Precis Oncol. 2024 May 24;8:118. doi: 10.1038/s41698-024-00612-y (PMC11126619; doi:10.1038/s41698-024-00612-y)
Supplement: Supplementary file 2 — Reporting summary [file 41698_2024_612_MOESM2_ESM.pdf]

nature portfolio

Corresponding author(s): Ratna K Vadlamudi
Hareesh Nair
Suryavathi Viswanadhapalli

Last updated by author(s): 3/14/2024

Reporting Summary

Nature Portfolio wishes to improve the reproducibility of the work that we publish. This form provides structure for consistency and transparency in reporting. For further information on Nature Portfolio policies, see our Editorial Policies and the Editorial Policy Checklist.

Statistics

For all statistical analyses, confirm that the following items are present in the figure legend, table legend, main text, or Methods section.

n/a	Confirmed	
☐	☒	The exact sample size (*n*) for each experimental group/condition, given as a discrete number and unit of measurement
☐	☒	A statement on whether measurements were taken from distinct samples or whether the same sample was measured repeatedly
☐	☒	The statistical test(s) used AND whether they are one- or two-sided<br>*Only common tests should be described solely by name; describe more complex techniques in the Methods section.*
☒	☐	A description of all covariates tested
☐	☒	A description of any assumptions or corrections, such as tests of normality and adjustment for multiple comparisons
☐	☒	A full description of the statistical parameters including central tendency (e.g. means) or other basic estimates (e.g. regression coefficient) AND variation (e.g. standard deviation) or associated estimates of uncertainty (e.g. confidence intervals)
☐	☒	For null hypothesis testing, the test statistic (e.g. *F*, *t*, *r*) with confidence intervals, effect sizes, degrees of freedom and *P* value noted<br>*Give P values as exact values whenever suitable.*
☒	☐	For Bayesian analysis, information on the choice of priors and Markov chain Monte Carlo settings
☒	☐	For hierarchical and complex designs, identification of the appropriate level for tests and full reporting of outcomes
☒	☐	Estimates of effect sizes (e.g. Cohen's *d*, Pearson's *r*), indicating how they were calculated

Our web collection on statistics for biologists contains articles on many of the points above.

Software and code

Policy information about availability of computer code

Data collection	Bio-Rad microplate reader was used for MTT assays<br>Bio-Rad imager was used for to collect western blotting data<br>The raw reads were aligned to the reference human genome (UCSC hg19) with TopHat2 . Genes were annotated (using NCBI RefSeq) and quantified by HTSeq , and DESeq was used to identify differentially expressed genes and significant genes with fold change > 1 and multiple-test adjusted p value <0.01 were used for interpreting the biological pathways.
Data analysis	Graphpad Prism (versions 9) was used for statistical analyses<br>Image Lab 5.2.1 (Bio-Rad) was used to analyze Western Blotting data.<br>FACS data were analyzed by the FlowJo®software (version 10)<br>TNM plot analysis tool (https://tnmplot.com/analysis/) to examine alterations in the levels of LIF, LIFR, and OSM in OCa,<br>ROC plotter was utilized to compare the expression levels of chemotherapy-resistant and sensitive malignancies<br>ELDA was performed using https://bioinf.wehi.edu.au/software/elda/

For manuscripts utilizing custom algorithms or software that are central to the research but not yet described in published literature, software must be made available to editors and reviewers. We strongly encourage code deposition in a community repository (e.g. GitHub). See the Nature Portfolio guidelines for submitting code & software for further information.

Data

Policy information about availability of data

All manuscripts must include a data availability statement. This statement should provide the following information, where applicable:

- Accession codes, unique identifiers, or web links for publicly available datasets
- A description of any restrictions on data availability
- For clinical datasets or third party data, please ensure that the statement adheres to our policy

mRNA-seq data are available from NCBI GEO under accession# GSE236743

Research involving human participants, their data, or biological material

Policy information about studies with human participants or human data. See also policy information about sex, gender (identity/presentation), and sexual orientation and race, ethnicity and racism.

Reporting on sex and gender	NA
Reporting on race, ethnicity, or other socially relevant groupings	NA
Population characteristics	NA
Recruitment	NA
Ethics oversight	NA

Note that full information on the approval of the study protocol must also be provided in the manuscript.

Field-specific reporting

Please select the one below that is the best fit for your research. If you are not sure, read the appropriate sections before making your selection.

☒ Life sciences ☐ Behavioural & social sciences ☐ Ecological, evolutionary & environmental sciences

For a reference copy of the document with all sections, see nature.com/documents/nr-reporting-summary-flat.pdf

Life sciences study design

All studies must disclose on these points even when the disclosure is negative.

Sample size	No statistical method was used to predetermine sample size. For in vivo experiments, we used at least 6 mice per group which is sufficient to detect meaningful biological difference. For in vitro experiments, unless otherwise stated, n=2 was chosen as the minimal number of replicates per experiment that would allow for adequate analysis to draw meaningful conclusions. We determined this to be sufficient based on the low observed variability between samples from in vitro experiments.
Data exclusions	Animals were excluded if the implanted tumors did not take
Replication	All in vitro assays were performed in triplicate and repeated at least two time
Randomization	Animal groups were randomized based on tumor volume. All in vitro assays were done in triplicate with no randomization.
Blinding	Blinding was not used in any experiment in this study since it is not possible based on treatment and general conditions of the samples used.

Reporting for specific materials, systems and methods

We require information from authors about some types of materials, experimental systems and methods used in many studies. Here, indicate whether each material, system or method listed is relevant to your study. If you are not sure if a list item applies to your research, read the appropriate section before selecting a response.

Materials & experimental systems

n/a	Involved in the study
☐	☒ Antibodies
☐	☒ Eukaryotic cell lines
☒	☐ Palaeontology and archaeology
☐	☒ Animals and other organisms
☒	☐ Clinical data
☒	☐ Dual use research of concern
☒	☐ Plants

Methods

n/a	Involved in the study
☒	☐ ChIP-seq
☐	☒ Flow cytometry
☒	☐ MRI-based neuroimaging

Antibodies

Antibodies used

Supplementary Table 2.
List of the antibodies used for western blot analysis
mTOR Cell Signaling 2972S
p-mTOR (2448)(49F9) IHC specific Cell Signaling 2976S
p70 S6 Kinase Antibody Cell Signaling 9202
phospho-p70 S6 Kinase Cell Signaling 9205
AKT Cell Signaling 9272S
p-AKT(S473) Cell Signaling 4060S
p-STAT3 (Y705) Cell Signaling 9131S
STAT3 79D7 Cell Signaling 4904S
p44/42MAPK (total) Cell Signaling #9102
Phos-p44/42MAPK Cell Signaling 9101
S6 ribosomal protein Cell Signaling 2217
p-S6 ribosomal Prottein Cell Signaling 4858S
LIF Santa Cruz Biotechnology sc-515931
LIFR Santa Cruz Biotechnology sc-659
Cas9 Cell Signaling 14697
GPX4 Cell Signaling 52455
NRF2 Cell Signaling 12721
xCT/SLC7A11 Cell Signaling 12691
p27 Santa Cruz Biotechnology SC-528
p21 Cell Signaling 2947P

Supplementary Table 4. Monoclonal antibodies to murine antigens and reagents for flow cytometry.
Fluorochrome-conjugated monoclonal antibodies (mAbs) to murine (mu) antigens
a–muCD45-Pacific Blue™ BioLegend Cat. # 103126 (Clone 30-F11)
a–muCD45-biotin BioLegend Cat. # 103104 (Clone 30-F11)
a–muCD3-APC-Cy7 BioLegend Cat. # 100221 (Clone 17A2)
a–muCD8b-PE eBioscience Cat. # 25-0083-82 (Clone H35-17.2)
a–muCD11b-APC BioLegend Cat. # 101212 (Clone M1/70)
a–muCD11b-biotin BioLegend Cat. # 101204 (Clone M1/70)
a–muB220-APC BioLegend Cat. # 103212 (Clone RA3-6B2)
a–muCD80-PE BD Biosciences Cat. # 553769 (Clone 16-10A1)
a–muPhosphoSTAT1 (Tyr701)-PE Cell Signaling Cat. # 8062 (Clone 58D6)
a–mucMAF-PerCP-eFluor™710 eBioscience Cat. # 46-9855-42 (Clone sym0F1)
a–muPD-L1-PerCP-eFluor™710 ThermoFisher Cat. # 46-5983-42 (Clone MIH5)
a–muPD-L1-Brilliant Violet 421™ BioLegend Cat. # 124315 (Clone 10F.9G2)
a–muLy6G-Pacific Blue™ BioLegend Cat. # 127612 (Clone 1A8)
a–muGr-1-APC BioLegend Cat. # 108412 (Clone RB6-8C5)
Other reagents
Fixable Viability Dye eFlour™ 780 eBioscience Cat. # 65-0865-14
Fixable Viability Dye eFlour™ 506 eBioscience Cat. # 65-0866-14
7-AAD (7-Aminoactinomycin D) SigmaAldrich Cat. # A9400
APC-Streptavidin BioLegend Cat. # 405207
PE/Cy7-Streptavidin BioLegend Cat. # 405206

Validation

All other antibodies used in this study were validated by manufacturers for that specific application (Western blotting, immunohistochemistry, immunefluorescence and flow cytometry). Relevant validating results can be found in the website of each manufacturer.

Eukaryotic cell lines

Policy information about cell lines and Sex and Gender in Research

Cell line source(s)

OVCAR3 (HGSOC), OVCAR8 (LGSOC), ES2 (LGSOC), SKOV3 (CCOC), TOV21G (CCOC), TOV112D (ENOC), OV90 (MOC), COV644

Cell line source(s)	(MOC), HEK-293T cells were received from the American Type Culture Collection (ATCC, Manassas, VA)<br>OVSAHO (HGSOC) was purchased from AcceGen™<br>OVCAR5 (HGSOC) cell line was purchased from Sigma<br>IGROV1 (ENOC) cell line was procured from Dr. Sood (MD Anderson Cancer Center, Houston).<br>ID8agg procured from Dr. Tyler curiel, Dartmouth medical school<br>OCa1, OCa2, OCa9, OCa10, OCa14, OCa27, OCa30, OCa38, OCa39, OCa45, OCa50, OCa66, and OCa73, AS20, 21, 23, 25, 28, and 29 and HESC cells were obtained from the Ob/Gyn tissue core
Authentication	Authentication was performed by STR profiling in ATCC.
Mycoplasma contamination	All cell lines tested and negative for mycoplasma contamination.
Commonly misidentified lines<br>(See ICLAC register)	None

Animals and other research organisms

Policy information about studies involving animals; ARRIVE guidelines recommended for reporting animal research, and Sex and Gender in Research

Laboratory animals	C57BL6, Female mouse, 6-8 weeks age<br>SCIDmice, Female mouse, 6-8 weeks age<br><br>We followed the UT Health San Antonio humane end points determination policy in our animal studies. None of the mice in our study showed any signs of moribund distress or had signs of unalleviated pain or distress during the experimental duration. None of the mice in our studies died because of the tumor burden. The goal per our approved protocol is to monitor experimental animals and sacrifice mice before the pathologies that may develop before debilitation or when the tumor size reaches ~2000 mm3 in diameter. The 2000 mm3 size criteria was chosen as the earliest humane end-point at which we could get meaningful data from these animal studies: based on prior experience, the tumors would have to get considerably larger to meet IACUC moribund distress criteria. For these studies, we constantly evaluate the size of the tumors based on growth rates and plan for euthanasia when the tumor size reaches ~2000 mm3 in diameter.<br><br>In 4 out of 9 experiments presented in this paper, the mice were euthanized before the tumors reached 2000 mm3. In each of these studies, the decision was made to perform euthanasia using isoflurane inhalation followed by cervical dislocation close to the time when then tumors were reaching the target size. However, in 3 PDX and 2 CDX tumor studies, some of the mice in the vehicle treated controls exceeded our goal of 2000 mm3 size, due to unexpected, unpredictable and rapid growth rate of their tumors. In each case, when the tumor volumes were >2000 mm3, we made the determination that the mice had reached the endpoint criteria and scheduled euthanasia. However, since euthanasia scheduling typically took 1-4 days, inevitably tumors continued to grow rapidly, reaching larger sizes at the time of sacrifice. In each case, we followed the humane endpoint guidelines as per IACUC policy. All the mice were active and none of these mice showed any signs of moribund distress and weight loss during the entire experimental duration. Thus, while we exceeded the goal of 2000 mm3, we were in compliance with the UT Health San Antonio humane end points determination policy for each of these mice.
Wild animals	No wild animals were used in the study
Reporting on sex	Only female mice were used because OCa only occur in females
Field-collected samples	No field collected samples were used in the study.
Ethics oversight	Animal protocol 20220037AR approved by IACUC office in UTHSCSA.

Note that full information on the approval of the study protocol must also be provided in the manuscript.

Plants

Seed stocks	NA
Novel plant genotypes	NA
Authentication	NA

Flow Cytometry

Plots

Confirm that:

☒ The axis labels state the marker and fluorochrome used (e.g. CD4-FITC).

☒ The axis scales are clearly visible. Include numbers along axes only for bottom left plot of group (a 'group' is an analysis of identical markers).

☒ All plots are contour plots with outliers or pseudocolor plots.

☒ A numerical value for number of cells or percentage (with statistics) is provided.

Methodology

Sample preparation

Lymphocyte preparation and analysis by flow cytometry
Single cell suspensions were prepared from pooled and minced ID8 tumors, ascites, draining mesenteric lymph node and the spleen using a 70-um cell strainer. Cells were collected in RPMI 1640 medium (Invitrogen) supplemented with FBS (10% v/v, Invitrogen), penicillin-streptomycin/amphotericin B (1% v/v) and resuspended in ACK Lysis Buffer (Lonzo) to lyse red blood cells. After quenching with RPMI-FBS, cells were resuspended in PBS for immediate staining and flow cytometry analysis. In most experiments involving tumor-infiltrating lymphocytes, leukocytes from ID8 tumors underwent additional enrichment by a Ficoll-Paque gradient (GE Healthcare, Cat. # 17-0891-01) following the manufacturer's instructions.
For staining, cells were first stained for 20 m in Hank's Buffered Salt Solution plus 0.1% BSA (BSA-HBSS) with fluorochrome-labeled mAbs to surface markers in the presence of mAb Clone 2.4G2, which blocks FcgIII and FcgII receptors, and 7-AAD or a fluorochrome-conjugated fixable viability dye (FVD, Supplementary Table 4). After washing, cells were either resuspended in HBSS for FACS analysis in an LSRII (BD) or proceeded for intracellular staining of phosphorylated STAT1 (pSTAT1) and cMAF. For intracellular staining, cells were resuspended in the BD Cytofix/Cytoperm buffer (250 ul) and incubated at 4°C for 20 m. After washing twice with the BD Perm/Wash buffer, cells were counted again and 1 million cells were resuspended in 100 ul of BD Cytofix/Cytoperm buffer for staining with fluorochrome-labeled anti-pSTAT1 and/or anti-cMAF mAbs at 4°C for 30 m (Supplementary Table 4). After washing with BD Perm/Wash buffer, cells were analyzed by FACS in an LSRII. All data were analyzed by FlowJo® (BD).

Instrument

LSRII (BD)

Software

FlowJo® (BD)

Cell population abundance

The numbers within each plot indicate the proportions (relative abundance) of indicated cells or cell subsets within the cell populations in the previous step.

Gating strategy

Fig. 6A, B: SSC-A/FSC-A lymphocyte ->FVD- live cell ->CD45+ (Supplementary Fig.5A)
Fig. 6C: FVD- live cell ->CD3+CD45+ T cell -> CD8+ T cell (Supplementary Fig.5B)
Fig. 6D: CD45+ ->B220 and CD11b (Supplementary Fig.5C)
Fig. 6E: FVD- live cell ->CD11b+ -> p-STAT1 and cMAF (Supplementary Fig.5D)
Fig. 6F: FVD- live cell ->CD80+CD11b+ ->cMAF and PD-L1 (Supplementary Fig.5E)
Fig. 6G: FVD- live cell ->cMAF and PD-L1 (Supplementary Fig.5E)
FVD- live cell ->CD80+CD11b+ -> Gr-1 and Ly6G (Supplementary Fig.5F)

☒ Tick this box to confirm that a figure exemplifying the gating strategy is provided in the Supplementary Information.
